# Dietary indices to measure diet quality in older cancer survivors: A scoping review on tools, their components and association with health outcomes

**DOI:** 10.1016/j.archger.2025.105797

**Published:** 2025-02-18

**Authors:** Andrea Boehmer, Christina Syu Hong Thio, Juliana Christina, Michelle Miller, Alex Fauer, Elsa Dent, Wendy Wing Tak Lam, Danielle Wing Lam Ng, Raymond Javan Chan, Chad Yixian Han

**Affiliations:** a Caring Futures Institute, College of Nursing and Health Sciences, Flinders University, Adelaide, South Australia, Australia; b Betty Irene Moore School of Nursing, University of California Davis, Sacramento, California, USA; c Faculty of Health Sciences & Medicine, Bond University, Gold Coast, Queensland, Australia; d LKS Faculty of Medicine, School of Public Health, Centre for Psycho-Oncology Research and Training, The University of Hong Kong, Hong Kong, Hong Kong SAR, China; e LKS Faculty of Medicine, Jockey Club Institute of Cancer Care, The University of Hong Kong, Hong Kong, Hong Kong SAR, China

**Keywords:** Diet, Healthy*, Aged, Cancer survivors, Nutrition assessment, Diet surveys/methods*

## Abstract

**Background:**

Older cancer survivors live with more comorbidities and have a higher mortality rate compared to the general older population. A high-quality diet that adheres to evidence based dietary recommendations and guidelines may help mitigate these issues. This can be assessed using dietary quality indices (DQIs), which objectively summarize scores for selected dietary components.

**Objective:**

Identify the DQIs available in the literature for older cancer survivors, and their associations with health outcomes.

**Method:**

Five databases were searched to identify peer-reviewed articles in English, from inception to 12th November 2024. Two researchers independently screened 3,145 studies, extracted and qualitatively assessed data from 28 included reports from 16 studies.

**Results:**

12 DQIs and 40 unique components within these indices were identified and summarised narratively. Total vegetables (*n* = 8), total fruits (*n* = 8), whole grains (*n* = 6), saturated fat (*n* = 8), and salt/sodium (*n* = 8) were the most frequently incorporated components within a DQI. All DQIs were derived from evidence-based dietary guidelines. Only three DQIs were specifically designed for oncology population. Higher diet quality was associated with higher HR-QoL in breast, prostate, and colorectal cancer survivors in all but one study. The associations between mortality and diet quality were inconsistent, depending on the type of cancer and the mortality type i.e., cancer-specific or other causes.

**Conclusions:**

DQIs are associated with important health outcomes. A major knowledge gap exists in DQIs suitable for older cancer survivors. Future research should develop DQIs to better assess how high-quality diets enhance health outcomes in older cancer survivors.

## 1. Introduction

The intersection of aging, nutrition, and cancer carries significant global implications, especially as we are now in the United Nations (UN) Decade of Healthy Ageing (2021–2030) and the UN Decade of Action on Nutrition (2016–2025) (United Nations, 2016; World Health Organization, 2023). Despite these initiatives, a gap remains in research on nutrition among older adults. This is particularly concerning, as recent literature has highlighted that older cancer survivors have a higher mortality rate and are at greater risk of comorbidities, severe health issues and developing second primary malignancies compared to their peers without cancer (Sulicka et al., 2018; Sung et al., 2021; Zheng et al., 2020). Addressing these intersecting factors is crucial for improving health outcomes and quality of life for such vulnerable populations worldwide.

The number of older cancer survivors is rising due to advances in early detection, treatment, and supportive care, which have improved survival rates (World Cancer Research Fund/American Institute for Cancer Research [WCRF/ACIR], 2018). Additionally, an aging population and increased focus on survivorship contribute to the growing number of older cancer survivors (WCRF/ACIR, 2018). Among the domains of supportive care in cancer survivorship, diet and nutrition form an integral part and are reflected in key management guidelines (Rock et al., 2022). Poor dietary habits can potentially exacerbate health problems e.g., sarcopenia, diabetes and hypertension, among older cancer survivors, whereas adopting healthier dietary patterns (i.e., adhering to recommended dietary guidelines) may offer positive health outcomes (Kleckner and Magnuson, 2022).In the wider population of cancer survivors, post-diagnostic diet quality was also associated with all-cause and cancer-specific mortality (Long et al., 2022; Park et al., 2022).With the rising number of older cancer survivors and the potential impact of diet on long-term health outcomes, assessing diet quality becomes increasingly important to understand and support their unique nutritional needs.

When examining the diet, measuring intake of specific nutrients can be pertinent to prevent and manage conditions related to deficiencies, while reviewing the overall diet through food-based perspective can assess how well an individual’s diet aligns with key recommendations and dietary guidelines from peak health organizations, e.g., World Cancer Research Fund/American Institute for Cancer Research (WCRF/ACIR, 2007; WCRF/ACIR, 2018). Diet quality assessment focuses on evaluating the diet in its entirety, focusing on not just specific important nutrients but food groups, allowing for cross-examination with health outcomes rather than solely focusing on any specific individual nutrients (Wirt and Collins, 2009).

Diet quality measurement has evolved, and the utilization and investigation of diet quality using predefined diet quality indices among cancer survivors have increased (Schwedhelm et al., 2016). Diet quality indices (DQIs) allow for assessing dietary quality in a population based on established guidelines, considering the positive or negative effects of various components in the overall diet (Schwedhelm et al., 2016). Depending on the a priori defined set of dietary guidelines, a quantitative score will be assigned to measure the individual’s level of adherence to the recommended diet. Furthermore, DQIs offer the advantage of examining the entire diet rather than focusing on individual nutrients and considering their interactions, for both research and clinical purposes (Kranz et al., 2022; Schwedhelm et al., 2016).

The significance of a high-quality diet for older cancer survivors highlights the importance of accurately assessing diet quality in this population and how it can affect their associations to health outcomes. As the population of older cancer survivors grows, understanding and addressing their unique nutritional needs and diet quality evaluation becomes increasingly important. The aims of this scoping review were to identify such DQIs used in the literature, map out the dietary components that make up these DQIs, and summarize the associations of such DQIs with health outcomes in this vulnerable population.

## 2. Methods

This study followed the Preferred Reporting Items for Systematic Reviews and Meta-Analyses Extension for Scoping Reviews (PRISMA-ScR), in Supplementary materials, Table S1 (Tricco et al., 2018). The study protocol was prospectively documented in the Open Science Framework Registry – https://osf.io/5d9em. The search strategy (Supplementary materials, Table S2) was developed by the lead authors (CYH and AB) and two other reviewers (MM and RJC) with the assistance of an academic librarian. Using a search strategy that consisted of agreed keywords and vocabulary terms, the academic librarian conducted systematic searches of peer-reviewed literature across five electronic databases (Medline, CINAHL, Scopus, Cochrane CENTRAL, and Web of Science) from inception to 12th November 2024 (Table S4). In addition, reference lists of eligible full-text articles were screened.

Peer-reviewed journal articles written in English were included if they 1) participants were older people with cancer conducted in humans aged ≥65 years (mean or median age of cohort ≥65 years), 2) used whole food or nutrients as a measure of diet quality, and 3) were quantitative in nature. Studies were excluded if they were qualitative only, reviews, or focused on a singular therapeutic function or particular food group. Relevant information from the selected studies including author (s), country, year of publication, population characteristics (mean age, standard deviation, sex, cancer type), dietary intake assessment, evidence-based guidelines used for DQI development, scoring and weighting of components, interpretation of score, and evaluation were extracted from each article into an excel spreadsheet and tabulated.

Food and nutrient components of each diet quality index were extracted; typically, they were categorized as either adequacy or moderation components. The adequacy components focus on foods that provide essential nutrients and promote overall health, supporting bodily functions and disease prevention. In contrast, the moderation components highlight foods that should be limited or consumed sparingly, as excessive intake may lead to negative health outcomes such as obesity, heart disease, and other chronic conditions.

## 3. Results

**Fig. 1.**
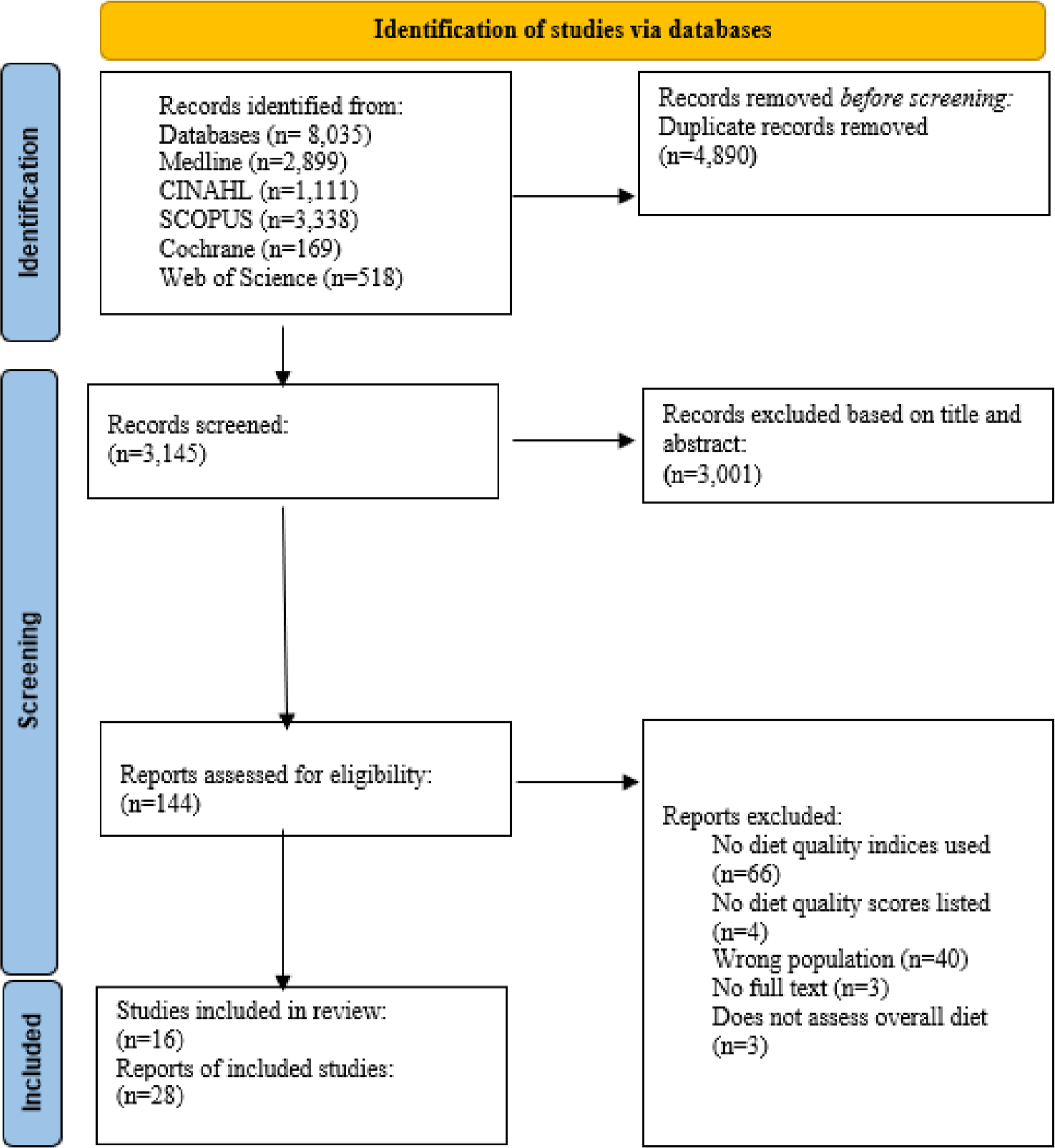
PRISMA 2020 flow chart illustrating results of the search and study screening and selection process.

The initial search in the five databases yielded 8035 records. After removing duplicates, 3145 remained, of which 3001 were excluded following title and abstract screening. Of the 144 full-text articles assessed for eligibility, 116 were excluded (Supplementary materials, Table S3). A total of 28 articles from 16 studies met the criteria were included in the narrative synthesis (Fig. 1).

### 3.1. Characteristics of sampled populations in reviewed studies

All but one study included in this review were from high-income countries, except the BHEI-R, (Mazzutti et al., 2021) which was adapted for the Brazilian population based on the HEI-2005 (Table S1). The DQIs were mainly developed for populations within the United States (US) (*n* = 21) (Bauer et al., 2018; Clutter Snyder et al., 2007; Demark-Wahnefried et al., 2006; Demark-Wahnefried et al., 2003; Demark-Wahnefried et al., 2012; Gopalakrishna et al., 2018; Inoue-Choi et al., 2014; Inoue-Choi et al., 2013a; Inoue-Choi et al., 2013b; Klassen et al., 2018; Krok-Schoen et al., 2021; McCullough et al., 2016; Miller et al., 2008; Mosher et al., 2009; Pelser et al., 2014; Pisegna et al., 2021; Schmalenberger et al., 2022; Snyder et al., 2009; Song et al., 2021; Sun et al., 2018; Wang et al., 2021) and the Netherlands (*n* = 5) (Breedveld-Peters et al., 2018; Kenkhuis et al., 2021; Koole et al., 2020; van Veen et al., 2019; van Zutphen et al., 2019). Sample size for all included studies ranged from 40 (Bennett et al., 2024) to 5427 (Pelser et al., 2014) older cancer survivors. The mean or median age of the included studies was between 65.0 to 78.9 years. Further details can be found in the online supplementary materials (Table S4).

Fourteen articles (Bauer et al., 2018; Breedveld-Peters et al., 2018; Gopalakrishna et al., 2018; Kenkhuis et al., 2021; Koole et al., 2020; Mazzutti et al., 2021; McCullough et al., 2016; Pelser et al., 2014; Pisegna et al., 2021; Song et al., 2021; Sun et al., 2018; van Veen et al., 2019; van Zutphen et al., 2019; Wang et al., 2021) focused on one cancer type e.g., breast, while the rest (Bennett et al., 2024; Clutter Snyder et al., 2007; Demark-Wahnefried et al., 2006; Demark-Wahnefried et al., 2003; Demark-Wahnefried et al., 2012; Inoue-Choi et al., 2014; Inoue-Choi et al., 2013a; Inoue-Choi et al., 2013b; Klassen et al., 2018; Krok-Schoen et al., 2021; Miller et al., 2008; Mosher et al., 2009; Schmalenberger et al., 2022; Snyder et al., 2009) included more than one type of cancer e.g., breast and prostate cancer. Overall, the top three most studied cancers were breast (*n* = 17) (Clutter Snyder et al., 2007; Demark-Wahnefried et al., 2006; Demark-Wahnefried et al., 2003; Demark-Wahnefried et al., 2012; Inoue-Choi et al., 2014; Inoue-Choi et al., 2013a; Inoue-Choi et al., 2013b; Klassen et al., 2018; Krok-Schoen et al., 2021; Mazzutti et al., 2021; McCullough et al., 2016; Miller et al., 2008; Mosher et al., 2009; Pisegna et al., 2021; Schmalenberger et al., 2022; Snyder et al., 2009; Sun et al., 2018), colorectal (*n* = 14) (Breedveld-Peters et al., 2018; Demark-Wahnefried et al., 2012; Inoue-Choi et al., 2014; Inoue-Choi et al., 2013a; Inoue-Choi et al., 2013b; Kenkhuis et al., 2021; Koole et al., 2020; Miller et al., 2008; Mosher et al., 2009; Pelser et al., 2014; Snyder et al., 2009; Song et al., 2021; van Veen et al., 2019; van Zutphen et al., 2019) and prostate (*n* = 9) (Bauer et al., 2018; Clutter Snyder et al., 2007; Demark-Wahnefried et al., 2006; Demark-Wahnefried et al., 2003; Demark-Wahnefried et al., 2012; Klassen et al., 2018; Miller et al., 2008; Mosher et al., 2009; Snyder et al., 2009). Most of the DQIs were developed using data from cross-sectional studies (*n* = 10) (Bauer et al., 2018; Bennett et al., 2024; Breedveld-Peters et al., 2018; Gopalakrishna et al., 2018; Kenkhuis et al., 2021; Krok-Schoen et al., 2021; Miller et al., 2008; Mosher et al., 2009; Pisegna et al., 2021; Schmalenberger et al., 2022), prospective cohort studies (*n* = 10)(Inoue-Choi et al., 2014; Inoue-Choi et al., 2013a; Inoue-Choi et al., 2013b; Koole et al., 2020; Mazzutti et al., 2021; McCullough et al., 2016; Pelser et al., 2014; Song et al., 2021; Sun et al., 2018; van Zutphen et al., 2019) and randomized controlled trials (*n* = 5) (Clutter Snyder et al., 2007; Demark-Wahnefried et al., 2006; Demark-Wahnefried et al., 2003; Demark-Wahnefried et al., 2012; Snyder et al., 2009).

### 3.2. Summary of dietary quality indices (DQIs) used for older cancer survivors

The following twelve DQIs were summarized from the studies that met the criteria of this scoping review: 1. Healthy Eating Index (HEI) HEI-2005, (Demark-Wahnefried et al., 2012; Mosher et al., 2009; Pelser et al., 2014) 2. HEI-2010,(Gopalakrishna et al., 2018; Klassen et al., 2018; Sun et al., 2018; Wang et al., 2021) 3. HEI-2015,(Krok-Schoen et al., 2021; Pisegna et al., 2021; Schmalenberger et al., 2022) 4. Brazilian Healthy Eating Index (BHEI-R),(Mazzutti et al., 2021) 5. Revised Healthy Eating Index (RHEI), (Miller et al., 2008; Snyder et al., 2009) 6. Diet Quality Index Revised (DQI-R),(Clutter Snyder et al., 2007; Demark-Wahnefried et al., 2006; Demark-Wahnefried et al., 2003) 7. Mediterranean Diet Score (MDS),(Bauer et al., 2018) 8. Dutch recommendations for a Healthy Diet (DHD-Index),(Breedveld-Peters et al., 2018) and the 9-item index (Klassen et al., 2018). Details on the scoring and weighting of components within the indices and interpretation of scores can be found in the supplementary materials (Table S4).

The following three indices that included diet and other lifestyle factors were summarized from the studies that met the criteria of this scoping review: 1. World Cancer Research Fund/American Institute for Cancer Research recommendations WCRF/AICR-2007, (Breedveld-Peters et al., 2018; Inoue-Choi et al., 2014; Inoue-Choi et al., 2013a; Inoue-Choi et al., 2013b; van Veen et al., 2019) 2. WCRF/AICR-2018,(Bennett et al., 2024; Kenkhuis et al., 2021; Koole et al., 2020; Song et al., 2021; van Zutphen et al., 2019) and 3. American Cancer Society guidelines scores (ACS) (McCullough et al., 2016). Details on scoring and weighting of components within the indices and interpretation of scores can be found in supplementary materials (Table S4).

### 3.3. Cancer-specific diet quality indices

Most DQIs (*n* = 9) were non-cancer specific and used in the general older adult population (Bauer et al., 2018; Breedveld-Peters et al., 2018; Clutter Snyder et al., 2007; Demark-Wahnefried et al., 2006; Demark--Wahnefried et al., 2003; Demark-Wahnefried et al., 2012; Gopalakrishna et al., 2018; Klassen et al., 2018; Krok-Schoen et al., 2021; Mazzutti et al., 2021; Miller et al., 2008; Mosher et al., 2009; Pelser et al., 2014; Pisegna et al., 2021; Schmalenberger et al., 2022; Snyder et al., 2009; Sun et al., 2018; Wang et al., 2021). Only three DQIs were found to be specific to the cancer population: WCRF/AICR-2007 (Breedveld-Peters et al., 2018; Inoue-Choi et al., 2014; Inoue-Choi et al., 2013a; Inoue-Choi et al., 2013b; van Veen et al., 2019), WCRF/AICR-2018 (Bennett et al., 2024; Kenkhuis et al., 2021; Koole et al., 2020; Song et al., 2021; van Zutphen et al., 2019) and ACS (McCullough et al., 2016), by measuring adherence to the respective dietary components found within the diet, nutrition, and physical activity guidelines for cancer prevention.

### 3.4. Dietary components within the dietary quality indices

The collective number of unique components included in the DQIs was 40, ranging from three (McCullough et al., 2016) to 13 (Krok-Schoen et al., 2021; Pisegna et al., 2021; Schmalenberger et al., 2022) within an index. Dietary components have been mapped to each DQI presented in Table 1. Eleven (Bauer et al., 2018; Bennett et al., 2024; Breedveld-Peters et al., 2018; Clutter Snyder et al., 2007; Demark-Wahnefried et al., 2006; Demark-Wahnefried et al., 2003; Demark-Wahnefried et al., 2012; Gopalakrishna et al., 2018; Inoue-Choi et al., 2014; Inoue-Choi et al., 2013a; Inoue-Choi et al., 2013b; Kenkhuis et al., 2021; Klassen et al., 2018; Koole et al., 2020; Krok-Schoen et al., 2021; Mazzutti et al., 2021; Miller et al., 2008; Mosher et al., 2009; Pelser et al., 2014; Pisegna et al., 2021; Schmalenberger et al., 2022; Snyder et al., 2009; Song et al., 2021; Sun et al., 2018; van Veen et al., 2019; van Zutphen et al., 2019; Wang et al., 2021) out of the included 12 DQIs encompassed both food groups and nutrients as components; ACS (McCullough et al., 2016) only consisted of food-based components. One DQI (Clutter Snyder et al., 2007; Demark-Wahnefried et al., 2006; Demark-Wahnefried et al., 2003) had components that were neither food nor nutrient-focused; the DQI-R (Clutter Snyder et al., 2007; Demark-Wahnefried et al., 2006; Demark-Wahnefried et al., 2003) included a component each for diet diversity and dietary moderation.

The most common number of components within the DQI was 12; with a median of nine and three food-based and nutrient-based components, respectively.

Several adequacy components (e.g., vegetables, fruits, whole grains, and dairy) and moderation components (e.g., red/processed meat, total fat, saturated fatty acid (SFA), salt/sodium, and added sugar/sugary foods and drinks) were integrated into all DQI measures, each of which comprised at least one such component. Overall, for food-based components, total vegetables (*n* = 8) (Bauer et al., 2018; Breedveld-Peters et al., 2018; Clutter Snyder et al., 2007; Demark-Wahnefried et al., 2006; Demark-Wahnefried et al., 2003; Demark-Wahnefried et al., 2012; Gopalakrishna et al., 2018; Krok-Schoen et al., 2021; Mazzutti et al., 2021; Miller et al., 2008; Mosher et al., 2009; Pelser et al., 2014; Pisegna et al., 2021; Schmalenberger et al., 2022; Snyder et al., 2009; Sun et al., 2018; Wang et al., 2021), total fruit (*n* = 8) (Bauer et al., 2018; Breedveld-Peters et al., 2018; Clutter Snyder et al., 2007; Demark-Wahnefried et al., 2006; Demark-Wahnefried et al., 2003; Demark-Wahnefried et al., 2012; Gopalakrishna et al., 2018; Izano et al., 2013; Klassen et al., 2018; Krok-Schoen et al., 2021; Mazzutti et al., 2021; Miller et al., 2008; Mosher et al., 2009; Pelser et al., 2014; Pisegna et al., 2021; Schmalenberger et al., 2022; Snyder et al., 2009; Sun et al., 2018; Wang et al., 2021), whole grains (*n* = 6)(Clutter Snyder et al., 2007; Demark-Wahnefried et al., 2006; Demark-Wahnefried et al., 2003; Demark-Wahnefried et al., 2012; Gopalakrishna et al., 2018; Klassen et al., 2018; Krok-Schoen et al., 2021; Mazzutti et al., 2021; Miller et al., 2008; Mosher et al., 2009; Pelser et al., 2014; Pisegna et al., 2021; Schmalenberger et al., 2022; Snyder et al., 2009; Sun et al., 2018; Wang et al., 2021) were the most frequently incorporated components within the 12 DQIs, while for nutrient-based components, they were SFA (*n* = 8) (Bauer et al., 2018; Breedveld-Peters et al., 2018; Clutter Snyder et al., 2007; Demark-Wahnefried et al., 2006; Demark-Wahnefried et al., 2003; Demark-Wahnefried et al., 2012; Klassen et al., 2018; Miller et al., 2008; Mosher et al., 2009; Pelser et al., 2014; Snyder et al., 2009) and sodium (*n* = 8) (Breedveld-Peters et al., 2018; Demark-Wahnefried et al., 2012; Gopalakrishna et al., 2018; Klassen et al., 2018; Krok-Schoen et al., 2021; Mazzutti et al., 2021; Miller et al., 2008; Mosher et al., 2009; Pelser et al., 2014; Pisegna et al., 2021; Ryu et al., 2020; Schmalenberger et al., 2022; Snyder et al., 2009; Sun et al., 2018; Wang et al., 2021). Most DQIs (*n* = 8) (Bauer et al., 2018; Breedveld-Peters et al., 2018; Clutter Snyder et al., 2007; Demark-Wahnefried et al., 2003; Demark--Wahnefried et al., 2012; Demark-Wahnefried et al., 2000; Gopalakrishna et al., 2018; Inoue-Choi et al., 2014; Inoue-Choi et al., 2013a; Inoue-Choi et al., 2013b; Klassen et al., 2018; Krok-Schoen et al., 2021; Mazzutti et al., 2021; Miller et al., 2008; Mosher et al., 2009; Pelser et al., 2014; Pisegna et al., 2021; Schmalenberger et al., 2022; Snyder et al., 2009; Sun et al., 2018; van Veen et al., 2019; Wang et al., 2021) either scored diet quality based on a standard energy count (e.g., number of serves per 1000kcal), or by a stand-alone component (e.g., scoring 1 for total calories within a pre-defined range).

Most components within all DQIs were assessed as continuous variables, represented as a percentage (0 to 100 %) indicating adherence to the recommendations. However, some components are scored categorically (Table S1). Four DQIs HEI-2005 (Demark-Wahnefried et al., 2012; Mosher et al., 2009; Pelser et al., 2014) and HEI-2010 (Gopalakrishna et al., 2018; Klassen et al., 2018; Sun et al., 2018; Wang et al., 2021), BHEI-R (Mazzutti et al., 2021), R-HEI (Miller et al., 2008; Snyder et al., 2009)) incorporate components with different weightings e.g. total scores were 10 and 20 for dairy and empty calories, respectively, while the remaining DQIs assigned equal scores to all components. Energy intake is considered for scoring purposes in majority (9 out of 12) of the DQIs (HEI-2005 (Demark-Wahnefried et al., 2012; Mosher et al., 2009; Pelser et al., 2014), HEI-2010 (Gopalakrishna et al., 2018; Klassen et al., 2018; Sun et al., 2018; Wang et al., 2021), HEI-2015 (Krok-Schoen et al., 2021; Pisegna et al., 2021; Schmalenberger et al., 2022), BHEI-R (Mazzutti et al., 2021), R-HEI (Miller et al., 2008; Snyder et al., 2009), DQI-R (Clutter Snyder et al., 2007; Demark-Wahnefried et al., 2006; Demark-Wahnefried et al., 2003), DHD-Index (Breedveld-Peters et al., 2018), WCRF/AICR 2007, (Breedveld-Peters et al., 2018; Inoue-Choi et al., 2014; Inoue-Choi et al., 2013a; Inoue-Choi et al., 2013b; van Veen et al., 2019) WCRF/ACIR 2018 (Bennett et al., 2024; Kenkhuis et al., 2021; Koole et al., 2020; Song et al., 2021; van Zutphen et al., 2019), 9-item index (Klassen et al., 2018). Details on each dietary component can be found in Supplementary materials, Table S5.

### 3.5. Method of dietary data collection for dietary quality assessment

Various methods were employed to assess participants’ diet quality, as outlined in Table S2 (supporting information online). Dietary intake was mainly assessed through a Food Frequency Questionnaire (FFQ). Overall, FFQs (*n* = 17) (Bauer et al., 2018; Bennett et al., 2024; Gopalakrishna et al., 2018; Inoue-Choi et al., 2014; Inoue-Choi et al., 2013a; Inoue-Choi et al., 2013b; Koole et al., 2020; Krok-Schoen et al., 2021; McCullough et al., 2016; Pelser et al., 2014; Pisegna et al., 2021; Schmalenberger et al., 2022; Song et al., 2021; Sun et al., 2018; van Veen et al., 2019; van Zutphen et al., 2019; Wang et al., 2021)., which thirteen (Bauer et al., 2018; Bennett et al., 2024; Gopalakrishna et al., 2018; Inoue-Choi et al., 2014; Inoue-Choi et al., 2013a; Inoue-Choi et al., 2013b; Krok-Schoen et al., 2021; Pisegna et al., 2021; Schmalenberger et al., 2022; Song et al., 2021; Sun et al., 2018; van Zutphen et al., 2019; Wang et al., 2021)were validated before use. Number of items included in FFQs ranged from 40 (van Veen et al., 2019) to 204 (van Zutphen et al., 2019), with a median of 124 items. Most studies used one FFQ. Three studies (Breedveld-Peters et al., 2018; Kenkhuis et al., 2021; Koole et al., 2020) used food diaries assessing seven-day dietary intake. Nine studies (Clutter Snyder et al., 2007; Demark-Wahnefried et al., 2006; Demark-Wahnefried et al., 2003; Demark-Wahnefried et al., 2012; Klassen et al., 2018; Mazzutti et al., 2021; Miller et al., 2008; Mosher et al., 2009; Snyder et al., 2009) used 24-h dietary recalls, of which three (Demark-Wahnefried et al., 2012; Miller et al., 2008; Mosher et al., 2009) were unannounced. Most 24 h dietary recalls were collected twice, with one study that collected a total of nine repeated recalls (Mazzutti et al., 2021). Additionally, assessments were made concerning sociodemographic factors (Klassen et al., 2018; Pisegna et al., 2021; Schmalenberger et al., 2022), quality of life (Bauer et al., 2018; Breedveld-Peters et al., 2018; Demark-Wahnefried et al., 2006; Gopalakrishna et al., 2018; Inoue-Choi et al., 2013a; Kenkhuis et al., 2021; Krok-Schoen et al., 2021; Mosher et al., 2013; Pisegna et al., 2021; van Veen et al., 2019), health and lifestyle factors, including body mass index (Bennett et al., 2024; Breedveld-Peters et al., 2018; Demark-Wahnefried et al., 2006; Demark-Wahnefried et al., 2012; Inoue-Choi et al., 2013a; Klassen et al., 2018; Krok-Schoen et al., 2021; Mosher et al., 2013; Pelser et al., 2014; Pisegna et al., 2021; Schmalenberger et al., 2022; Snyder et al., 2009; Song et al., 2021; van Veen et al., 2019; van Zutphen et al., 2019) and physical functioning (Breedveld-Peters et al., 2018; Demark-Wahnefried et al., 2006; Demark-Wahnefried et al., 2003; Demark-Wahnefried et al., 2012; Inoue-Choi et al., 2014; Inoue-Choi et al., 2013a; Kenkhuis et al., 2021; Krok-Schoen et al., 2021; Schmalenberger et al., 2022; Snyder et al., 2009; van Veen et al., 2019; van Zutphen et al., 2019; Wang et al., 2021).

### 3.6. DQIs and their reported associations with health outcomes

A total of 15 (Bauer et al., 2018; Breedveld-Peters et al., 2018; Gopalakrishna et al., 2018; Inoue-Choi et al., 2013a; Kenkhuis et al., 2021; Krok-Schoen et al., 2021; McCullough et al., 2016; Mosher et al., 2009; Pelser et al., 2014; Pisegna et al., 2021; Schmalenberger et al., 2022; Song et al., 2021; Sun et al., 2018; van Veen et al., 2019; Wang et al., 2020) of the 28 included articles reported health outcomes in relation to DQI scores. The associations and correlations between diet quality and health outcomes are summarized in Table 2.

The most measured health outcomes were health-related quality of life (Bauer et al., 2018; Breedveld-Peters et al., 2018; Gopalakrishna et al., 2018; Inoue-Choi et al., 2013a; Kenkhuis et al., 2021; Krok-Schoen et al., 2021; Mosher et al., 2009; van Veen et al., 2019) (measured by an assessment tool, e.g., quality of life (EORTC assessment tool such as the European Organization for the Research and Treatment of Cancer Quality of Life Questionnaire (EORTC-C-Medical Outcomes Survey Short Form-36)30), Medical Outcomes Survey Short Form-36), and mortality (Inoue-Choi et al., 2013a; McCullough et al., 2016; Pelser et al., 2014; Song et al., 2021; Sun et al., 2018). Other health outcomes compared with the overall diet quality, or a dietary component/food group were skeletal muscle mass (Wang et al., 2021), self-rated health (Schmalenberger et al., 2022), serum albumin (Schmalenberger et al., 2022), disability, fatigue (Kenkhuis et al., 2021; van Veen et al., 2019) (other than within a QoL scale as above), and neuropathy (Kenkhuis et al., 2021).

Overall, a higher diet quality appeared to be associated with increased HR-QoL in breast, prostate, and colorectal cancer survivors, apart from one study that found no statistical significance (van Veen et al., 2019). The associations between mortality and diet quality were also inconsistent, depending on the type of cancer and the mortality type, that is, cancer-specific or other causes.

## 4. Discussion

Despite the growing population of cancer survivors, our scoping review revealed a significant gap in diet quality indices specifically designed for older cancer survivors. The WCRF/ACIR-2007–2018 and HEI were the most used DQI to assess diet quality in older cancer survivors, with only three DQIs specific to the older oncology population. While there were 40 unique adequacy and moderation components across the 12 DQIs, fruits and vegetables were ubiquitous, as separate or combined components. Whole grains, SFA and salt/sodium were also frequently incorporated among those indices. A higher diet quality appeared to be associated with a better physical HR-QoL in breast, prostate, and colorectal cancer survivors in all but one study, and a reduced cancer-specific mortality in colorectal cancer survivors.

The Healthy Eating Index (HEI) evaluates diet adherence to the Dietary Guidelines for Americans (DGA) and has been instrumental in examining relationships between diet quality and health outcomes, including cardiovascular disease mortality and cancer survivorship, where cardiotoxicity is a concern (Kennedy et al., 1995; Krebs-Smith et al., 2018). Studies using HEI-2005(Demark-Wahnefried et al., 2012; Mosher et al., 2009; Pelser et al., 2014), HEI-2010(Gopalakrishna et al., 2018; Klassen et al., 2018; Sun et al., 2018; Wang et al., 2021), HEI-2015 (Krok-Schoen et al., 2021; Pisegna et al., 2021; Schmalenberger et al., 2022) and RHEI (Miller et al., 2008; Snyder et al., 2009) have primarily focused on the U.S. population, while adaptations like the BHEI-R align with Brazilian dietary guidelines (Mazzutti et al., 2021). HEI scores use an energy-adjusted density approach (Kennedy et al., 1995) on the basis that consuming excess energy can progressively lead to overweight and obesity (Romieu et al., 2017). Studies also suggested that obesity could exacerbate various aspects of cancer survivorship, such as cancer recurrence, susceptibility to certain secondary cancers and quality of life (Schmitz et al., 2013; Sung et al., 2020). The research on different cancer types grows in parallel with their prevalence rates. Hence, evidence for the rarer cancers is scarce in comparison.

Three DQIs were specific to the general cancer population (WCRF/AICR-2007–2018, ACS), for the adult population including those 65 years and above. The WCRF/AICR-2007, (Breedveld-Peters et al., 2018; Inoue-Choi et al., 2014; Inoue-Choi et al., 2013a; Inoue-Choi et al., 2013b; van Veen et al., 2019) WCRF/AICR 2018, (Bennett et al., 2024; Kenkhuis et al., 2021; Koole et al., 2020; Song et al., 2021; van Zutphen et al., 2019) were employed and adapted in 10 studies. The WCRF/AICR-2007–2018, developed from thorough systematic reviews for cancer prevention, assesses dietary adherence to its recommendations and explores associations with cancer-related and other health outcomes, (WCRF/ACIR, 2007; WCRF/ACIR, 2018) and is meant to be applicable globally across various populations and countries (WCRF/ACIR, 2007; WCRF/ACIR, 2018). The WCRF/AICR-2007–2018 places its emphasis on modifiable lifestyle behaviors, promoting cancer prevention, and overall health (WCRF/ACIR, 2007; WCRF/ACIR, 2018). The recommendations include weight management, physical activity, and dietary guidelines for fruits, vegetables, and whole grains, as well as limitations on consuming ‘fast foods’ and other processed foods high in fat, starches, sugars, red and processed meat, sugar-sweetened drinks, and alcohol (WCRF/ACIR, 2007; WCRF/ACIR, 2018). While specific guidelines for cancer survivors are still limited, following the WCRF/AICR general Cancer Prevention Recommendations is advised. At present, studies that utilized the WCRF/AICR 2007–2018 were based on populations from the Netherlands (Breedveld-Peters et al., 2018; Kenkhuis et al., 2021; Koole et al., 2020; van Veen et al., 2019; van Zutphen et al., 2019), the USA (Inoue-Choi et al., 2014; Inoue-Choi et al., 2013a; Inoue-Choi et al., 2013b; Song et al., 2021) and Ireland (Bennett et al., 2024).The authors stated that the WCRF/AICR was selected based on evidence-based lifestyle recommendations, highlighting the importance of adherence to improve the health of cancer survivors.

The ACS guidelines diet score (McCullough et al., 2016), similar to the WCRF/ACIR 2007–2018, emphasizes a plant-based diet rich in fruits, vegetables, and whole grains while limiting red and processed meats. While these recommendations align with cancer prevention goals (Kushi et al., 2006), adherence can be challenging for older s due to physical limitations, transportation issues, and the effort required to prepare fresh produce (Han et al., 2024). Additionally, affordability of meat alternatives and cultural beliefs associating meat with strength may further discourage plant-based dietary choices (Drolet-Labelle et al., 2023).

Ultra-processed foods (UPFs), high in sugar, salt, saturated fat, refined grains and energy, are linked to chronic conditions, including cancer (Cordova et al., 2023). The NOVA classification defines UPFs as industrial products made largely of extracted or synthetic food components with additives for enhanced palatability (Monteiro et al., 2019). The WCRF/AICR-2007 (Breedveld-Peters et al., 2018; Inoue-Choi et al., 2014; Inoue-Choi et al., 2013a; Inoue-Choi et al., 2013b; van Veen et al., 2019), WCRF/AICR-2018 (Bennett et al., 2024; Kenkhuis et al., 2021; Koole et al., 2020; Song et al., 2021; van Zutphen et al., 2019) and DHD-index (Breedveld-Peters et al., 2018) incorporate sugary drinks and soft drinks as a moderation component. Added sugars are commonly found in sugar-sweetened beverages and energy-dense foods, such as fast foods or UPFs, and are associated with an increased risk of weight gain and being overweight or obese (Livingstone et al., 2022; Malik and Hu, 2022). However, the HEI did not include an UPF component within their indices (Guenther et al., 2013; Guenther et al., 2008; Krebs-Smith et al., 2018). Considering the growing evidence of increased UPF intake globally and their negative health impacts the WCRF/AICR recommended limiting the consumption of fast foods and other ultra processed foods high in fat, starches, or sugar (WCRF/ACIR, 2007; WCRF/ACIR, 2018). Interestingly, 4 out of 10 studies where WCRF/AICR was used did not operationalize this recommendation, (Breedveld-Peters et al., 2018; Inoue-Choi et al., 2014; Inoue-Choi et al., 2013a; Inoue-Choi et al., 2013b), the lack of international agreement on the definitions being one of the reasons stated (Breedveld-Peters et al., 2018). In contrast, Kenkhuis et al. (2021) operationalized the fast-food component based on ultra-processed foods according to the NOVA classification.

Most dietary guidelines caution against excessive consumption of red meat, mainly processed meat, for both health and environmental reasons (Rock et al., 2022). The ACS (McCullough et al., 2016) and the WCRF/AICR 2007–2018 (WCRF/ACIR, 2007; WCRF/ACIR, 2018) recommended that cancer survivors reduce red and processed meat intake, though the safe consumption threshold remains unclear. Given this uncertainty, the ACS suggests prioritizing protein sources like fish, poultry and beans over red meat (Rock et al., 2022).

Overall, higher diet quality was associated with better HR-QoL among breast, prostate, and colorectal cancer survivors, with the exception of one study that reported no statistically significant association (van Veen et al., 2019). The relationship between diet quality and mortality varied depending on the cancer type and whether the mortality was cancer-specific or due to other causes. The impact of diet quality on health outcomes may largely stem from the emphasis of DQIs on adequate fruit and vegetable intake. All DQIs incorporate fruits and vegetables, either as individual components or in combination. These foods offer several potential benefits for cancer survivors due to their rich content of micronutrients and phytochemicals (e.g., carotenoids, polyphenols, and sulfur compounds), as well as dietary fiber (Hurtado-Barroso et al., 2020). For instance, postmenopausal women with higher vegetable intake, reflected in elevated scores on the vegetable component of the AHEI, may have a lower risk of developing estrogen receptor-positive breast cancer (Fung et al., 2006). Additionally, adequate vegetable consumption has been associated with reduced overall cancer mortality among cancer survivors (Hurtado-Barroso et al., 2020). Notably, the strongest protective associations against cancer were observed for green-yellow and cruciferous vegetables, likely due to the chemo preventive properties of carotenoids and isothiocyanates (Hurtado-Barroso et al., 2020). Research on diet quality and health outcomes in older cancer survivors remains scarce as compared to the extensive body of research on other conditions such type 2 diabetes (Brlek and Gregorič, 2023), and in other population groups (Han et al., 2015).

One of the study’s strengths was that it followed the Preferred Reporting Items for Systematic Reviews and Meta-Analyses Extension for Scoping Reviews (PRISMA-ScR) (Tricco et al., 2018). Most of the DQIs reviewed in this study were developed based on dietary guidelines and recommendations. The authors acknowledge that excluding publications in languages other than English is a limitation of this review, particularly concerning DQIs that might have been developed in non-English-speaking low-income and middle-income countries. Research has shown that people from lower socio-economic status (SES) backgrounds consume less fruits, vegetables, whole grains, and fish, as compared to those from higher SES backgrounds (Gómez et al., 2021). Therefore, future research could focus on diet quality in these populations to address socio-economic disparities in dietary quality to improve health outcomes among geriatric oncology populations globally. The studies included in this review were also largely focused on breast, prostate, and colorectal cancer survivors, with limited data on survivors of other cancer types. Another important consideration was that the reviewed studies employed varying dietary assessment methods, such as FFQ and 24 h dietary recalls, which can influence the associations observed between diet and health outcomes due to variations in measurement error, recall bias, and participant burden (Shim, Oh and Kim, 2014). Most of the included studies used FFQs, which, despite being validated, may still overestimate intake due to reliance on memory and predefined food lists, potentially attenuating associations with health outcomes. The heterogeneity observed in certain health outcomes in this review may, in part, be attributed to the use of different dietary assessment methods. Overall, the associations between diet quality and health outcomes reported in this review should be interpreted with caution, as the studies are subjected to various biases, including those related to dietary assessment methods (as mentioned above) and sample selection.

## 5. Clinical and research implications

This review summarized the available validated dietary quality assessment tools and their individual dietary components, used to measure diet quality in older cancer survivors. Surprisingly, there is limited use of cancer- and older age-specific DQI in research and clinical literature. Many cancer survivors will require permanent alterations to their diet that may not be reflected in the general DQIs. Therefore, there is a need for cancer-specific DQIs that incorporate food groups that reflect the adequacy of nutritional intake in this intersectionality of older and cancer survivors. As older cancer survivors represent the largest growing age group among global cancer incidence, Clinicians will benefit from knowledge to recognize which components of the diet are key to a high-quality diet (Siegel et al., 2024). Researchers studying DQIs would benefit from studying their associations with functional health outcomes rather than HR-QOL or mortality, to be consistent with guidelines and trials in the geriatric oncology field (Mohile et al., 2018). These suggestions from our findings will help inform researchers and clinicians on validated measurements used to examine patient-reported dietary information and/or evaluate interventions that involve diet improvement and nutrition in this population.

## 6. Conclusion

This scoping review summarised published DQIs used to evaluate the diet quality of older cancer survivors and their development or adaptation. Most DQIs examined were derived from dietary guidelines and data from high-income countries, with only two DQIs explicitly developed for the cancer population. Overall, the DQIs found in this review were not explicitly designed for the older cancer survivors, and their unique differences in dietary requirements may not necessarily be reflected in DQIs designed for the general population. A higher diet quality appeared to be associated with a better physical HR-QoL in breast, prostate, and colorectal cancer survivors in all but one study and reduced all-cause mortality in rectal cancer survivors. Future research should consider adaptation of the dietary recommendations for this intersectional population and develop DQIs for this population to better capture how a high-quality diet can potentially enhance the health of older cancer survivors.

Table S1: Development, scoring and evaluation of DQIs used with older cancer survivors; Table S2: Summary of components used in the current DQIs for older cancer survivors; Table S3: Final article list exclusion DQIs in older cancer survivors; Table S4, Search Strategy; Table S5: PRISMA-ScR Checklist.

## Supplementary Material

S1-S5 tables

## Figures and Tables

**Table 1:** Mapping of dietary components to Dietary quality index.

	HEI-2005 (Demark-Wahnefried et al., 2012; Mosher et al., 2009; Pelser et al., 2014)	HEI-2010 (Gopalakrishna et al., 2018; Klassen et al., 2018; Sun et al., 2018; Wang et al., 2021)	HEI-2015 (Krok-Schoen et al., 2021; Pisegna et al., 2021; Schmalenberger et al., 2022)	RHEI (Miller et al., 2008; Snyder et al., 2009)	BHEI- R (Mazzutti et al., 2021)	DQI-R (Clutter Snyder et al., 2007; Demark-Wahnefried et al., 2006; Demark-Wahnefried et al., 2003)	MDS (Bauer et al., 2018)	DHD-I (Breedveld-Peters et al., 2018)	9-item index (Klassen et al., 2018)	WCRF/AICR-2007 (Breedveld-Peters et al., 2018; Inoue-Choi et al., 2014; Inoue-Choi et al., 2013a; Inoue-Choi et al., 2013b; van Veen et al., 2019)	WCRF/AICR-2018 (Bennett et al., 2024; Kenkhuis et al., 2021; Koole et al., 2020; Song et al., 2021; van Zutphen et al., 2019)	ACS (McCullough et al., 2016)
Adequacy (*n* = 23)^1^												
Total Grains	✓			✓	✓		✓					✓
Whole grains	✓	✓	✓	✓	✓	✓						
Total Vegetables	✓	✓	✓	✓	✓	✓	✓	✓				
Dark green leafy, Orange vegetables, Legumes	✓			✓	✓							
Greens and Beans		✓	✓									
Total Fruit	✓	✓	✓	✓	✓	✓	✓	✓				
Whole fruit	✓	✓	✓	✓	✓							
Fruit and Vegetables combined									✓	✓	✓	✓
Beans, pulses, legumes							✓					
Nuts							✓					
Dairy, Milk	✓	✓	✓	✓	✓							
Total protein foods		✓	✓									
Fish							✓	✓				
Seafood and Plant Protein		✓	✓									
Meats and Beans	✓			✓								
Meat, Eggs and Beans					✓							
Fiber								✓	✓	✓	✓	
Calcium						✓			✓			
Iron						✓						
Vitamin D									✓			
(PUFA+MUFA)/SFA		✓	✓									
Ratio of PUFA: SFA							✓					
Oils-MUFA, PUFA, oilseeds, fish fat	✓				✓							
Moderation (*n* = 17)^1^												
Red/processed										✓	✓	✓
meat												
Refined Grains		✓	✓									
Added sugar/sugary foods and drinks			✓					✓				
Sugar-sweetened beverages and fruit drinks								✓		✓	✓	
Percent of total calories from ultra-processed foods (aUPFs)											✓	
Total fat						✓						
Saturated Fat	✓		✓	✓	✓	✓	✓	✓	✓			
Trans fat								✓				
Cholesterol						✓			✓			
Salt or Sodium	✓	✓	✓	✓	✓			✓	✓	✓		
Alcohol							✓	✓	✓	✓	✓	
Calories from Solid Fats, Alcoholic Beverages, Added Sugars (SoFAAS)	✓	✓		✓	✓							
Total Calories									✓			
Diet Diversity					✓							
Dietary moderation					✓							
Dietary Supplements										✓		
Energy density – *considered when scoring other components* e. g.*, per 1000kcal*	✓	✓	✓	✓	✓	✓				✓		

HEI-2005: Healthy Eating Index 2005, HEI-2010: Healthy Eating Index 2015, HEI-2015: Healthy Eating Index 2015, BHEI-R: Brazil Healthy Eating Index Revised, RHEI: Revised Healthy Eating Index, WCRF/AICR-2007: World Cancer Research Fund/American Institute for Cancer Research 2007, WCRF/AICR-2018: World Cancer Research Fund/American Institute for Cancer Research 2018, DQI-R: Diet Quality Index Revised, Mediterranean Diet Score: MDS, DHD-I: Dutch Healthy Diet Index, ACS: The American Cancer Society guidelines diet scores, PUFA: Polyunsaturated fatty acids, MUFA: Monounsaturated fatty acids

1 The adequacy components focus on foods that provide essential nutrients and promote overall health, supporting bodily functions and disease prevention.

2 The moderation components highlight foods that should be limited or consumed sparingly, as excessive intake may lead to negative health outcomes such as obesity, heart disease, and other chronic conditions.

**Table 2:** Health outcomes associated with diet scores from diet quality indices in older cancer survivors.

Outcome	Key summary of associated health-related outcomes and cancer types	
HR-QoL – Global	In bladder cancer survivors, no statistically significantly difference between those with higher HEI-2010 compared to lower HEI-2010 adherence in global HR-QoL (Gopalakrishna et al., 2018).	⨯
	In female colorectal cancer survivors, higher DHD-I score is associated with higher global QoL (ß=5.1, 95 % CI: 0.1, 10.1, *P* < 0.05) in female colorectal cancer survivors (Breedveld-Peters et al., 2018).	✓
	In colorectal cancer survivors, dietary adherence to the WCRF/AICR-2007 dietary components only were not significantly associated with global health (P>0.05) (van Veen et al., 2019).	⨯
	In colorectal cancer survivors, increased vegetable intake (per 50g) using WCRF/AICR-2018 was linked to enhanced global quality of life (ß=2.6, 95 %CI: 0.6, 4.7, *P* < 0.05) (Kenkhuis et al., 2021).	✓
HR-QoL - Physical	In breast, prostate, colorectal cancer survivors, positive correlations between HEI-2005 scores and physical quality of life (adjusted r^2^=0.1, P=0.005) and physical functioning (r^2^=0.01, P=0.01) (Mosher et al., 2009).	✓
	In female breast, hematologic and gynecologic cancer survivors, positive correlation between HEI-2015 scores and physical functioning (r=0.37, *P* < 0.001 (Krok-Schoen et al., 2021));	✓
	Total significant associations between HEI-2015 scores and physical functioning (F (1, 110) =18.55, *P* < 0.001; Adjusted r^2^=0.14, SE=9.38) based on multiple linear regression (Krok-Schoen et al., 2021).	
	Individuals who had higher HEI scores were more likely to report higher physical functioning (ß=0.38, *P* < 0.001) (Krok-Schoen et al., 2021).	
	In female colorectal cancer survivors, higher DHD-I score is associated with higher physical functioning scores (ß=8.3, 95 % CI: 3.2, 13.4, *P* < 0.05) (Breedveld-Peters et al., 2018).	✓
	In overweight-obese individuals colorectal cancer survivors (BMI≥25kg/m^2^), higher DHD-I score is associated with higher Physical functioning scores (ß=5.4, 95 % CI: 1.5, 9.3, *P* < 0.05) (Breedveld-Peters et al., 2018).	
	In older female cancer survivors, adherence to the recommendation for less alcohol intake as per WCRF/AICR-2007 was not associated with physical component summary scores (Inoue-Choi et al., 2013a).	⨯
	In colorectal cancer survivors, elevated intake of fruits and vegetables (per 100 g) was associated with improved physical functioning (ß=3.2, 95 % CI: 0.8, 5.5, *P* < 0.05).	✓
	Higher consumption of energy-dense foods (per 100 kJ/100 g) as per the WCRF/AICR-2018 was associated with poorer physical functioning (ß=−4.2, 95 % CI: −7.1, −1.25.5, *P* < 0.05) (Kenkhuis et al., 2021).	
	In prostate cancer survivors, No significant association between the Mediterranean Diet Score after diagnosis and sexual function	✓
	Only in individuals with a history of benign prostatic hyperplasia or lower urinary tract symptoms before their prostate cancer diagnosis, higher post-diagnosis vegetable intake was associated with modestly higher urinary incontinence scores i.e., better urinary function (P-trend=0.003), after adjusting for pre-diagnosis diet. Lower post-diagnostic polyunsaturated fat intake was associated with higher urinary irritation/obstruction scores i.e., better urinary function comparing lowest to highest quintile (P-trend = 0.005) (Bauer et al., 2018).	
Fatigue	In colorectal cancer survivors, dietary adherence to the WCRF/AICR-2007 dietary components only was not significantly associated with fatigue (P>0.05) (van Veen et al., 2019).	⨯
	In colorectal, Increased vegetable intake (per 50 g) (WCRF/AICR-2018) was linked to reduced levels of fatigue (ß=−4.5, 95 %CI: −7.6, −1.4, *P* < 0.05) (Kenkhuis et al., 2021).	✓
Pain	In breast, prostate, colorectal cancer survivors, positive correlations between HEI-2005 scores and pain (adjusted r^2^=0.08, p=0.048) (Mosher et al., 2009).	✓
Mental	In older female cancer survivors, higher HEI-2015 scores (β = 0.30, p = 0.011) was associated with higher mental HR QoL (Model: F = 11.50, p < 0.001) (Pisegna et al., 2021).	✓
	In older female cancer survivors, who met more dietary recommendations (within the WCRF/AICR-2007) had higher MCS (P-trend=0.03). MCS was higher only with adherence to the recommendations for reduced red meat (P=0.006) and sodium (P=0.0003) intake. Adherence to the recommendation for less alcohol intake was not associated with MCS (Inoue-Choi et al., 2013a).	✓
Mortality	In older female survivors, overall supplement and individual multivitamin use (WCRF/AICR-2007) were not linked to mortality (P>0.05 for all) (Inoue-Choi et al., 2013a).	⨯
	Mortality (overall): Post-diagnostic WCRF/AICR-2018 dietary components only score did not show a statistically significant association with overall mortality (Song et al., 2021).	⨯
	In female breast cancer survivors, Mortality Pre-diagnostic ACS diet scores was not associated with mortality from any causes. No significant association was observed between fruit and vegetable consumption (both pre- and post-diagnosis) and all-causes of mortality (McCullough et al., 2016).	⨯
	In female breast cancer survivors, post-diagnostic ACS diet score was associated with a borderline lower risk of other causes of death, e.g., other cancers, respiratory illness, and Alzheimer’s disease (RR 0.78, 95 % CI 0.56–1.07, P-trend = 0.03; per two-point increase in score (RR 0.88, 95 %CI 0.79–0.99). Higher consumption of red and processed meat after diagnosis was the only component of the ACS diet score independently associated with non-breast cancer causes of mortality. Specifically, the lowest versus highest quartile of red and processed meat intake after diagnosis was associated with a statistically significant 48 %, 43 %, and 36 % lower risk of CVD, non-breast, non-CVD causes of death, and total mortality, respectively (McCullough et al., 2016).	✓
	In colorectal cancer survivors, individuals in the highest quintile of HEI-2005 scores had reduced all-cause mortality (RR: 0.60; 95 %CI: 0.42–0.86, P=0.04) compared to those in the lowest (Pelser et al., 2014).	✓
	In colorectal cancer survivors, post-diagnostic WCRF/AICR-2018 dietary components only score did not show a statistically significant association with colorectal cancer–specific mortality(Song et al., 2021).	⨯
	In breast cancer survivors, women who experienced a decrease in diet quality (≥15 % decrease in HEI-2010 score over a span of 12 years) had a significantly higher risk of death from breast cancer compared to those who maintained stable diet quality (adjusted HR 1.66, 95 % CI 1.09 to 2.52). However, increasing diet quality did not show significant associations with the risk of death from any causes, breast cancer or other causes, after adjusting for changes in Body mass index over time. Pre-diagnosis diet quality was not associated with death from all causes, breast cancer, or other causes, (P>0.05 for all). Higher post-diagnosis diet quality (Quartile 4 vs Quartile 1) was associated with lower risk of death from causes other than breast cancer (adjusted HR 0.72, 95 % CI 0.55–0.94) (Sun et al., 2018).	✓
	In female breast cancer survivors, post-diagnosis ACS diet scores was not associated with breast cancer-specific mortality (RR: 1.44, 95 % CI 0.90–2.30 for scores 6–9 vs 0–2) (McCullough et al., 2016).	⨯
	In female breast cancer survivors, the lowest versus highest quartile of red and processed meat intake, as per ACS score, after diagnosis was associated with a statistically significant 48 % lower risk of CVD mortality (McCullough et al., 2016).	⨯
Muscle Mass	In bladder cancer survivors, HEI-2010 scores not associated with skeletal muscle mass (P=0.822) (Wang et al., 2021).	⨯
Self-rated Health	In female breast, hematologic and gynecologic cancer survivors, no significant associations between HEI-2015 and self-rated healthy in adjusted regression models (Schmalenberger et al., 2022).	⨯
Serum Albumin	In female breast, hematologic and gynecologic cancer survivors, no significant associations between HEI-2015 and albumin in adjusted regression models (Schmalenberger et al., 2022).	⨯
Disability	In female colorectal cancer survivors, higher DHD-I score is associated with lower disability scores in female (ß=−5.6, 95 % CI: −9.5, −1.6, *P* < 0.05) (Breedveld-Peters et al., 2018).	✓
Neuropathy	In colorectal cancers survivors, no discernible associations were observed between adherence to dietary recommendations by WCRF/AICR-2018 and neuropathy (Kenkhuis et al., 2021).	⨯

HR-QoL, Health-related Quality of Life; HEI, Healthy Eating Index; DHD-I, Dutch Healthy Diet Index WCRF/AICR, World Cancer Research Fund/American Institution of Cancer Research diet only components scores; MDS, Mediterranean Diet Score; ACS, American Cancer Society guidelines diet component only scores ✔=association found; ⨯=association not found

## HIGHLIGHTS

- Total vegetables, total fruits, whole grains, saturated fat, and salt/sodium were the most frequently incorporated components within a diet quality index for older cancer survivors.
- Higher diet quality was associated with higher health-related quality of life in breast, prostate, and colorectal cancer survivors in all but one study.
- The associations between mortality and diet quality were inconsistent, depending on the type of cancer and the mortality type i.e., cancer-specific or other causes.

## References

[R1] Bauer SR, Van Blarigan EL, Stampfer MJ, Chan JM, & Kenfield SA (2018). Mediterranean diet after prostate cancer diagnosis and urinary and sexual functioning: The health professionals follow-up study. The Prostate, 78, 202–212. doi: 10.1002/pros.23457 29194691 PMC5768457

[R2] Bennett AE, O’Neill L, Doyle SL, Guinan EM, O’Sullivan J, Reynolds JV, & Hussey J (2024). Nutrient intakes and gastrointestinal symptoms among esophagogastric cancer survivors up to 5 years post-surgery. Nutrition and Cancer, 76, 442–451. doi: 10.1080/01635581.2024.2328380 38486410

[R3] Breedveld-Peters JJL, Koole JL, Müller-Schulte E, van der Linden BWA, Windhausen C, Bours MJL, van Roekel EH, & Weijenberg MP (2018). Colorectal cancers survivors’ adherence to lifestyle recommendations and cross-sectional associations with health-related quality of life. British Journal of Nutrition, 120, 188–197. doi: 10.1017/s0007114518000661 29658446

[R4] Brlek A, & Gregorič M (2023). Diet quality indices and their associations with all-cause mortality, CVD and type 2 diabetes mellitus: An umbrella review. British Journal of Nutrition, 130, 709–718. doi: 10.1017/s0007114522003701 36423897

[R5] Clutter Snyder DMSRD, Sloane RMPH, Haines PSDRD, Miller P, Clipp ECPRN, Morey MCP, Pieper CD, Cohen HMD, & Demark-Wahnefried WPRD (2007). The diet quality index-revised: A tool to promote and evaluate dietary change among older cancer survivors enrolled in a home-based intervention trial. Journal of the American Dietetic Association, 107, 1519–1529. doi: 10.1016/j.jada.2005.05.035 17761229

[R6] Cordova R, Viallon V, Fontvieille E, Peruchet-Noray L, Jansana A, Wagner K-H, Kyrø C, Tjønneland A, Katzke V, Bajracharya R, Schulze MB, Masala G, Sieri S, Panico S, Ricceri F, Tumino R, Boer JMA, Verschuren WMM, van der Schouw YT, Jakszyn P, Redondo-Sánchez D, Amiano P, Huerta JM, Guevara M, Borné Y, Sonestedt E, Tsilidis KK, Millett C, Heath AK, Aglago EK, Aune D, Gunter MJ, Ferrari P, Huybrechts I, & Freisling H (2023). Consumption of ultra-processed foods and risk of multimorbidity of cancer and cardiometabolic diseases: A multinational cohort study. The Lancet regional health Europe, 35. doi: 10.1016/j.lanepe.2023.100771, 100771–100771. 38115963 PMC10730313

[R7] Demark-Wahnefried W, Clipp EC, Morey MC, Pieper CF, Sloane R, Snyder DC, & Cohen HJ (2006). Lifestyle intervention development study to improve physical function in older adults with cancer: Outcomes from Project LEAD. Journal of Clinical Oncology, 24, 3465–3473. doi: 10.1200/jco.2006.05.7224 16849763 PMC1532928

[R8] Demark-Wahnefried W, Morey MC, Clipp EC, Pieper CF, Snyder DC, Sloane R, & Cohen HJ (2003). Leading the way in exercise and diet (Project LEAD): intervening to improve function among older breast and prostate cancer survivors. Controlled Clinical Trials, 24, 206–223. doi: 10.1200/jco.2005.23.16_suppl.8138 12689742

[R9] Demark-Wahnefried W, Morey MC, Sloane R, Snyder DC, Miller PE, Hartman TJ, & Cohen HJ (2012). Reach out to enhance wellness home-based diet-exercise intervention promotes reproducible and sustainable long-term improvements in health behaviors, body weight, and physical functioning in older, overweight/obese cancer survivors. Journal of Clinical Oncology, 30, 2354–2361. doi: 10.1200/jco.2011.40.0895 22614994 PMC3675693

[R10] Demark-Wahnefried W, Peterson B, McBride C, Lipkus I, & Clipp E (2000). Current health behaviors and readiness to pursue life-style changes among men and women diagnosed with early stage prostate and breast carcinomas. Cancer, 88, 674–684. doi: 10.1002/(sici)1097-0142(20000201)88:3<674::aid-cncr26>3.3.co;2-i 10649263

[R11] Drolet-Labelle V, Laurin D, Bédard A, Drapeau V, & Desroches S (2023). Beliefs underlying older adults’ intention to consume plant-based protein foods: A qualitative study. Appetite, 180, Article 106346. doi: 10.1016/j.appet.2022.106346 36257358

[R12] Fung TT, Hu FB, McCullough ML, Newby PK, Willett WC, & Holmes MD (2006). Diet quality is associated with the risk of estrogen receptor-negative breast cancer in postmenopausal women. The Journal of Nutrition, 136(2), 466–472. doi: 10.1093/jn/136.2.466 16424129

[R13] Hurtado-Barroso S, Trius-Soler M, Lamuela-Raventós RM, & Zamora-Ros R (2020). Vegetable and fruit consumption and prognosis among cancer survivors: A systematic review and meta-analysis of cohort studies. Advances in Nutrition, 11(6), 1569–1582. doi: 10.1093/advances/nmaa082 32717747 PMC7666913

[R14] Gómez G, Kovalskys I, Leme ACB, Quesada D, Rigotti A, Cortes Sanabria LY, Yepez Garcia MC, Liria-Domínguez MR, Herrera-Cuenca M, Fisberg RM, & Nogueira Previdelli A (2021). Socioeconomic status impact on diet quality and body mass index in eight Latin American countries: ELANS study results. Nutrients, 13(7). doi: 10.3390/nu13072404. p.2404. 34371915 PMC8308629

[R15] Gopalakrishna A, Chang A, Longo TA, Fantony JJ, Harrison MR, Wischmeyer PE, & Inman BA (2018). Dietary patterns and health-related quality of life in bladder cancer survivors. Urologic oncology, 36, 469. doi: 10.1016/j.urolonc.2018.06.001. e421–469.e429. 30126776

[R16] Guenther PM, Casavale KO, Reedy J, Kirkpatrick SI, Hiza HA, Kuczynski KJ, Kahle LL, & Krebs-Smith SM (2013). Update of the Healthy Eating Index: HEI-2010. *Journal of the Academy of Nutrition and Dietetics*, 113, 569–580. doi: 10.1016/j.jand.2012.12.016 23415502 PMC3810369

[R17] Guenther PM, Reedy J, & Krebs-Smith SM (2008). Development of the Healthy Eating Index-2005. Journal of the American Dietetic Association, 108, 1896–1901. doi: 10.3389/fnut.2022.952223 18954580

[R18] Han CY, Colega M, Quah EPL, Chan YH, Godfrey KM, Kwek K, Saw S-M, Gluckman PD, Chong Y-S, & Chong MF-F (2015). A healthy eating index to measure diet quality in pregnant women in Singapore: A cross-sectional study. BMC Nutrition, 1, 39. doi: 10.1186/s40795-015-0029-3

[R19] Han CY, Middleton G, Doh J, Yaxley A, Sharma Y, Baldwin C, & Miller M (2024). Barriers and enablers to a hospital-to-home, combined exercise and nutrition, self-managed program for pre-frail and frail hospitalised older adults. InHealthcare, 12(6), 678. doi: 10.3390/healthcare12060678 PMC10970091 38540641

[R20] Inoue-Choi M, Greenlee H, Oppeneer SJ, & Robien K (2014). The Association between Postdiagnosis Dietary Supplement Use and Total Mortality differs by diet quality among older female cancer survivors. Cancer Epidemiology, Biomarkers & Prevention, 23, 865–875. doi: 10.1158/1055-9965.epi-13-1303 PMC4066370 24621441

[R21] Inoue-Choi M, Lazovich D, Prizment AE, & Robien K (2013a). Adherence to the world cancer research fund/american institute for cancer research recommendations for cancer prevention is associated with better health-related quality of life among elderly female Cancer survivors. Journal of Clinical Oncology, 31, 1758–1766. doi: 10.1200/jco.2012.45.4462 23569318 PMC3641697

[R22] Inoue-Choi M, Robien K, & Lazovich D (2013b). Adherence to the WCRF/AICR guidelines for Cancer prevention is associated with lower mortality among older female cancer survivors. Cancer Epidemiology, Biomarkers & Prevention, 22, 792–802. doi: 10.1158/1055-9965.epi-13-0054 PMC3650116 23462914

[R23] Izano MA, Fung TT, Chiuve SS, Hu FB, & Holmes MD (2013). Are diet quality scores after breast cancer diagnosis associated with improved breast cancer survival? Nutrition and Cancer, 65, 820–826. doi: 10.1080/01635581.2013.804939 23909725 PMC3776311

[R24] Kenkhuis M-F, van der Linden BWA, Breedveld-Peters JJL, Koole JL, van Roekel EH, Breukink SO, Mols F, Weijenberg MP, & Bours MJL (2021). Associations of the dietary World Cancer Research Fund/American Institute for Cancer Research (WCRF/AICR) recommendations with patient-reported outcomes in colorectal cancer survivors 2–10 years post-diagnosis: A cross-sectional analysis. British Journal of Nutrition, 125, 1188–1200. doi: 10.1017/s0007114520003487 33087189

[R25] Kennedy ET, Ohls J, Carlson S, & Fleming K (1995). The Healthy Eating Index: Design and applications. Journal of the American Dietetic Association, 95, 1103–1108. doi: 10.1016/s0002-8223(95)00300-2 7560680

[R26] Klassen AC, Smith KC, Shuster M, Coa KI, Caulfield LE, Helzlsouer KJ, Peairs KS, Shockney LD, Stoney D, & Hannum S (2018). “We’re just not prepared for eating over our whole life”: A mixed methods approach to understanding dietary behaviors among longer term cancer survivors. Integrative Cancer Therapies, 17, 350–362. doi: 10.1177/1534735417731515 28971702 PMC6041917

[R27] Kleckner AS, & Magnuson A (2022). The nutritional needs of older cancer survivors. Journal of Geriatric Oncology, 13, 738–741. doi: 10.1016/j.jgo.2021.12.007 34906443 PMC9187777

[R28] Koole JL, Bours MJL, Breedveld-Peters JJL, van Roekel EH, van Dongen MCJM, Eussen SJPM, van Zutphen M, van Duijnhoven FJB, Boshuizen HC, & Weijenberg MP (2020). Evaluating the validity of a food frequency questionnaire in comparison with a 7-day dietary record for measuring dietary intake in a population of survivors of colorectal cancer. Journal of the Academy of Nutrition and Dietetics, 120, 245–257. doi: 10.1016/j.jand.2019.09.008 31806573

[R29] Kranz S, Hasan F, Kennedy E, Zoellner J, Guertin KA, Shivappa N, Hébert JR, Anderson R, & Cohn W (2022). Diet quality and dietary inflammatory index score among women’s cancer survivors. International Journal of Environmental Research and Public health, 19. doi: 10.3390/ijerph19041916, 1916. 35206105 PMC8871885

[R30] Krebs-Smith SM, Pannucci TE, Subar AF, Kirkpatrick SI, Lerman JL, Tooze JA, Wilson MM, & Reedy J (2018). Update of the healthy eating index: HEI-2015. *Journal of the Academy of Nutrition and Dietetics*, 118, 1591–1602. doi: 10.1016/j.jand.2018.05.021 30146071 PMC6719291

[R31] Krok-Schoen JL, Pisegna J, Arthur E, Ridgway E, Stephens C, & Rosko AE (2021). Prevalence of lifestyle behaviors and associations with health-related quality of life among older female cancer survivors. Supportive Care in Cancer, 29, 3049–3059. doi: 10.1007/s00520-020-05812-3 33040283

[R32] Kushi LH, Doyle C, McCullough M, Rock CL, Demark-Wahnefried W, Bandera EV, Gapstur S, Patel AV, Andrews K, & Gansler T (2006). American Cancer Society guidelines on nutrition and physical activity for cancer prevention: Reducing the risk of cancer with healthy food choices and physical activity. *CA*: A Cancer Journal for Clinicians, 62, 30–67. doi: 10.3322/canjclin.56.5.254 22237782

[R33] Livingstone KM, Sexton-Dhamu MJ, Pendergast FJ, Worsley A, Brayner B, & McNaughton SA (2022). Energy-dense dietary patterns high in free sugars and saturated fat and associations with obesity in young adults. European Journal of Nutrition, 61, 1595–1607. doi: 10.1007/s00394-021-02758-y 34870745 PMC8921009

[R34] Long T, Zhang K, Chen Y, & Wu C (2022). Trends in diet quality among older US adults from 2001 to 2018. JAMA Network Open, 5. doi: 10.1001/jamanetworkopen.2022.1880. e221880–e221880. 35275167 PMC8917422

[R35] Malik VS, & Hu FB (2022). The role of sugar-sweetened beverages in the global epidemics of obesity and chronic diseases. Nature Reviews Endocrinology, 18, 205–218. doi: 10.1038/s41574-021-00627-6 PMC8778490 35064240

[R36] Mazzutti FS, Custodio IDD, Lima MTM, de Carvalho KP, Pereira TSS, Molina M.d. C. B., Canto PPL, Paiva CE, & Maia Y. C.d. P. (2021). Breast cancer survivors undergoing endocrine therapy have a worrying risk factor profile for cardiovascular diseases. Nutrients, 13, 1114. doi: 10.3390/nu13041114 33805280 PMC8067236

[R37] McCullough ML, Gapstur SM, Shah R, Campbell PT, Wang Y, Doyle C, & Gaudet MM (2016). Pre-and postdiagnostic diet in relation to mortality among breast cancer survivors in the CPS-II Nutrition Cohort. Cancer Causes and Control, 27, 1303–1314. doi: 10.1007/s10552-016-0802-x 27644127

[R38] Miller P, Demark-Wahnefried W, Snyder DC, Sloane R, Morey MC, Cohen H, Kranz S, Mitchell DC, & Hartman TJ (2008). Dietary supplement use among elderly, long-term cancer survivors. Journal of Cancer Survivorship, 2, 138–148. doi: 10.1007/s11764-008-0060-3 18792788 PMC2766274

[R39] Mohile SG, Dale W, Somerfield MR, Schonberg MA, Boyd CM, Burhenn PS, Canin B, Cohen HJ, Holmes HM, Hopkins JO, Janelsins MC, Khorana AA, Klepin HD, Lichtman SM, Mustian KM, Tew WP, & Hurria A (2018). Practical assessment and management of vulnerabilities in older patients receiving chemotherapy: ASCO guideline for geriatric oncology. Journal of Clinical Oncology, 36, 2326–2347. doi: 10.1200/jop.18.00180 29782209 PMC6063790

[R40] Monteiro CA, Cannon G, Levy RB, Moubarac JC, Louzada ML, Rauber F, Khandpur N, Cediel G, Neri D, Martinez-Steele E, Baraldi LG, & Jaime PC (2019). Ultra-processed foods: What they are and how to identify them. Public Health Nutrition, 22, 936–941. doi: 10.1017/s1368980018003762 30744710 PMC10260459

[R41] Mosher CE, Lipkus I, Sloane R, Snyder DC, Lobach DF, & Demark-Wahnefried W (2013). Long-term outcomes of the FRESH START trial: Exploring the role of self-efficacy in cancer survivors’ maintenance of dietary practices and physical activity. Psycho-Oncology, 22, 876–885. doi: 10.1002/pon.3089 22544562 PMC3429767

[R42] Mosher CE, Sloane R, Morey MC, Snyder DC, Cohen HJ, Miller PE, & Demark-Wahnefried W (2009). Associations between lifestyle factors and quality of life among older long-term breast, prostate, and colorectal cancer survivors. Cancer, 115, 4001–4009. doi: 10.1002/cncr.24436 19637244 PMC2743037

[R43] Park S-Y, Kang M, Shvetsov YB, Setiawan VW, Boushey CJ, Haiman CA, Wilkens LR, & Le Marchand L (2022). Diet quality and all-cause and cancer-specific mortality in cancer survivors and non-cancer individuals: The Multiethnic Cohort Study. European Journal of Nutrition, 1–9. doi: 10.1007/s00394-021-02700-2 PMC8857026 34657186

[R44] Pelser C, Arem H, Pfeiffer RM, Elena JW, Alfano CM, Hollenbeck AR, & Park Y (2014). Prediagnostic lifestyle factors and survival after colon and rectal cancer diagnosis in the National Institutes of Health (NIH)-AARP Diet and Health Study. Cancer, 120, 1540–1547. doi: 10.1002/cncr.28573 24591061 PMC4151292

[R45] Pisegna J, Xu M, Spees C, & Krok-Schoen JL (2021). Mental health-related quality of life is associated with diet quality among survivors of breast cancer. Supportive Care in Cancer, 29, 2021–2028. doi: 10.1007/s00520-020-05698-1 32844314

[R46] Rock CL, Thomson CA, Sullivan KR, Howe CL, Kushi LH, Caan BJ, Neuhouser ML, Bandera EV, Wang Y, Robien K, Basen-Engquist KM, Brown JC, Courneya KS, Crane TE, Garcia DO, Grant BL, Hamilton KK, Hartman SJ, Kenfield SA, Martinez ME, Meyerhardt JA, Nekhlyudov L, Overholser L, Patel AV, Pinto BM, Platek ME, Rees-Punia E, Spees CK, Gapstur SM, & McCullough ML (2022). American Cancer Society nutrition and physical activity guideline for cancer survivors. *CA*: A Cancer Journal for Clinicians, 72, 230–262. doi: 10.3322/caac.21719 35294043

[R47] Romieu I, Dossus L, Barquera S, Blottière HM, Franks PW, Gunter M, Hwalla N, Hursting SD, Leitzmann M, Margetts B, Nishida C, Potischman N, Seidell J, Stepien M, Wang Y, Westerterp K, Winichagoon P, Wiseman M, & Willett WC (2017). Energy balance and obesity: What are the main drivers? Cancer Causes and Control, 28, 247–258. doi: 10.1007/s10552-017-0869-z 28210884 PMC5325830

[R48] Ryu SW, Son YG, & Lee MK (2020). Motivators and barriers to adoption of a healthy diet by survivors of stomach cancer: A cross-sectional study. European Journal of Oncology Nursing, 44. doi: 10.1016/j.ejon.2019.101703, 101703-101703. 31816509

[R49] Schmalenberger M, Spees C, Bittoni AM, & Krok-Schoen JL (2022). Association of dietary quality, inflammatory markers, and physical functioning among older female cancer survivors. Nutrition and Cancer, 74, 496–504. doi: 10.1080/01635581.2021.1892157 33678060

[R50] Schmitz KH, Neuhouser ML, Agurs-Collins T, Zanetti KA, Cadmus-Bertram L, Dean LT, & Drake BF (2013). Impact of obesity on cancer survivorship and the potential relevance of race and ethnicity. *JNCI*: Journal of the National Cancer Institute, 105, 1344–1354. doi: 10.1093/jnci/djt223 23990667 PMC3776266

[R51] Schwedhelm C, Boeing H, Hoffmann G, Aleksandrova K, & Schwingshackl L (2016). Effect of diet on mortality and cancer recurrence among cancer survivors: A systematic review and meta-analysis of cohort studies. Nutrition Reviews, 74, 737–748. doi: 10.1093/nutrit/nuw045 27864535 PMC5181206

[R52] Shim JS, Oh K, & Kim HC (2014). Dietary assessment methods in epidemiologic studies. Epidemiology and Health, 36. doi: 10.4178/epih/e2014009. e2014009. 25078382 PMC4154347

[R53] Siegel RL, Giaquinto AN, & Jemal A (2024). Cancer statistics, 2024. CA: A Cancer Journal for Clinicians, 74, 12–49. doi: 10.3322/caac.21820 38230766

[R54] Snyder DC, Morey MC, Sloane R, Stull V, Cohen HJ, Peterson B, Pieper C, Hartman TJ, Miller PE, Mitchell DC, & Demark-Wahnefried W (2009). Reach out to ENhancE Wellness in older cancer survivors (RENEW): design, methods and recruitment challenges of a home-based exercise and diet intervention to improve physical function among long-term survivors of breast, prostate, and colorectal cancer. Psycho-Oncology, 18, 429–439. doi: 10.1002/pon.1491 19117329 PMC2748788

[R55] Song R, Petimar J, Wang M, Tabung FK, Song M, Liu L, Lee DH, Giovannucci EL, Zhang X, & Smith-Warner SA (2021). Adherence to the world cancer research fund/american institute for cancer research cancer prevention recommendations and colorectal cancer survival. Cancer Epidemiology, Biomarkers & Prevention, 30, 1816–1825. doi: 10.1158/1055-9965.epi-21-0120 PMC8492523 34272268

[R56] Sulicka J, Pac A, Puzianowska-Kuźnicka M, Zdrojewski T, Chudek J, Tobiasz-Adamczyk B, Mossakowska M, Skalska A, Więcek A, & Grodzicki T (2018). Health status of older cancer survivors—Results of the PolSenior study. Journal of Cancer Survivorship, 12, 326–333. doi: 10.1007/s11764-017-0672-6 29318512 PMC5956036

[R57] Sun Y, Bao W, Liu B, Caan BJ, Lane DS, Millen AE, Simon MS, Thomson CA, Tinker LF, Van Horn LV, Vitolins MZ, & Snetselaar LG (2018). Changes in overall diet quality in relation to survival in postmenopausal Women with breast cancer: Results from the Women’s health initiative. Journal of the Academy of Nutrition and Dietetics, 118, 1855–1863. doi: 10.1016/j.jand.2018.03.017. e1856. 29859758

[R58] Sung H, Ferlay J, Siegel RL, Laversanne M, Soerjomataram I, Jemal A, & Bray F (2021). Global cancer statistics 2020: GLOBOCAN estimates of incidence and mortality worldwide for 36 cancers in 185 countries. CA: A Cancer Journal for Clinicians, 71, 209–249. doi: 10.3410/f.739487650.793592245 33538338

[R59] Sung H, Hyun N, Leach CR, Yabroff KR, & Jemal A (2020). Association of first primary cancer with risk of subsequent primary cancer among survivors of adult-onset cancers in the United States. *JAMA* : The Journal of the American Medical Association, 324, 2521–2535. doi: 10.1001/jama.2020.23130 33351041 PMC7756242

[R60] Tricco AC, Lillie E, Zarin W, O’Brien KK, Colquhoun H, Levac D, Moher D, Peters MDJ, Horsley T, Weeks L, Hempel S, Akl EA, Chang C, McGowan J, Stewart L, Hartling L, Aldcroft A, Wilson MG, Garritty C, Lewin S, Godfrey CM, Macdonald MT, Langlois EV, Soares-Weiser K, Moriarty J, Clifford T, Tunçalp Ö ., & Straus SE (2018). PRISMA extension for scoping reviews (PRISMA-ScR): checklist and explanation. Annals of Internal Medicine, 169, 467–473. doi: 10.7326/M18-085 30178033

[R61] Nations United. (2016). United nations decade of action on nutrition 2016–2025. Rome: Food and Agriculture Organization of the United Nations.

[R62] van Veen MR, Mols F, Bours MJL, Weijenberg MP, Kampman E, & Beijer S (2019). Adherence to the World Cancer Research Fund/American Institute for Cancer Research recommendations for cancer prevention is associated with better health–related quality of life among long-term colorectal cancer survivors: Results of the PROFILES registry. Supportive Care in Cancer, 27, 4565–4574. doi: 10.1007/s00520-019-04735-y 30927111 PMC6825038

[R63] van Zutphen M, Boshuizen HC, Kok DE, van Baar H, Geijsen AJMR, Wesselink E, Winkels RM, van Halteren HK, de Wilt JHW, Kampman E, & van Duijnhoven FJB (2019). Colorectal cancer survivors only marginally change their overall lifestyle in the first 2 years following diagnosis. Journal of Cancer Survivorship, 13, 956–967. doi: 10.1007/s11764-019-00812-7 31646463 PMC6881417

[R64] Wang F, Cai H, Gu K, Shi L, Yu D, Zhang M, Zheng W, Zheng Y, Bao P, & Shu XO (2020). Adherence to dietary recommendations among long-term breast cancer survivors and cancer outcome associations. Cancer Epidemiology, Biomarkers & Prevention, 29, 386–395. doi: 10.1158/1055-9965.epi-19-0872 PMC7007374 31871105

[R65] Wang Y, Chang A, Tan WP, Fantony JJ, Gopalakrishna A, Barton GJ, Wischmeyer PE, Gupta RT, & Inman BA (2021). Diet and exercise are not associated with skeletal muscle mass and sarcopenia in patients with bladder cancer. European Urology Oncology, 4, 237–245. doi: 10.1002/jcsm.12052 31133436 PMC6875605

[R66] Wirt A, & Collins CE (2009). Diet quality – what is it and does it matter? Public Health Nutrition, 12, 2473–2492. doi: 10.1017/s136898000900531x 19335941

[R67] World Cancer Research Fund/American Institute for Cancer Research. (2007). Food, nutrition, physical activity, and the prevention of cancer: A global perspective. Washington, DC: World Cancer Research Fund/American Institute for Cancer Research.

[R68] World Cancer Research Fund/American Institute for Cancer Research. (2018). Diet, nutrition, physical acitivity and cancer: A global persective, continous update project expert report 2018. World Cancer Research Fund/American Institute for Cancer Research.

[R69] World Health Organization. (2023). Progress report on the united nations decade of healthy ageing. Geneva: World Health Organization, 2021–2023.

[R70] Zheng X, Li X, Wang M, Shen J, Sisti G, He Z, Huang J, Li YM, & Wu A (2020). Second primary malignancies among cancer patients. Annals of Translational Medicine, 8. doi: 10.21037/atm-20-2059, 638–638. 32566575 PMC7290649

