# Supplementary material for: Dietary indices to measure diet quality in older cancer survivors: A scoping review on tools, their components and association with health outcomes
Source: Arch Gerontol Geriatr. Author manuscript; Available in PMC 2026 Mar 1. (PMC12068661; doi:10.1016/j.archger.2025.105797)
Supplement: S1-S5 tables [file NIHMS2075120-supplement-S1-S5_tables.docx]

**Table S4** Development, scoring and evaluation of Diet Quality Indices in older adult cancer survivors

|  | **Development** | | | | **Scoring information** | **Findings from study** |
| --- | --- | --- | --- | --- | --- | --- |
|  | **Country** | **Population (mean age, SD & sex) and Cancer type %** | **Dietary intake assessment** | **Evidence base used for Diet Quality Index or Dietary component (s) development** | **Scoring and weighting of components; Intepretation of scores** |  |
| **Healthy Eating Index 2005 (HEI-2005)** | | | | | | |
| Demark-Wahnefried et al. (2012) | USA/ Canada/ UK | Mean ages:  Immediate- Intervention Arm  73.0 ± 5.2 - Male 45.7 %; Female 54.3%  Delayed- Intervention Arm  72.9 ± 5.0 - Male 43.7 %;  Female 56.3%  Immediate Intervention:  Breast 45.7%; Prostate 40.7%  Colorectal 13.6%  Delayed Intervention:  Breast 44.9%; Prostate 38.4%  Colorectal 16.7%  Immediate- Intervention Arm (n=243). Delayed- Intervention Arm (n =245) Total: n=488 | Two unannounced 24-hour recalls | Existing Healthy Eating Index 2005 (HEI-05) (Guenther et al., 2008) based on 2005 DGA (U.S. Department of Health and Human Services and U.S. Department of Agriculture, 2005) | The HEI-2005 score was calculated for each participant using mean values of variables from the dietary recalls.  The HEI-2005 scores 12 components, using an energy- adjusted density approach to set standards (e.g., per 1,000 calories or as a percent of calories)  Total of 12 components: total fruit, whole fruit, total vegetables, dark green vegetables and orange vegetables and legumes, total grains, whole grains, milk, meat and beans, oils, saturated fat, sodium, calories from solid fats, alcoholic beverages and added sugars (SoFAAS). The latter three components are reverse- scored.  Each component contributes between 0 and 20 points. For each participant, each component was scored and calculated a total score (100 possible points), ranges from 0 (worst) to 100 (best) indicating diet quality and adherence to dietary guidelines | Change in Physical functioning  • The pace of decrease significantly slowed in both groups during the intervention, but in the immediate-intervention group, it accelerated in the year following the completion of the intervention.  • Diet quality is secondary outcome    Change in Targeted Behaviors and BMI  The group receiving immediate intervention showed positive changes from baseline to the first year, and these improvements remained consistent, with no notable variations between the first and second-year levels, except for a significant decrease in the consumption of fruits and vegetables.  • The delayed intervention group did not display significant enhancements across these measures from the baseline to the first year, but improvements were observed from the first to the second year during the intervention period.  • Both groups exhibited significant improvements Diet Quality, Physical activity, and BMI from the baseline to the two-year follow-up. |
| Mosher et al. (2009) | USA | Mean age 73 ±5    Female 52%; Male 48%  Female breast 43%; Prostate 42%; Colorectal 15%  n=753 | Two unannounced  24-hour diet recalls | As above | As above | Physical and mental QoL, include breakdown by sex and cancer type  • The study revealed a median of 10 minutes of weekly exercise, and only 7% achieved HEI-2005 scores over 80, indicating healthy eating practices according to national guidelines.  • All sex/cancer subgroups reported mental and physical QoL levels exceeding those of older cancer survivors and noncancer controls.  • Exclusion of survivors with significant comorbidities and functional limitations, coupled with the younger age of respondents, may partially explain the positive QoL findings.  • Associations between dietary/exercise habits, body weight status, and QoL outcomes showed that more weekly minutes of moderate-to-vigorous exercise correlated with improved physical QoL, including reduced pain and role limitations due to physical problems.  • The study identified positive associations between diet quality and physical functioning and vitality.  • In overweight and obese older cancer survivors, higher BMI was associated with poorer physical QoL across domains, but BMI showed no correlation with mental QoL.  • Further research is needed to assess the long-term impact of adherence to a low-fat, plant-based diet on various QoL indices among older cancer survivors. |
| Pelser et al. (2014) | USA | Mean age 68.4 SD unspecified,  Male 68.4%, Female 31.6%  Colon 73.6%, Rectal 26.4%  n=5,727 | Two food frequency questionnaire FFQ assessed diet (including alcohol consumption)  included 124-items | As above | As above | Survival after rectal cancer diagnosis  • Compared to normal-weight individuals, those who were obese had about a 19% increased risk of death from any cause, and approximately 84% increased risk of CVD death.  • There were also significant trends of decreased CVD mortality with increasing HEI-2005 scores and with alcohol consumption..  • A significant trend of decreased risk of colorectal cancer-specific mortality was observed with increasing HEI-2005 scores. However, pre-diagnosis physical activity and alcohol intake did not show associations with either all-cause mortality or colorectal cancer-specific mortality in colon or rectal cancer cases.  • Compared to those meeting one or fewer recommendations, colon cancer cases meeting all five recommendations had a 34% reduced risk of death, and rectal cancer cases had a 46% reduced risk of death.  • Higher lifestyle scores were also linked to a reduced risk of colorectal cancer mortality among rectal cancer cases, but not among colon cancer cases.  • No significant interactions were observed between the composite lifestyle score and sex, smoking, BMI, or cancer stage |
| **Healthy Eating Index 2010 (HEI-2010)** | | | | | | |
| Gopalakrishna et al. (2018) | USA | Mean age 73.6 ± 9.8,  Male 81%  Female 19%  bladder 100%  n=459 | One validated Diet History Questionnaire II (DHQ II,) involving 151 questions covering portion size over 134 food items and 8 supplements | Existing Healthy Eating Index 2010 (HEI-2010) (Guenther et al., 2013), which assesses adherence to the 2010 DGA (McGuire, 2011) | The HEI-2010 score was calculated for each participant using mean values of variables from the dietary recalls.  The HEI-2010 scores 12 components, using an energy- adjusted density approach to set standards (e.g., per 1,000 calories or as a percent of calories) Each component contributes between 0 and 20 points, and calculated a total score (100 possible points), ranges from 0 (worst) to 100 (best) indicating diet quality and adherence to Dietary guidelines.  Total of 12 components.  9 components (total fruit, whole fruit, total vegetables, green vegetables and beans, whole grains, dairy, total protein foods, seafood/plant proteins) focus on adequacy (dietary components to increase) and 3 (moderate intake of refined grains, sodium, so-called empty calories—added sugars, solid fats, alcohol, and an appropriate ratio of unsaturated to saturated fats­) focus on moderation (dietary components to decrease). | Health-related quality of life (HRQOL)  •Scores for both the overall HEI-2010 and its individual components closely aligned with the mean values observed in the U.S. population aged 65 years and older, as indicated by data from the 2011 to 2012 National Health and Nutrition Examination Survey.  •Similar to the majority of Americans, the cohort's total HEI-2010 score falls within the "needs improvement" category.  •Participants who underwent solely endoscopic treatments demonstrated higher diet quality compared to those who underwent cystectomy), although the absolute difference in  HEI-2010 scores is marginal.  •The participants exhibited limited consumption of whole grains and fat-soluble vitamins, with a notable deficiency in vitamin D intake.  •Initial analysis stated a significant correlation between diet quality and HRQOL, this association did not retain statistical significance in more comprehensive multivariate analysis.  •the Elixhauser Comorbidity Index demonstrated a significant association with HRQOL in the multivariate analysis, suggesting that comorbidities play a crucial role in influencing the quality-of-life outcomes for the participants. |
| Klassen et al. (2018) | USA | Mean age 65.4 ± 7.7  Male 76%  Female 24%    Breast 47%,  Prostate 38%,  Non-Hodgkin’s Lymphoma 15%  n=53 | Three unannounced 24-hour dietary recalls | As above | As above | Diet Quality  • Survivors adhering to a healthier diet tended to be female, possess higher socioeconomic resources, have a longer time since diagnosis, maintain a normal weight, and have no history of smoking.  • Qualitative discussions uncovered a more nuanced comprehension of dietary strategies among those practicing healthy eating habits, emphasizing the significance of household members in the decision-making process related to dietary choices.  • Among these cancer survivors, a substantial majority (89%) adhered to general adult guidelines for limiting alcohol consumption, and two-thirds reported consuming 5 or more servings of fruits and vegetables per day. However, only about half of the respondents met five of the recommendations, with percentages as follows: cholesterol (55%), total calories (51%), vitamin D (53%), calcium (49%), and saturated fat (40%).  • Two recommendations proved particularly challenging for this population. Only 15% of respondents achieved adequate fiber intake across their 3 recall days, and a mere 2% reported sodium consumption within recommended limits.  • The HEI-2010 reflected a diverse range among participants. Scores varied from a low of 36 (indicating numerous areas of poor nutrition) to 86, with an average value of 63 (similar to the US population average of 59). Among these 53 individuals, index scores and HEI-2010 scores showed a positive and moderate correlation.  • Younger participants showed a tendency toward higher average HEI 2010 scores but there were no differences in the number of recommendations met.  • No distinctions were noted between white and black participants, gender-based differences in dietary behaviors were evident.  • Women, on average, scored nearly 10 points higher on the HEI-2010 and met 1.2 more recommendations than men.  • Socioeconomic factors played a role, with participants attaining college or graduate education and higher income levels exhibiting higher HEI-2010 scores and meeting more dietary recommendations.  • Additional trends indicated higher HEI-2010 scores among respondents living alone, and those currently employed met a larger average number of recommendations.  • Differences in dietary habits based on cancer type mirrored those observed by gender, with breast cancer survivors meeting more dietary recommendations and having higher HEI-2010 scores than prostate cancer survivors.  • Survivors at least 5 years post-diagnosis demonstrated healthier eating habits than those with more recent diagnoses, and there was a trend toward higher HEI-2010 scores among individuals diagnosed at a younger age. |
| Wang et al. (2021) | USA | Mean age 73.8, SD unspecified, Male 79.6%, Female 20.4%  Bladder 100%  n=285 | One validated Diet History Questionnaire II (DHQ-II) food frequency questionnaire involving 151 questions covering portion size of over 134 food items and eight supplements. | As above | As above | Skeletal muscle mass (SMI) and sarcopenia  • Lifestyle factors including diet quality and physical activity are not associated with SMI and therefore appear to have limited impact on sarcopenia. Sarcopenia may largely be affected by nonmodifiable risk factors. HEI-2010 scores: Good (70%), Needs improvement (80%), poor (10%).  • Sarcopenia was observed in 72% of men and 55% of women among patients.  • Older age, male gender, and Black race were significant predictors of SMI based on univariate linear regression analysis.  • Tumor stage, grade, chemotherapy, and surgical procedures did not predict SMI.  • Modifiable lifestyle factors, including total physical activity, strenuousness of physical activity (high, moderate, low), individual nutritional components (daily calories, fat, carbohydrates, protein), and HEI-2010 diet quality, did not show an association with SMI according to multivariate linear regression analysis. |
| Sun et al. (2018) | USA | Mean age 65.0 ±6.9  Female 100%  Breast 100%  n=2,295 | OS participants:  Three self-administered developed and validated FFQ (adapted from the Health, Habits and Lifestyle Questionnaire) with 122 items  66% WHI DM participants  Two self-administered developed and validated FFQ, as above.  33% WHI DM participants  Completed an FFQ on a rating bases each year from Years two to nine, self-administered developed and validated FFQ with 122 items | As above | As above and additionally - The changes of HEI-2010 scores were categorized into 3 groups: improved dietary quality defined as 15% (one standard deviation in changes of diet quality) increase in the score. Relatively stable dietary quality defined as 14.9% change in the score. Worsening dietary quality defined as 15% decrease in the score. | Risk of death and diet quality:  • Over 12 years of follow-up, there were a total of 763 deaths recorded, among which 242 were attributed to breast cancer while 521 were due to other causes.  • After being diagnosed with breast cancer, 72% of the women maintained a relatively stable diet quality, with only a 14.9% change in their HEI-2010 score. • • About 9% experienced a decrease in diet quality, marked by a decline of more than 15% in the HEI-2010 score, while 19% saw an increase in diet quality with a similar increase in the score.  • Comparing these groups, those who experienced a decrease in diet quality tended to be older, had higher diet quality before diagnosis, lower education levels, and were more likely to increase total energy and alcohol intake after diagnosis.  • Those who improved their diet quality were younger, had lower diet quality before diagnosis, had higher education levels, and were more inclined to decrease total energy and alcohol intake post-diagnosis.  • The average diet quality of the study population increased from 66.0 to 67.6 after being diagnosed with invasive breast cancer.  • Women who experienced a decrease in diet quality had a significantly higher risk of death from breast cancer compared to those who maintained stable diet quality.  • Increasing diet quality did not show significant associations with the risk of death from any causes, breast cancer or other causes.  • These findings remained consistent even after adjusting for changes in BMI over time.  • No significant variations were observed based on factors like smoking status, postmenopausal hormone therapy, cancer characteristics, obesity, or changes in physical activity.  • The quality of diet before diagnosis did not seem to affect the risk of death from any causes, breast cancer or other causes.  • Higher diet quality post-diagnosis was associated with a reduced risk of death from causes other than breast cancer. |
| **Healthy Eating Index 2015 (HEI-2015)** | | | | | | |
| Krok-Schoen et al. (2021) | USA | Mean age 74.50 ± 8.43  Female 100%  Breast 67.7%,  Hematologic 13.5%,  Gynecologic 12.0%,  Other 6.8%  n=171 | One Diet History Questionnaire II (DHQ II), a validated food frequency questionnaire FFQ containing of 134 food item questions and 8 dietary supplement questions | Existing Healthy Eating Index 2010 (HEI-2015), which assesses adherence to the 2015 DGA (US Department of Health and Human Services and US Department of Agriculture, 2015) | Total of 13 components, using an energy- adjusted density approach to set standards (e.g., per 1,000 calories or as a percent of calories)  9 components (total fruit, whole fruit, total vegetables, greens and beans, whole grains, dairy, total protein foods, seafood/plant proteins, Fatty acids) focus on adequacy (dietary components to increase) and 4 (moderate intake of refined grains, sodium, so-called empty calories—added sugars, solid fats, alcohol, and saturated fats­) focus on moderation (dietary components to decrease).  Each component contributes between 0 and 10 points  Total score of 100 and standards for scoring  For each participant, each component was scored and calculated a total score (100 possible points), ranges from 0 (worst) to 100 (best) indicating diet quality and adherence to Dietary guidelines | Dietary intake and associations with physical and mental HRQoL  • The results highlighted various challenges in maintaining healthy lifestyles among older cancer survivors. In this study, older female cancer survivors exhibited low levels of both physical and mental Health-Related Quality of Life (HRQoL), engaged in limited physical activity, and tended to be overweight. • HRQoL was adversely affected by poor diet quality, high rates of overweight or obesity, and insufficient levels of physical activity.  • Married older female cancer survivors with a normal weight, higher incomes, no unintentional weight loss, elevated HEI total scores, and involvement in strenuous and/or moderate exercise exhibited notably higher mean Physical Component Summary scores  • The study results revealed a need for enhancement in lifestyle behaviors among older female cancer survivors.  • Nearly 70% of the participants reported a lack of mild exercise and were categorized as overweight or obese.  • Older female cancer survivors with at least a college degree, retired status, and no unintentional weight loss demonstrated significantly higher mean Mental Component Summary scores.  • The participants achieved an average HEI-2015 score of 66.54 ± 10.0 out of 100, with scores ranging from 34.6 to 89.8. This score was shaped by a substantial intake of total vegetables, whole fruits, total protein foods, seafood, and plant proteins and a limited consumption of whole grains and an elevated intake of saturated fat. |
| Pisegna et al. (2021) | USA | Mean 71.3 ± 8.1  Female 100%  Breast 100%  n=90 | One validated Diet History Questionnaire II (DHQII) is a FFQ, containing of 134 food item questions and 8 dietary supplement questions |  |  | • The study participants were predominantly non-Hispanic white (90.0%) and overweight, with a mean BMI of 28.2.  • About 66.8% of participants were classified as overweight or obese. A small percentage (4.4%) reported concerns about food security, while 25.6% scored 2 or more on the Malnutrition Screening Tool (MST), indicating a risk for malnutrition.  • Participants demonstrated a diet quality classified as "needs improvement" by HEI-2015 scoring, with a mean HEI-2015 score of 67.02 ± 9.74.  • On average, participants reported consuming one serving each of fruits and vegetables per day.  • Mean emotional well-being and social functioning composite scores were 63.6 ± 10.2 and 83.5 ± 19.6, respectively.  • Significant positive associations were observed between HEI-2015 scores and social functioning composite scores, as well as HEI-2015 scores and education level.  • Simple linear regression analyses showed that social functioning composite scores accounted for 4.5% of the variance in HEI-2015 scores, while education level accounted for 4.3% of the variance.  • Hierarchical linear regression analyses were conducted to explore associations among predictor variables of MHRQoL variables (comprising emotional well-being and social functioning composite scores), food insecurity, malnutrition risk, and demographic variables (race/ethnicity, education, income, marital status, and age), with HEI-2015 scores as the dependent variable.  • The overall hierarchical regression model did not reveal statistically significant associations when controlling for demographic characteristics.  • Individuals with at least a bachelor's degree exhibited significantly higher HEI-2015 scores compared to those with less than a bachelor's degree. |
| Schmalenberger et al. (2022) | USA | Mean age 66.6 ±9.4  Female 100%  Breast 67.7%, Hematologic 12.8%,  Gynecologic 12.0%,  other cancers include lung, connective tissue, skin, lymph node, bone, maxillary sinus 7.5%  n=171 | One the Diet History Questionnaire (DHQ) II,  is a validated food frequency questionnaire FFQ containing of 134 food item questions and 8 dietary supplement questions |  |  | Physical functioning, Income and education were significantly associated with dietary quality  • The majority of participants were of white ethnicity (90%), married (55%), retired (83%), and possessed at least a college degree (55%).  • The predominant cancer types were breast (68%) and hematologic.  • A significant proportion of cancers were categorized as stage 1A/1B (30%) and stage 2A/2B (24.5%). Women exhibited a Body Mass Index (BMI) ranging from 17 to 51 kg/m², with a mean BMI of 27.7 kg/m².  • The average Malnutrition Screening Tool (MST) score was 1.0 ± 1.8, indicating that 72% of participants were not considered at risk for malnutrition.  • The mean physical functioning score was 59.8 ± 24.1. Most participants rated their health as very good (42%) or good (40%). • The mean albumin levels were 4.0 g/dL ± 0.4 g/dL, and the mean blood levels of C-reactive protein (CRP) were 14.9 mg/L ± 36.4 mg/L, with limited documentation of CRP in participants' medical records.  • Participants obtained a mean HEI-2015 score of 66.54 ± 10.01 out of 100, with scores ranging from 34.58 to 89.81 out of 100.  • This was notably influenced by a high consumption of total vegetables, whole fruits, total protein foods, seafood, and plant proteins, coupled with a low consumption of whole grains and high consumption of saturated fat.  • Among the adequacy components, whole grains had the lowest mean score of 2.70 out of a possible 10 points, whereas whole fruit had the highest mean score of 4.53 out of a possible five points. Total protein food was the next highest component with a mean score of 4.57 out of a possible five points.  • Concerning the moderation components, sodium had the lowest mean score of 5.02 out of a possible 10 points, and saturated fat followed as the second lowest component with a mean score of 5.36 out of a possible 10 points.  • Positive correlations were identified between total HEI-2015 scores and physical functioning, self-rated health, albumin, education, and income. A moderate positive correlation was observed between CRP and BMI.  • Negative correlations were noted between physical functioning and BMI, while positive correlations were found between physical functioning and education and income.  • Individuals with higher HEI-2015 scores were more likely to report higher physical functioning, although other characteristics were not significantly associated with HEI-2015 scores. |
| **Brazilian Healthy Eating Index—Revised (BHEI-R)** | | | | | | |
| Mazzutti et al. (2021) | Brazil | Median age 65 years  Female 100%  Breast 100%  n=89 | Nine 24HRs recalls, the first 24HR was carried out in person and the others via telephone | Existing Brazilian Healthy Eating Index—Revised (BHEI-R**) (**Previdelli et al., 2011) is based on HEI-2005 (Guenther et al., 2008) incorporating the recommendations by 2006 Dietary Guidelines for the Brazilian Population (MInistery of Health Brazil/Secretariat of Health Care Primary Health Care Department, 2006) | Data with household measurements from the 24HRs were converted to units of measurement (grams or milliliters) by the Table to Evaluate Food Consumption in Household Measurements to calculate the number of servings. The number of daily servings was adjusted by 1000 kcal/day.  The scores for each food component and the BHEI-R total score were calculated. For most components, the recommendations by the Food Guide for the Brazilian Population regarding the  number of daily servings were considering when adopting the criteria for establishing the minimum, intermediate and maximum scores.  BHEI-R includes 12 components, each component contributes between 0 and 20 points  The maximum BHEI-R total score is 100 points.  For each participant, each component was scored and calculated a total score (100 possible points), ranges from 0 (worst) to 100 (best) indicating diet quality and adherence to Dietary guidelines  Inadequate diet scores below 58.46; diet required modifications scores below 64.38; healthy diet scores equal to or greater than 64.38. A total BHEI-R score below 64.38 as a cardiovascular risk factor | • The findings from this prospective study reveal a significant prevalence of multiple risk factors associated with cardiovascular diseases (CVDs).  • In the evaluation of the BHEI-R components, a significant in the intake of the Meat, Eggs, and Legumes components was observed from T0 to T1 , as determined by post hoc analysis.  • Similarly, there was an significant increase in the consumption of the Oils component between T1 and T2.  • However, no significant alterations were found in the other categories.  • In terms of the quantitative nutrient analysis, there was a decrease in energy intake, total carbohydrates, sugars, omega-6, total and monounsaturated fats observed at T1 and T2 compared to T0.  • Furthermore, there was a reduction in polyunsaturated fat consumption at T1 compared to T0. At T2, there was a decline in saturated fats and cholesterol intake, along with an increase in sodium and omega-3 consumption.  • Additionally, a decrease in protein, trans fats, and the omega-3/omega-6 ratio was observed across all three time points. Total fiber consumption remained unchanged over tim, but there was a decrease in soluble fiber at T2 compared to T0 and T1. |
| **Revised Healthy Eating Index (RHEI)** | | | | | | |
| Miller et al. (2008) | USA | Median age 73 years  Female 52%, Supplement use 78%  Male 48%  Supplement use 70%  Female breast 43%,  Prostate 42%, Colorectal 15%  n=753 | Two unannounced  24-h diet recalls | Existing Revised Healthy Eating Index RHEI (Guenther et al., 2006), was based on the  HEI-2005 (Guenther et al., 2008) and 2005 DGA (U.S. Department of Health and Human Services and U.S. Department of Agriculture, 2005) | Total of 12 individual scored components.  The following six components have a maximum score of five: Total Fruit, Whole Fruit, Total Vegetables, Dark Green and Orange Vegetables and Legumes, Total Grains, and Whole Grains. Milk, Oil, Meat and Beans, Sodium, and Percent Calories from Saturated Fat have a maximum score of 10.  The remaining component of Calories from Solid Fat, Alcohol, and Added Sugar has a maximum score of 20.  Revised Healthy Eating Index (HEI)  includes 12 components, each component contributes between 0 and 20 points  The maximum Revised Healthy Eating Index (HEI) score is 100 points  For each participant, each component was scored and calculated a total score (100 possible points), ranges from 0 (worst) to 100 (best) indicating diet quality and adherence to Dietary guidelines | Diet quality as a factor associated with dietary supplement use  • Bivariate analyses identified characteristics associated with supplement use (e.g., age, gender, cancer type, education, tobacco use, total HEI score).  • Multivariate logistic regression included significant variables to predict supplement use patterns.  • Higher RHEI scores in Total Fruit, Whole Grain, and Oil components of RHEI were associated with increased supplement use.  • Higher scores in the Meat and Beans category were linked to decreased likelihood of supplement use.  • Individuals with professional or graduate degrees were significantly more likely to use supplements compared to those with less than a high school education.  In a study of older cancer survivors (≥65 years, ≥5 years post-diagnosis):  • 74% reported supplement use, ranging from 70% in colorectal cancer survivors to 80% in breast cancer survivors.  • Common supplements included MVMM products (60%), calcium/vitamin D (37%), antioxidants (30%), fatty acids/oils (21%), and glucosamine/chondroitin (14%).  • Supplement use correlated with various demographic, disease, and health-related characteristics, indicating a potential marker for other health-related behaviors.  • Supplement users and nonusers had similar BMI and physical activity distributions, possibly due to screening criteria focusing on overweight or obese older adults.  • Small proportions of supplement users exceeded Tolerable Upper Intake Levels (ULs):  • About 10% exceeded the UL for zinc.  • 4% or less exceeded ULs for vitamins A, B6, C, and D, folic acid, calcium, iron, and magnesium.  • No individuals exceeded the UL for vitamin E; ULs not established for vitamins B12 and K, or potassium.  • Among prostate cancer survivors using supplements:  • 27% had calcium intakes exceeding 1,500 mg per day.  • Only 5% met the Adequate Intake (AI) for calcium from diet alone, suggesting the need for tailored guidance on supplement doses for this subgroup. |
| Snyder et al. (2009) | USA | Mean age: 73.4 ± 5.4,  Female 50.1%, Male 49.9%  Breast 40.1%; Prostate 42.5%; Colorectal 17.4%  n=641 | Three random, 24-h recalls (one each at baseline, one and two-year follow-up) | As above | As above | Physical functioning:  • A higher percentage of them were white and female breast cancer survivors.  • The majority of survivors were initially diagnosed with localized disease.  • Initially, 753 subjects were identified as eligible through the screener, and after completing the two baseline surveys, an additional screening on BMI and/or 150 minutes of moderate to vigorous exercise per week led to 112 subjects being deemed ineligible.  • In the end, 641 participants were successfully enrolled in the RENEW program. Currently, approximately half of the participants are undergoing the intervention and completing follow-up. |
| **The Diet Quality Index Revised (DQI-R)** | | | | | | |
| Demark-Wahnefried et al. (2003) | USA | [Study protocol]  Mean age  71.7 ±-4 .9,  Female 57.6% Male 42.4%  Prostate and female breast, % unspecified  n=158 | Three 24-hour dietary recalls (once each at baseline, 6 months and 12 months) | Existing Diet Quality Index Revised (DQI-R) by Haines et al. (1999) | DQI-R includes on ten subcategories of ten points each, which include intakes of: total fat; saturated fat, cholesterol; fruit; vegetables; grains; calcium; iron; diet diversity ((consumption across 23 food groups); and dietary moderation (composite score of alcohol, discretionary fat, added sugars and sodium).  Since the intervention did not target alcohol, sodium or added sugar, this subcategory is assigned a five-point constant. Total score unspecified. A higher score thus indicated a higher adherence to recommendations. | Physical functioning  [Study protocol] |
| Demark-Wahnefried et al. (2006) | USA | Intervention age mean 71.5 +/- 4.4  Female 57.3%, Male 42.7%  Attention Control mean age 71.9 +/- 5.6  Female 57.0%, Male 43.0%  Intervention:  female breast cancer 57.3%  prostate cancer 42.7%  Attention control:  female breast cancer 57%  prostate cancer 43%  n=182 | Three 24-hour dietary recalls (once each at baseline, 6 months and 12 months) | As above | DQI-R includes on ten subcategories of ten points each, which include intakes of: total fat; saturated fat, cholesterol; fruit; vegetables; grains; calcium; iron; diet diversity (consumption across 23 food groups); (and dietary moderation (composite score of alcohol, discretionary fat, added sugars and sodium).  Since the intervention did not target alcohol, sodium or added sugar, this subcategory is assigned a five-point constant.  Total score unspecified. A higher score thus indicated a higher adherence to recommendations | Physical functioning  • The majority of participants expressed readiness to make changes in their diet and exercise habits, displaying high levels of confidence in achieving these goals. Notably, readiness and confidence were more pronounced for dietary changes than for exercise.  • Initially, there were no notable differences between the study arms at the baseline assessment.  • Comparing baseline data with the 6-month follow-up, the intervention exhibited a statistically significant enhancement in diet quality and an increase in QOL for both study arms.  • While the overall quality of the participants' diets improved with the intervention, there were no significant alterations observed in specific food groups or dietary components. Similarly, no significant changes were noted in the already high baseline levels of self-efficacy or the stage of readiness for adopting a healthy diet.  • QOL showed improvement and stabilization at higher levels in both the intervention and control arms.  • The agreement between BMIs calculated from self-reported weights and heights and those derived from clinical assessments was excellent. |
| Clutter Snyder et al. (2007) | USA | Intervention:  n=89  Mean age 71.5 ±4.4  Attention Control:  n=93  Mean age 71.9 ±5.6  Intervention:  female breast cancer 57%,  prostate cancer 43%  Attention control:  female breast cancer 57%,  prostate cancer 43%  n=182 | Three 24-h diet recalls (once at baseline, once at at completion of the intervention period 6 months and once at 12-month follow-up); | As above | DQI-R includes on ten subcategories of ten points each, which include intakes of: total fat; saturated fat, cholesterol; fruit; vegetables; grains; calcium; iron; diet diversity (consumption across 23 food groups); and dietary moderation (composite score of alcohol, discretionary fat, added sugars and sodium).  Dietary diversity and moderation components were assigned continuous scores on a 10-point scale.  Each component contributes between 0 and 10 points.  For each participant, each component was scored and calculated a total score (100 possible points  A higher score thus indicated a higher adherence to recommendations. | • 85% of participants reported energy intakes ranging from 500 to  • 1,800 kcal/day based on the average of three 24-hour recalls.  • At baseline, the DQI-R scores ranged from 27.2 to 92.7, with a mean of 67.6±13.4 (out of 100 points).  • Initial DQI-R scores were similar between study arms and followed a normal distribution.  • After 6 months, participants in the intervention arm significantly enhanced their average DQI-R scores compared to the control group.  • Except for dietary diversity at both 6 and 12 months and calcium intake at 12 months, no significant changes were observed over time in any of the subscores.  • While diet diversity was the only subcomponent showing significant differences between arms over time, no such differences were noted in any of the 23 food subgroups comprising this composite measure.  • Initially, more than half of the participants adhered to guidelines for cholesterol and iron intake.  • Roughly 40% of participants met guidelines for total fat and saturated fat, but less than one-third met guidelines for calcium, grains, and fruit and vegetable consumption.  • Although the intervention arm exhibited higher goal attainment scores for total fat, saturated fat, cholesterol, fruit servings, and iron at 6 months compared to the attention control arm, these differences lacked statistical significance.  • Regardless of study arm, participants were less likely to meet guidelines for vegetable and grain servings at the 6-month mark.  • No significant changes in weight status were observed either between or within study arms over time. |
| **Mediteranean Diet Score (MDS)** | | | | | | |
| Bauer et al. (2018) | USA | Mean age 68 **±** 7,  Male 100%  Prostate 100%  n=2,960 | One validated FFQ with 130 items | Mediterranean Diet Score developed by Trichopoulou et al. (2003) | Mediterranean Diet Score  1 point each for consuming less than the median dairy and meat intake calculated separately for each dietary questionnaire cycle  1point for alcohol intake between 10 and 50 g/d;  1 point each for being above the median intake of vegetables, legumes, fruits and nuts, grains, fish, and the ratio of polyunsaturated to saturated lipids  Monounsaturated fat, used in the traditional Mediterranean Diet Score was not used for the lipid ratio.  The 9 components are summed up from 0-9; higher score indicates closer adherence to the Mediterranean diet. | Urinary incontinence, urinary irritation/obstruction among men with history of BPH/LUTS, urinary irritation/obstruction among men without history of BPH/LUTS, sexual function:  • No significant connection between the Mediterranean Diet Score after diagnosis and HRQOL (urinary or sexual function).  • Nonetheless, a slight correlation was identified between increased post-diagnostic vegetable consumption and improved urinary function, as reflected in lower urinary incontinence scores.  • A connection was observed between higher post-diagnosis vegetable intake and lower polyunsaturated fat intake, leading to slightly higher scores in urinary irritation/obstruction, indicating improved urinary function. Notably, these associations were confined to individuals with a history of benign prostatic hyperplasia (BPH) or lower urinary tract symptoms (LUTS) before their prostate cancer diagnosis. |
| **Dutch Healthy Diet Index score (DHD-index)** | | | | | | |
| Breedveld-Peters et al. (2018) | The Netherlands | Mean age 70 ±8·7,  Male 63 %  Female 37 %  Colorectal 100%  n=145 | 1 each for dietary intake and supplement use using 7-day food diaries, information on supplement use, type and brand, ingredients, dosage and frequency of use was recorded and registered in detail during home visits | Existing Dutch Healthy Diet Index (DHD-Index) (van Lee et al., 2012) is based on the Dutch Recommendations for a healthy Diet 2006 (Health Council of the Netherlands, 2006) | Adherence to these recommendations was scored using the previously published Dutch Healthy Diet Index score (DHD-i), which is a continuous score with ten components that represent the Dutch Recommendations for a healthy Diet (2006) includes one PA component and nine dietary recommendations.  9 Dietary components:Vegetables, fruit and fruit juice intake, dietary fibre, fish, SFA, sodium, alcohol, trans fatty acid, foods and beverages that contain easily fermentable sugars and drinks.  10 components summed up ranges from 0 (worst) to 10 (best) indicating diet quality and adherence to Dietary guidelines | QOL and physical functioning  • Significant associations of a higher DHD-index adherence score with better global QoL and physical functioning in the overall group; with better global QoL, better physical functioning and less disability in women only; and with better physical functioning in overweight/obese CRC survivors only |
| **9-item index** | | | | | | |
| Klassen et al. (2018) | USA | mean age 65.4 ±7.7  Male 76%  Female 24%    Breast 47%,  Prostate 38%,  Non-Hodgkin’s Lymphoma 15%  n=53 | Three unannounced 24-hour dietary recalls | 9-item index developed by Klassen et al. (2018) is based on 2010 DGA and other cancer-relevant recommendations from the American Cancer Society (Kushi et al., 2012) | Averages were calculated across the three 24-hour recalls for 9 dietary components, based on broadly accepted dietary recommendations to prevent cancer and other chronic diseases, and assigned a value of 0 or 1 to each respondent, based on meeting recommendations.  The 9 dietary behaviors included no more than moderate intake of 5 unhealthful dietary components (alcohol intake, cholesterol, total calories, saturated fat and sodium) and adequate intake in 4 components (fruit and vegetables, vitamin D, calcium and fiber.  Participant scores of 0 or 1 on the nine elements were summed for a composite score, indicating number of recommendations met. 9 dietary components were summed up ranges from 0 (worst) to 9 (best) indicating diet quality and adherence to Dietary guidelines | • Survivors adhering to a healthier diet tended to be female, possess higher socioeconomic resources, have a longer time since diagnosis, maintain a normal weight, and have no history of smoking.  • Among these cancer survivors, a substantial majority (89%) adhered to general adult guidelines for limiting alcohol consumption, and two-thirds reported consuming 5 or more servings of fruits and vegetables per day.  • About half of the respondents met five of the recommendations, with percentages as follows: cholesterol (55%), total calories (51%), vitamin D (53%), calcium (49%), and saturated fat (40%).  • Only 15% of respondents achieved adequate fiber intake across their 3 recall days, and a mere 2% reported sodium consumption within recommended limits.  • When assessing the summed index scores for respondents, results ranged from 1 to 8, indicating that all respondents met at least one dietary recommendation, but none achieved all nine.  • The median number of recommendations met was 4. |
| **The World Cancer Research Fund (WCRF)/American Institute for Cancer Research (AICR) 2007 Recommendations (WCRF/AICR 2007)** | | | | | | |
| Breedveld-Peters et al. (2018) | The Netherlands | Mean age 70 ± 8·7  Male 63 %  Female 37 %  Colorectal 100%  n=145 | 1 each for dietary intake and supplement use using 7-day food diaries, and information on supplement use | Based on the existing World Cancer Research Fund American Institute for Cancer Research (WCRF/ AICR) 2007 recommendations (WCRF/ACIR, 2007) and scores were calculated based on the Dutch Food Composition Table | WCRF/AICR recommendations included in this study are body fatness, PA, energy-dense foods, plant foods, meat consumption, alcoholic drinks and dietary supplements.  This study did not operationalize following recommendations on:  Fast food and starchy food, (as no international agreement on the definitions and overlap with energy dense foods) salt restriction (as difficult to measure and not available), avoid mouldy cereals and pulses and breast feeding (not appliable for population) .  The WCRF/AICR dietary recommendation scores were calculated based on the Dutch Food Composition table data  To calculate the WCRF/AICR adherence score, each recommendation was assigned a score of 1 point for complete adherence, 0·5 points for moderate adherence and 0 points for non-adherence.  Predefined cut-off values of the WCRF/AICR recommendations adherence categories were used  By summing the scores of the ten operationalised (sub) recommendations, an overall adherence score for the WCRF/AICR lifestyle recommendations was calculated (score range: 0–10 points). A higher score thus indicated a higher adherence to recommendations. | 1) WCRF/AICR-2007 lifestyle recommendations for cancer prevention, association of adherence with relevant HRQoL outcomes, including global QoL, physical functioning, fatigue, disability and distress  •Considerable variation in adherence levels across individual WCRF/AICR-2007 recommendations was observed, indicating that different lifestyle factors contribute differently to individuals' overall scores.  •Moderate adherence was prevalent for various individual lifestyle items, such as BMI, sedentary behaviour, sugary drink consumption, and fruit and vegetable intake.  •Adherence was significiantly low for certain recommendations, limiting red and processed meat consumption and energy-dense foods, while it was high for the recommendation to limit alcohol intake. |
| Inoue-Choi et al. (2014) | USA | Mean age 78.9 ±3.9  Female 100%  Breast 45.7%  Colorectal 18.8%,  Gynecologic 13.5%,  Other cancer 22.0%  n=2,118 | One validated Harvard food frequency questionnaire (FFQ) to report usual intake of 127 food items | As above | WCRF/AICR recommendations included in this study were for sugary drinks, fruits and vegetables, fiber, red and processed meat, alcohol, and sodium  Predefined cut-off values of the WCRF/AICR recommendations adherence categories were used  This study did not operationalize following recommendations on:  Body fatness, physical activity, Limit consumption of energy dense foods, avoid mouldy cereals and pulses, dietary supplements, breast feeding and recommendations for cancer prevention after treatment.  Scores for the 6 dietary recommendations (increasing total fruit and vegetable and dietary fiber intake, and limiting red meat and processed meat product, alcohol, and sodium intake) were summed as a dietary quality score (maximum score =6)  To calculate the WCRF/AICR adherence score, each recommendation was assigned a score of 1 point for complete adherence, 0·5 points for moderate adherence and 0 points for non-adherence. A higher score thus indicated a higher adherence to recommendations  Scores for the seven indicators were summed as the recommendation adherence score (0-6). | All-cause mortality: •Differences in characteristics of cancers were not observed between users and nonusers of dietary supplements.  • Among cancer survivors, those with lower diet quality scores were more likely to be current smokers, physically inactive, diabetic, perceive poorer general health, have survived for shorter periods, and undergo cancer therapy, compared to those with higher diet quality scores.  • The average diet quality score was 4.5 (range: 2.0–6.0), and it did not vary between supplement users and nonusers.  •Dietary supplement users, compared to nonusers, exhibited slightly higher intake of protein, fruits and vegetables, and whole grains.  • The intake of micronutrients, such as vitamins A, D, and iron, was also higher among supplement users, although differences appeared to be minimal.  • The total intake (combining dietary and supplemental sources) of all micronutrients was higher in supplement users.  •Cancer survivors with low diet quality scores had lower intake of total energy, protein, carbohydrates, fruits and vegetables, total meat, and whole grains and had higher alcohol intake compared to those with high diet quality scores.  • Fat intake (both total and saturated) did not differ between low and high diet quality score groups.  •Up to 2010, 608 deaths were identified, with approximately 85% of cancer survivors using dietary supplements.  • Overall supplement use and multivitamin use were not linked to mortality. Iron supplement use was associated with a 39% higher risk of death, especially among survivors with deteriorating general health.  • Folic acid supplement use was associated with a higher risk of death, particularly among survivors reporting a low-quality diet.  •Multivitamin use and using a greater number of supplements showed a trend towards higher mortality, but only among those with poor diet quality.  • Vitamin E supplements in combination with a multivitamin was associated with a lower risk of death, specifically among survivors with higher dietary vitamin E intake. |
| (Inoue-Choi et al., 2013a) | USA | Mean age 78.9 ± 3.9  Female 100%  Breast 47.2%  Colorectal 17.4%,  Gynecologic 12.9%,  Other 22.5%  n=2,193 | One validated Harvard food frequency questionnaire to report usual intake of 127 food items | As above | WCRF/AICR recommendations included in this study are body fatness, PA, plant foods, meat consumption, alcoholic drinks and sodium intake.  Predefined cut-off values of the WCRF/AICR recommendations adherence categories were used.  This study did not operationalize following recommendations on:  Limit consumption of energy dense foods and drinks, avoid mouldy cereals and pulses, dietary supplements, breast feeding and recommendations for cancer prevention after treatment.  Scores for the 5 dietary recommendations (increasing total fruit and vegetable and dietary fiber intake, and limiting red meat and processed meat product, alcohol, and sodium intake) were summed as a dietary quality score (maximum score =5)  To calculate the WCRF/AICR adherence score, each recommendation was assigned a score of 1 point for adherence and 0 points for non-adherence.  Scores for the seven indicators were summed as the recommendation adherence score (0-7).  The adherence score was separated into 3 components of the WCRF/AICR guidelines: body weight (0 to 1), physical activity (0 to 1), and diet (0 to 5). A higher score thus indicated a higher adherence to recommendations | HRQOL • The average adherence score was 4.0 ± 1.2. In general, a stronger adherence to the WCRF/AICR-2007 guidelines showed a significant correlation with improved Physical Component Summary (PCS) and Mental Component Summary (MCS) scores, even after accounting for factors such as age, education, marital status, comorbidities, smoking, cancer stage, and ongoing cancer treatment  • Specifically, individuals with adherence scores of 5 or higher had higher PCS and MCS (compared to those scoring 3 or lower.  • Adhering to the physical activity recommendation was linked to elevated PCS and MCS, considering demographic and medical variables, body mass index, and adherence to dietary recommendations.  •Following the body weight recommendation was associated with higher PCS but lower MCS.  •Adherence to dietary recommendations was only linked to higher MCS.  • Elevated PCS scores were observed specifically in association with adherence to fruit and vegetable and dietary fibre recommendations, even after adjusting for BMI and physical activity level.  •MCS scores were higher only when adhering to recommendations for reduced red meat and sodium intake.  •Adherence to the recommendation for reduced alcohol intake did not show a significant association with either PCS or MCS after adjusting for BMI and physical activity level. |
| Inoue-Choi et al. (2013b) | USA | Mean age 78.9 ±3.9  Female 100%  Breast 46.5%,  Colorectal 18.8%,  Gynecologic 13.0%,  Other 21.7%  n=2,017 | One validated Harvard food frequency questionnaire, to report usual intake of 127 food items | As above | WCRF/AICR recommendations included in this study are body fatness, PA, energy dense drinks, plant foods, meat consumption, alcoholic drinks and sodium intake.  Predefined cut-off values of the WCRF/AICR recommendations adherence categories were used.  This study did not operationalize following recommendations on:  Limit consumption of energy dense foods, avoid mouldy cereals and pulses, dietary supplements, breast feeding and recommendations for cancer prevention after treatment.  Scores for the 8 indicators were summed as the recommendation adherence score (0-8). A higher score thus indicated a higher adherence to recommendations  The adherence score was separated into 3 components of the WCRF/AICR guidelines: body weight (0 to 1), physical activity (0 to 1), and diet (0 to 6 ). | • All-cause (n = 461), cancer-specific (n=184), and cardiovascular disease (CVD)-specific mortality (n=145) were compared by the total adherence score and by adherence scores for each of the three components of the recommendations.  • Older female cancer survivors adhering to more WCRF/AICR-2007 guidelines for cancer prevention exhibited lower risks of all-cause mortality.  • The association potentially extended to cancer-specific mortality but did not include cardiovascular disease (CVD)-specific mortality.  • Within the three WCRF/AICR 2007 guideline components (body weight, physical activity, and diet), adherence to the physical activity recommendation showed the strongest correlation with reduced risk for all mortality outcomes.  • Stratification by cancer type revealed lower death risk among breast cancer survivors, while this association was not observed in colorectal and gynecologic cancer survivors.  • Although not statistically significant, survivors of other cancers with higher guideline adherence (score of 6 or above out of 8) had a lower risk of mortality compared to those with lower adherence (score of 4 or less).  • Among breast cancer survivors and those with other cancer types, women with greater adherence to guidelines showed a weaker but discernible trend of lower cancer-related mortality.  • Adherence to dietary recommendations was linked to a lower risk of all-cause mortality, but not specifically to cancer-specific or CVD-related mortality.  • Adherence to the body weight recommendation was associated with a higher risk of all-cause mortality.  •These findings highlight the importance of adopting a healthy lifestyle post-cancer diagnosis for better survival among older cancer survivors, irrespective of pre-diagnosis health behaviors. |
| van Veen et al. (2019) | The Netherlands | 70.8 + 9.2 years,  Male 58%, Female 42%  Colon 58%  Rectum 42%  n=1,096 | Two WCRF/DHD-FFQ consists of 40 items.  The WCRF/DHD-FFQ was adapted from the Dutch Healthy Diet-Food Frequency Questionnaire (DHD-FFQ) | As above | WCRF/AICR recommendations included in this study are  body fatness, physical activity, foods, and drinks that promote weight gain, plant-based foods, meat products, alcoholic drinks, preservation/processing/preparation of foods, and dietary supplement use.  Predefined cut-off values of the WCRF/AICR recommendations adherence categories were used.  This study did not operationalise:  avoid mouldy cereals and pulses, breast feeding recommendations for cancer prevention after treatment.  To calculate the WCRF/AICR adherence score, each recommendation was assigned a score of 1 point for complete adherence, 0·5 points for moderate adherence and 0 points for non-adherence. A higher score thus indicated a higher adherence to recommendations.  Scores for the 8 indicators were summed as the recommendation adherence score (0-8). | Quality of life  • Average WCRF/AICR-2007 adherence score: 4.81 ± 1.04 out of 8 points  • Women had higher adherence scores than men  Higher adherence seen in non-smokers, older individuals, and those not receiving chemotherapy.  • Factors like years since diagnosis, tumor characteristics, and treatment were evenly distributed across adherence tertiles.  • Adherence to BMI recommendation: 34%, physical activity: 75%, mean dietary adherence: 3.48 ± 0.87 out of 6 points  • Varied adherence rates to specific dietary recommendations (e.g., sugary drinks, plant-based foods, meat products)  Men generally had higher HRQoL scores in various domains.  • Younger age, no comorbidities, and non-smoking associated with higher HRQoL scores  Highest WCRF/AICR-2007 adherence linked to better physical and role functioning, lower fatigue.  • Multivariable models showed higher adherence associated with improved physical, role, social functioning, and lower fatigue  Highest adherence tertile correlated with higher scores in emotional and cognitive functioning, global health status  • Increased adherence consistently associated with better functioning and global health, and less fatigue  • Adherence to physical activity linked to improved functioning and reduced fatigue.  • Obesity associated with lower physical functioning  • Dietary adherence not significantly associated with different functioning scales, global health, or fatigue. |
| **The World Cancer Research Fund (WCRF)/American Institute for Cancer Research (AICR) 2018 recommendations** | | | | | | |
| Bennett et al. (2024) | Ireland | Mean age 65.5±9.3  Male 82.5%  Female 17.5%  Esophageal 80%  Gastric 20%  n=40 | One 131-item European Prospective Investigation of Cancer (EPIC) Food Frequency Questionnaire (FFQ) | Based on the World Cancer Research Fund/American Institute for Cancer Research (WCRF/AICR) 2018 recommendations (WCRF/ACIR, 2018) | WCRF/AICR recommendations included in this study are consumption of fruit, vegetables, fibre, % total energy of adapted ultra-processed foods, adapted red and process meat, total sugar-sweetened drinks and alcohol.  Two components of the WCRF/AICR score were adapted: ultra-processed foods (aUPFs) and weekly consumption of red and processed meat.  To calculate the WCRF/AICR adherence score, each recommendation was assigned a score of 1 point for complete adherence, 0·5 points for moderate adherence and 0 points for non-adherence.  Scores for the 7 indicators were summed as the recommendation adherence score (0-7). | Malnutrition risk, gastrointestinal (GI) symptoms and adherence to cancer prevention recommendations  • 50% of participants had a BMI in the healthy range.  • Over 25% were at risk of malnutrition.  • Consumption of meat and meat products exceeded recommended intake, while intake of fruits, vegetables, and fiber was below recommendations, with no significant differences observed between the groups.  • The average WCRF/AICR-2018 score was 3.6±1.1, reflecting adherence to 3.6 out of 7 cancer prevention guidelines, with no significant differences observed between subgroups.  • Mild to moderate gastrointestinal discomfort was reported, with no significant variation in symptoms between the groups. |
| Kenkhuis et al. (2021) | The Netherlands | Mean age 69.7 ± 8.7  Male 62 %,  Female 38 %  Colorectal 100%,  Colon 53.3%,  Rectosigmoid and rectum 46.7%  n=150 | One dietary record of 7 consecutive days (quantitative data on food and beverage consumption) | As above | WCRF/AICR recommendations included in this study are consumption of fruit, vegetables, fibre, % total energy of adapted ultra-processed foods, red and process meat, total sugar-sweetened drinks and alcohol.  Predefined cut-off values of the WCRF/AICR recommendations adherence categories were used  This study did not operationalize following recommendations on:  BMI, physical activity and breast feeding.  To calculate the WCRF/AICR adherence score, each recommendation was assigned a score of 1 point for complete adherence, 0·5 points for moderate adherence and 0 points for non-adherence. A higher score thus indicated a higher adherence to recommendations  Scores for the 5 indicators were summed as the recommendation adherence score (0-5). | HRQoL, fatigue and neuropathy • Increased vegetable intake (per 50 g) was linked to enhanced overall QoL, improved physical functioning, and reduced levels of fatigue.  • Elevated intake of fruits and vegetables (per 100 g) was associated with improved physical functioning, while higher consumption of energy-dense foods (per 100 kJ/100 g) was correlated with diminished physical functioning.  • No discernible associations were observed between adherence to dietary recommendations and neuropathy.  • Higher consumption of energy-dense foods was linked to poorer physical functioning and increased fatigue.  • Non-alcoholic individuals exhibited significantly lower levels of physical, role, and social functioning, a lower EORTC summary score, and higher levels of fatigue compared to moderate alcohol drinkers.  • These findings suggest that following specific dietary recommendations outlined by WCRF/AICR-2018 is connected with enhanced HRQoL and reduced fatigue in colorectal cancer survivor |
| Koole et al. (2020) | The Netherlands | Mean age 65.4 ±7.7  Male 76%,  Female 24%  Colorectal 100%  n=100 | One 7-day dietary record was an adapted version of the EnCoRe FFQ  , covering 253 unique food products (2 weeks later follow up and measured intake in the preceding month) | As above | WCRF/AICR recommendations included in this study are consumption of fruit & vegetables, fibre, fast foods (energy density of diet), red and process meat, sugar-sweetened drinks and alcohol.  Predefined cut-off values of the WCRF/AICR recommendations adherence categories were used  This study did not operationalize following recommendations on:  BMI, physical activity and breast feeding  To calculate the WCRF/AICR adherence score, each recommendation was assigned a score of 1 point for complete adherence, 0·5 points for moderate adherence and 0 points for non-adherence. A higher score thus indicated a higher adherence to recommendations  Scores for the 6 indicators were summed as the recommendation adherence score (0-6). | • The average total daily energy intake for men was recorded at 2,193 kcal, while for women, it was 1,733 kcal.  • The FFQ reported higher figures, with men at 2,253 kcal and women at 1,941 kcal.  • When compared to the dietary record, the FFQ showed absolute intakes of macronutrients, including protein, total fat, and carbohydrates, to be approximately 6% to 7% higher.  • Alcohol intake was notably lower, showing a reduction of 22% in the FFQ compared to the dietary record  • Discrepancies of around 5% were observed in the intake of various food categories, including bread, cheese, cereals and cereal products, vegetables, meat, meat products, and poultry, as well as fats, oils, and savory sauces when comparing the FFQ and the dietary record.  • The most significant differences between the two methods were noted in savory bread spreads, soy products, and vegetarian products, along with mixed dishes, all of which were estimated to be more than 40% lower according to the FFQ in comparison to the dietary record.  • WCRF/AICR-2018 were assessed for the total population and separately for men and women using both the FFQ and dietary record methods.  • The Spearman correlation between the total scores obtained from the two methods was 0.53. The FFQ yielded a median score of 2.5 (ranging from 0.5 to 4.0), while the dietary record resulted in a median score of 2.0 (ranging from 0.5 to 5.5).  • Women scored a median of 3.0 points using both methods, whereas men scored a median of 2.5 points with the FFQ and a median of 2.0 points with the dietary record.  • The performance of the FFQ was relatively poorer (correlations < 0.50) for certain micronutrients, including folic acid, riboflavin, calcium, and dietary folate equivalents, as well as for specific food groups like legumes, nuts, seeds, snacks, and fats, oils, and savory sauces.  • The kappa coefficients were relatively low (< 0.40) for nutrients such as protein, total fat, minerals like calcium and magnesium, and vitamins like riboflavin and vitamin B-6, approximately 70% to 80% of participants were still classified in the exact or adjacent quintile and few participants (4%) were categorized in the opposite quintile.  • Despite lower correlations, the FFQ demonstrated reasonable agreement in quintile classification for these nutrients among the study population.  • The correlation of 0.53 between the total WCRF/ACIR 2018 scores obtained from both the FFQ and dietary record was considered moderate to good.  • The FFQ yielded higher scores compared to the dietary record. Kappa coefficients for the distribution of scoring 0, 0.5, or 1 point were below 0.40 for individual recommendations on energy density, fruit and vegetables, dietary fiber, and red and processed meat.  • Correlation coefficients between individual recommendations were all less than 0.50.  • When ranking subjects based on their adherence scores, both methods demonstrated moderate to good agreement, while the exact number of points scored was less comparable between the two methods |
| Song et al. (2021) | USA | Mean age 68.5 ± 8.5  Male 42.5%, Female 57.5%  Colorectal 100%  n=1,491 | Validated semiquantitative FFQs were reported in 1980, 1984 and every 4 years since 1986 | As above | WCRF/AICR recommendations included in this study for men are: fruits and vegetables, dietary fiber, whole grains, nuts, and legumes, refined grains and processed foods high in fat and sugar, red and processed meat, sugar-sweetened beverages, and alcohol.  Predefined cut-off values of the WCRF/AICR recommendations adherence categories were used.  This study did not operationalize following recommendations on:  PA, BMI and breast feeding  To calculate the WCRF/AICR adherence score, each recommendation was assigned a score of 1 point for complete adherence, 0·5 points for moderate adherence and 0 points for non-adherence.  The final WCRF/AICR diet score was calculated by averaging the seven diet component scores and ranged 0-1.  Scores for the 7 indicators were summed as the recommendation adherence score (0-7). A higher score thus indicated a higher adherence to recommendations. | Survival in colorectal patients  • The percentage of deaths was higher in individuals assigned to the lowest category of the WCRF/AICR-2018 score (those whose lifestyle was least)  • Over a median follow-up period of 7.92 years, there were a total of 641 deaths, including 179 from colorectal cancer.  • Patients in the highest quartile of the post-diagnostic WCRF/AICR-2018 score, encompassing factors such as diet, body mass index (BMI), and physical activity, exhibited a 24% lower risk of colorectal cancer–specific mortality and a 37% lower risk of overall mortality compared to those in the lowest quartile  • When BMI was excluded from the lifestyle score due to potential disease-related weight loss, more robust inverse associations were observed for both colorectal cancer–specific and overall mortality in the same comparison.  • The post-diagnostic WCRF/AICR-2018 diet score did not show a statistically significant association with either colorectal cancer–specific or overall mortality. |
| van Zutphen et al. (2019) | The Netherlands | Mean age  65 ± 9,  Male 63%, Female 37%  Colon 67%, Rectum 33%  n=1,072 | Three validated semi-quantitative food frequency questionnaire (FFQ) with 204 items at baseline and 6 months and 2 years after CRC diagnosis. | Based on the World Cancer Research Fund/American Institute of Cancer Research (WCRF/ AICR) 2018 recommendations (WCRF/ACIR, 2018), and the Division of Human Nutrition and Health Wageningen University Research, the Netherlands | WCRF/AICR recommendations included in this study are healthy weight, physical activity consumption of fruit & vegetables, fibre, fast foods (energy density of diet), red and process meat, sugar-sweetened drinks and alcohol.  Predefined cut-off values of the WCRF/AICR recommendations adherence categories were used  This study did not operationalize recommendations for breast feeding .  To calculate the WCRF/AICR adherence score, each recommendation was assigned a score of 1 point for complete adherence, 0·5 points for moderate adherence and 0 points for non-adherence.  Scores for the 7 indicators were summed as the recommendation adherence score (0-7). A higher score thus indicated a higher adherence to recommendations. | • Following colorectal cancer (CRC) diagnosis, adherence to dietary recommendations was limited, with lowest adherence for limiting red and processed meat (8%) and highest for limiting ultra-processed foods (33%).  • A majority (90%) adhered to the physical activity recommendation at CRC diagnosis.  • 38% maintained a healthy BMI, and 24% had a healthy waist circumference. Improvements over two years included reduced sugary drink consumption (-45 g/day) and red/processed meat intake (-62 g/week).  • Deviations included decreased fiber intake (1 g/day) and increased BMI (0.4 kg/m2) and waist circumference (2 cm).  • Participants generally did not significantly change fruit/vegetable intake, ultra-processed foods, or smoking habits over two years.Initial reductions in alcohol intake and physical activity levels were not statistically significant after two years.  • Overall lifestyle improvement was marginal, with no significant differences based on demographics or clinical characteristics.  • Participants living without a partner showed better two-year lifestyle improvement.  • Nearly all participants (92%) changed adherence to at least one of the seven WCRF/AICR lifestyle recommendations within two years after CRC diagnosis.  • 70% demonstrated improvement, with 51% making simultaneous changes improving adherence to one recommendation while decreasing adherence to another.  • 20% solely improved adherence, while 24% solely decreased adherence.  • Changes did not follow a distinct pattern, and effect sizes were consistent even when excluding participants diagnosed with recurrence within two years. |
| **The American Cancer Society guidelines diet scores (ACS)** | | | | | | |
| McCullough et al. (2016) | USA | Mean age at diagnosis 70.7 ±7.2  Female 100%  Breast 100%  n=4,452 | One modified food frequency questionnaire (FFQ) with the baseline survey in 1992 with 68 items    Two modified Harvard FFQ with follow-up surveys (1999 and 2003) with 152-items | The diet score was developed by McCullough et al. (2016) as part of an overall lifestyle score used to evaluate the association of the American Cancer Society Nutrition and Physical Activity Guidelines for Cancer  Prevention (Kushi et al., 2012) | Three key food-based recommendations:  -Consume 5+ servings of a variety of fruits and vegetables’’ (one point for consuming >=5 servings/day and 0, 1, 2 points for tertiles of variety, summed), ‘  -Choose whole grains in preference to processed, refined grains’’ (quartiles of percent grains that are whole),  -Limit consumption of red and processed meats’’ (quartiles of total red and processed meat, reverse-scored)  Each key food-based recommendation contributing 0–3 points, with a score of 3 reflecting optimal adherence.  Components scores were then summed up to a maximum score 9.  diet scores [highest (6–9) versus lowest (0–2) scores | Risk of death from breast cancer, CVD, or other causes • Pre- and postdiagnostic diet pattern scores aligned with the ACS no association with breast cancer-specific mortality, CVD mortality, or overall mortality.  • A two-point increase in the postdiagnostic diet score was linked to a 12% lower risk of non-CVD, non-breast cancer causes of death.  • The common causes in this category included other cancers, respiratory illness, and Alzheimer's disease.  • Higher consumption of red and processed meat after diagnosis was the only component of the ACS score independently associated with non-breast cancer causes of mortality.  • Specifically, the lowest versus highest quartile of red and processed meat intake after diagnosis was associated with a statistically significant 48%, 43%, and 36% lower risk of CVD, non-breast, non-CVD causes of death, and total mortality, respectively.  • No significant association was observed between fruit and vegetable consumption and breast cancer survival, regardless of whether the assessment was made before or after diagnosis.  • While the ACS guidelines do not explicitly recommend moderating fat or fiber intake for breast cancer survivors, these constituents tracked in the expected directions with the ACS score.  • Red and processed meat consumption emerged as the only score component linked to mortality from causes other than breast cancer in this study.  • The study found no differences in results based on tumor estrogen receptor (ER) or progesterone receptor (PR) status or local/regional tumor stage. However, stability in associations among women with ER-negative tumors was less evident, likely due to smaller numbers. |

Note. This table summarizes studies evaluating dietary intake and adherence to diet quality indices or dietary guidelines in relation to health outcomes among cancer survivors. Data includes details about the country, population demographics (mean age, sex distribution, cancer type), dietary intake assessment methods, evidence base for diet quality index development, scoring methodology, interpretations of the diet quality indices used and findings from the studies. DGA: Dietary Guidelines for Americans, HEI: Healthy Eating Index, FFQ: Food frequency questionnaire, Qol: Quality of Life, DHD-II: Diet History Questionaire II, QFFQ: Quantitative food frequency questionnaire,

RDA: Recommended Dietary Allowances.

**Table S5.** Details of dietary components used in Diet Quality Indices for older adult cancer survivors

|  | **HEI-2005** (Demark-Wahnefried et al., 2012; Mosher et al., 2009; Pelser et al., 2014)  **HEI-2010**  (Gopalakrishna et al., 2018; Sun et al., 2018; Wang et al., 2021)  **HEI-2015** (Krok-Schoen et al., 2021; Pisegna et al., 2021; Schmalenberger et al., 2022) | **RHEI**  (Miller et al., 2008; Snyder et al., 2009) | **BHEI- R** (Mazzutti et al., 2021) | **DQI-R**  (Clutter Snyder et al., 2007; Demark-Wahnefried et al., 2006; Demark-Wahnefried et al., 2003) | **MDS**  (Bauer et al., 2018) | **DHD-I** (Breedveld-Peters et al., 2018) | **9-item index** (Klassen et al., 2018) | **WCRF/AICR-2007** (Breedveld-Peters et al., 2018; Inoue-Choi et al., 2014; Inoue-Choi et al., 2013a; Inoue-Choi et al., 2013b; van Veen et al., 2019)  **WCRF/AICR-2018** (Bennett et al., 2024; Kenkhuis et al., 2021; Koole et al., 2020; Song et al., 2021; van Zutphen et al., 2019) | **ACS** (McCullough et al., 2016) | **Use** |
| --- | --- | --- | --- | --- | --- | --- | --- | --- | --- | --- |
| **Adequacy:** |  |  |  |  |  |  |  |  |  |  |
| **Total Grains** | HEI-2005 (Guenther et al., 2008) √  DGA (U.S. Department of Health and Human Services and U.S. Department of Agriculture, 2005) recommended  ≥3.0 oz equiv. per 1,000 kcal | RHEI (Guenther et al., 2006) √  DGA (U.S. Department of Health and Human Services and U.S. Department of Agriculture, 2005) recommended  ≥3.0 oz equiv. per 1,000 kcal | BHEI-R (Previdelli et al., 2011) √  DGB (MInistery of Health Brazil/Secretariat of Health Care Primary Health Care Department, 2006) recommended  ≥2 portions equiv. per 4184 kJ |  | MDS (Bauer et al., 2018) √  Grains above median intake, i.e. using a cut off score derived from the observed cohort |  |  |  | ACS Diet score (McCullough et al., 2016) √  Recommended to choose whole grains in preference to processed, refined grains | 5/12 |
| **Whole grains** | HEI-2005 (Guenther et al., 2008) √  DGA (U.S. Department of Health and Human Services and U.S. Department of Agriculture, 2005) recommended  ≥1.5 oz equiv. per 1,000 kcal  HEI-2010 (Guenther et al., 2013) √  DGA (McGuire, 2011) recommended  ≥1.5 oz equiv. per 1,000 kcal  HEI-2015 (Krebs-Smith et al., 2018) √  DGA (US Department of Health and Human Services and US Department of Agriculture, 2015) recommended  ≥1.5 oz equiv. per 1,000 kcal | RHEI (Guenther et al., 2006) √  DGA (U.S. Department of Health and Human Services and U.S. Department of Agriculture, 2005) recommended  ≥1.5 oz equiv. per 1,000 kcal | BHEI-R (Previdelli et al., 2011) √  DGB (MInistery of Health Brazil/Secretariat of Health Care Primary Health Care Department, 2006) recommended  ≥1 portion equiv. per 4184 kJ | DQI-R (Haines et al., 1999)√  recommended ≥9 servings/d for men or≥ 6 servings/d for women |  |  |  |  |  | 6/12 |
| **Total Vegetables** | HEI-2005 (Guenther et al., 2008) √  DGA (U.S. Department of Health and Human Services and U.S. Department of Agriculture, 2005) recommended  ≥1.1 cup equiv. per 1,000 kcal  HEI-2010 (Guenther et al., 2013) √  DGA (McGuire, 2011) recommended  ≥1.1 cup equiv. per 1,000 kcal  HEI-2015 (Krebs-Smith et al., 2018) √  DGA (US Department of Health and Human Services and US Department of Agriculture, 2015) recommended  ≥1.1 cup equiv. per 1,000 kcal | RHEI (Guenther et al., 2006) √  DGA (U.S. Department of Health and Human Services and U.S. Department of Agriculture, 2005) recommended  ≥1.1 cup equiv. per 1,000 kcal | BHEI-R (Previdelli et al., 2011) √  DGB (MInistery of Health Brazil/Secretariat of Health Care Primary Health Care Department, 2006) recommended  ≥1 portion equiv. per 4184 kJ | DQI-R (Haines et al., 1999)√  recommended ≥4 servings/d for men or ≥3 servings/d for women | MDS (Bauer et al., 2018) √ above the median, i.e, using a cut off score derived from the observed cohort | DHD-Index (van Lee et al., 2012) √  DGHD (Health Council of the Netherlands, 2006) recommend  150–200 g/d |  |  |  | 8/12 |
| **Dark green leafy**  **and orange vegetables and Legumes** | HEI-2005 (Guenther et al., 2008) √  DGA (U.S. Department of Health and Human Services and U.S. Department of Agriculture, 2005) recommended  ≥0.4 cup equiv. per 1,000 kcal | RHEI (Guenther et al., 2006) √  DGA (U.S. Department of Health and Human Services and U.S. Department of Agriculture, 2005) recommended  ≥0.4 cup equiv. per 1,000 kcal | BHEI-R (Previdelli et al., 2011) √  DGB (MInistery of Health Brazil/Secretariat of Health Care Primary Health Care Department, 2006) recommended  ≥0.5 portion equiv. per 4184 kJ |  |  |  |  |  |  | 3/12 |
| **Greens and Beans** | HEI-2010 (Guenther et al., 2013) √  DGA (McGuire, 2011) recommended  ≥0.2 cup equiv. per 1,000 kcal  HEI-2015 (Krebs-Smith et al., 2018) √  DGA (US Department of Health and Human Services and US Department of Agriculture, 2015) recommended  ≥0.2 cup equiv. per 1,000 kcal |  |  |  |  |  |  |  |  | 2/12 |
| **Total Fruit** | HEI-2005 (Guenther et al., 2008) √  DGA (U.S. Department of Health and Human Services and U.S. Department of Agriculture, 2005) recommended  ≥0.8 cup equiv. per 1,000 kcal  HEI-2010 (Guenther et al., 2013) √  DGA(McGuire, 2011) recommended  ≥0.8 cup equiv. per 1,000 kcal  HEI-2015 (Krebs-Smith et al., 2018) √  DGA (US Department of Health and Human Services and US Department of Agriculture, 2015) recommended  ≥0.8 cup equiv. per 1,000 kcal | RHEI (Guenther et al., 2006) √ DGA (U.S. Department of Health and Human Services and U.S. Department of Agriculture, 2005) recommended  ≥0.8 cup equiv. per 1,000 kcal | BHEI-R (Previdelli et al., 2011) √  DGB (MInistery of Health Brazil/Secretariat of Health Care Primary Health Care Department, 2006)  recommended  ≥1 portion equiv. per 4184 kJ | DQI-R (Haines et al., 1999)√  recommended ≥3 servings/d for men or ≥2 servings/d for women | MDS (Bauer et al., 2018) √  Above median intake, i.e., using a cut off score derived from the observed cohort | DHD-Index (van Lee et al., 2012) √  DGHD (Health Council of the Netherlands, 2006)  recommend 200 g/d |  |  |  | 8/12 |
| **Whole fruit** | HEI-2005 (Guenther et al., 2008) √  DGA (U.S. Department of Health and Human Services and U.S. Department of Agriculture, 2005) recommended  ≥0.4 cup equiv. per 1,000 kcal  HEI-2010 (Guenther et al., 2013) √  DGA (McGuire, 2011) recommended  ≥0.4 cup equiv. per 1,000 kcal  HEI-2015 (Krebs-Smith et al., 2018) √  DGA (US Department of Health and Human Services and US Department of Agriculture, 2015) recommended  ≥0.4 cup equiv. per 1,000 kcal | RHEI (Guenther et al., 2006) √  DGA (U.S. Department of Health and Human Services and U.S. Department of Agriculture, 2005) recommended  ≥0.4 cup equiv. per 1,000 kcal | BHEI-R (Previdelli et al., 2011) √  DGB (MInistery of Health Brazil/Secretariat of Health Care Primary Health Care Department, 2006) recommended  ≥0.5 portion equiv. per 4184 kJ |  |  |  |  |  |  | 5/9 |
| **Fruit and**  **Vegetables**  **combined** |  |  |  |  |  |  | The 9-item index (Klassen et al., 2018) √  recommended 5+ servings | WCRF/AICR 2007 (WCRF/ACIR, 2007) √  Recommended intake ≥400 g of a variety of non-starchy fruits and vegetables every day  WCRF/AICR 2018 (WCRF/ACIR, 2018)√  Recommended intake ≥400 g of a variety of non-starchy fruits and vegetables every day | ACS Diet score (McCullough et al., 2016) √  recommended 5+ servings of a variety of fruits and vegetables | 4/12 |
| **Beans, Pulses,**  **legumes** |  |  |  |  | MDS (Bauer et al., 2018) √  Above median intake, i.e., using a cut off score derived from the observed cohort |  |  |  |  | 1/12 |
| **Nuts** |  |  |  |  | MDS (Bauer et al., 2018) √  Above median intake, i.e., using a cut off score derived from the observed cohort |  |  |  |  | 1/12 |
| **Dairy, Milk** | HEI-2005 (Guenther et al., 2008) √  DGA (U.S. Department of Health and Human Services and U.S. Department of Agriculture, 2005) recommended  ≥1.3 cup equiv. per 1,000 kcal  HEI-2010 (Guenther et al., 2013) √  DGA (McGuire, 2011) recommended  ≥1.3 cup equiv. per 1,000 kcal  HEI-2015 (Krebs-Smith et al., 2018) √  DGA (US Department of Health and Human Services and US Department of Agriculture, 2015)  recommended  ≥1.3 cup equiv. per 1,000 kcal | RHEI (Guenther et al., 2006) √  DGA (U.S. Department of Health and Human Services and U.S. Department of Agriculture, 2005) recommended  ≥1.3 cup equiv. per 1,000 kcal | BHEI-R (Previdelli et al., 2011) √  DGB (MInistery of Health Brazil/Secretariat of Health Care Primary Health Care Department, 2006)  recommended  ≥1.5 portions equiv. per 4184 kJ |  |  |  |  |  |  | 5/12 |
| **Total protein foods** | HEI-2010 (Guenther et al., 2013) √  DGA (McGuire, 2011) recommended  ≥2.5 oz equiv. per 1,000 kcal  HEI-2015 (Krebs-Smith et al., 2018) √  DGA (US Department of Health and Human Services and US Department of Agriculture, 2015) recommended  ≥2.5 oz equiv. per 1,000 kcal |  |  |  |  |  |  |  |  | 2/12 |
| **Fish** |  |  |  |  | MDS (Bauer et al., 2018) √  Above median intake, i.e., using a cut off score derived from the observed cohort | DHD-Index (van Lee et al., 2012) √  DGHD (Health Council of the Netherlands, 2006)  recommend 2 serving/week |  |  |  | 2/12 |
| **Seafood and Plant Protein** | HEI-2010 (Guenther et al., 2013) √  DGA (McGuire, 2011) recommended  ≥0.8 oz equiv. per 1,000 kcal  HEI-2015 (Krebs-Smith et al., 2018) √  DGA (US Department of Health and Human Services and US Department of Agriculture, 2015) recommended  ≥0.8 oz equiv. per 1,000 kcal |  |  |  |  |  |  |  |  | 2/12 |
| **Meats and Beans** | HEI-2005 (Guenther et al., 2008) √  DGA (U.S. Department of Health and Human Services and U.S. Department of Agriculture, 2005) recommended  ≥2.5 oz equiv. per 1,000 kcal | RHEI (Guenther et al., 2006) √  DGA (U.S. Department of Health and Human Services and U.S. Department of Agriculture, 2005) recommended  ≥2.5 oz equiv. per 1,000 kcal |  |  |  |  |  |  |  | 2/12 |
| **Meat, Eggs and Beans** |  |  | BHEI-R (Previdelli et al., 2011) √  DGB (MInistery of Health Brazil/Secretariat of Health Care Primary Health Care Department, 2006) recommended  ≥1 portion equiv. per 4184 kJ |  |  |  |  |  |  | 1/12 |
| **Fibre** |  |  |  |  |  | DHD-Index (van Lee et al., 2012) √  DGHD (Health Council of the Netherlands, 2006)  recommend 30–40 g/d | The 9-item index (Klassen et al., 2018) √  recommended  ≥25 g for women, ≥28 g for men | WCRF/AICR 2007 (WCRF/ACIR, 2007) √  Recommended intake ≥25 g/day of fibre from food sources  WCRF/AICR 2018 (WCRF/ACIR, 2018) √  Recommended intake ≥30 g/day of fibre from food sources |  | 4/12 |
| **Calcium** |  |  |  | DQI-R (Haines et al., 1999)√  800–1000 mg/d for men or ≥1200 mg/d for women |  |  | The 9-item index(Klassen et al., 2018) √recommended  ≥1000 mg if age 31-50 years, ≥1200 mg if age 51+ years |  |  | 2/12 |
| **Iron** |  |  |  | DQI-R (Haines et al., 1999)√  recommended 10 mg/d for both men and women |  |  |  |  |  | 1/12 |
| **Vitamin D** |  |  |  |  |  |  | The 9-item index (Klassen et al., 2018) √  recommended ≥15μg, ≥20 if age 70+ years |  |  | 1/12 |
| **Fatty Acids**  **(PUFA + MUFA)/SFA** | HEI-2010 (Guenther et al., 2013) √  DGA (McGuire, 2011) recommended  (PUFAs + MUFAs)/SFAs ≥2.5  HEI-2015 (Krebs-Smith et al., 2018)√  DGA (US Department of Health and Human Services and US Department of Agriculture, 2015) recommended  (PUFAs + MUFAs)/SFAs ≥2.5 |  |  |  |  |  |  |  |  | 2/12 |
| **Ratio of PUFA: SFA** |  |  |  |  | MDS (Bauer et al., 2018) √ above median intake, i.e., using a cut off score derived from the observed cohort |  |  |  |  | 1/12 |
| **Oils (including mono- and polyunsaturated fats, oilseeds and fish fat)** | HEI-2005 (Guenther et al., 2008) √  DGA (U.S. Department of Health and Human Services and U.S. Department of Agriculture, 2005) recommended  ≥12 grams equiv. per 1,000 kcal |  | BHEI-R (Previdelli et al., 2011) √  DGB (MInistery of Health Brazil/Secretariat of Health Care Primary Health Care Department, 2006) recommended  ≥0.5 portion equiv. per 4184 kJ |  |  |  |  |  |  | 2/12 |
| **Moderation:** |  |  |  |  |  |  |  |  |  |  |
| **Red/processed meat** |  |  |  |  |  |  |  | WCRF/AICR 2007 (WCRF/ACIR, 2007) √  Recommended to limit intake of red meat and avoid processed meat  Red and processed meat <500 g/w and processed meat intake <3 g/d  WCRF/AICR 2018 (WCRF/ACIR, 2018) √  Recommended to limit intake of red meat and avoid processed meat, red meat ≤500g/week (≤18 oz) and processed meat <21g/week (<0.75 oz) | ACS Diet score (McCullough et al., 2016) √  Recommended to limit consumption of red and processed meats | 3/12 |
| **Refined Grains** | HEI-2010 (Guenther et al., 2013) √  DGA (McGuire, 2011) recommended  ≤1.8 oz equiv. per 1,000 kcal  HEI-2015 (Krebs-Smith et al., 2018) √  DGA (US Department of Health and Human Services and US Department of Agriculture, 2015) recommended  ≤1.8 oz equiv. per 1,000 kcal |  |  |  |  |  |  |  |  | 2/12 |
| **Added sugar/sugary foods and drinks** |  |  |  |  |  | DHD-Index (van Lee et al., 2012) √  DGHD (Health Council of the Netherlands, 2006)  recommend limit the consumption of foods and beverages that contain easily fermentable sugars and drinks |  |  |  | 1/12 |
| **Sugar-sweetened beverages and fruit drinks** |  |  |  |  |  | DHD-Index (van Lee et al., 2012) √  DGHD (Health Council of the Netherlands, 2006)  Recommend  up to a maximum of 100 g of fruit juices that naturally contain folate and vitamin C |  | WCRF/AICR 2007 (WCRF/ACIR, 2007) √  Recommend to avoid sugary drinks (total sugar-sweetened drinks 0 g/day)  WCRF/AICR 2018 (WCRF/ACIR, 2018)√  Recommended to avoid sugary drinks (total sugar-sweetened drinks 0 g/day) |  | 3/12 |
| **Percent of total calories from ultra-processed foods (aUPFs** |  |  |  |  |  |  |  | WCRF/AICR 2018 (WCRF/ACIR, 2018) √  Recommended to limit consumption of “fast foods” and other processed foods high in fat, starches, or sugars  (referencing to tertile 1 of analysed cohort) |  | 1/12 |
| **Total fat** |  |  |  | DQI-R (Haines et al., 1999)√  recommended  <30% of total calories |  |  |  |  |  | 1/12 |
| **Saturated Fat** | HEI-2005 (Guenther et al., 2008) √  DGA (U.S. Department of Health and Human Services and U.S. Department of Agriculture, 2005) recommended  ≤7% of energy  HEI-2015 (Krebs-Smith et al., 2018) √  DGA (US Department of Health and Human Services and US Department of Agriculture, 2015) recommended  ≤8% of energy | RHEI (Guenther et al., 2006) √  DGA (U.S. Department of Health and Human Services and U.S. Department of Agriculture, 2005)  recommended  ≤7% of energy | BHEI-R (Previdelli et al., 2011) √  DGB (MInistery of Health Brazil/Secretariat of Health Care Primary Health Care Department, 2006)  recommended  7% of TEV | DQI-R (Haines et al., 1999)√  recommended  <10% of total calories | MDS (Bauer et al., 2018) √  below median intake, i.e., using a cut off score derived from the observed cohort | DHD-Index (van Lee et al., 2012) √  DGHD (Health Council of the Netherlands, 2006)  Recommended <10 % of energy intake | The 9-item index (Klassen et al., 2018) √  ≤10% of total calories |  |  | 8/12 |
| **Trans fat** |  |  |  |  |  | DHD-Index (van Lee et al., 2012) √  DGHD (Health Council of the Netherlands, 2006)  recommend <1 % of energy intake |  |  |  | 1/12 |
| **Cholesterol** |  |  |  | DQI-R (Haines et al., 1999)√  recommended  <300 mg/d |  |  | The 9-item index (Klassen et al., 2018) √  recommended limit intake <300mg |  |  | 2/12 |
| **Salt or Sodium** | HEI-2005 (Guenther et al., 2008) √  DGA (U.S. Department of Health and Human Services and U.S. Department of Agriculture, 2005) recommended  ≤0.7 gram per 1,000 kcal  HEI-2010 (Guenther et al., 2013)√  DGA (McGuire, 2011) recommended  ≤1.1 gram per 1,000 kcal  HEI-2015 (Krebs-Smith et al., 2018)√  DGA (US Department of Health and Human Services and US Department of Agriculture, 2015) recommended  ≤1.1 gram per 1,000 kcal | RHEI (Guenther et al., 2006) √  DGA (U.S. Department of Health and Human Services and U.S. Department of Agriculture, 2005) recommended  ≤0.7 gram per 1,000 kcal | BHEI-R (Previdelli et al., 2011)√  DGB (MInistery of Health Brazil/Secretariat of Health Care Primary Health Care Department, 2006)  recommended  ≤0.7 gram per 4184 kJ |  |  | DHD-Index (van Lee et al., 2012) √  DGHD (Health Council of the Netherlands, 2006)  recommended limit consumption of table salt to 6 g/d | The 9-item index (Klassen et al., 2018) √  ≤2300 mg, or ≤1500 for age 50 years and older, African Americans, or those with specific chronic diseases | WCRF/AICR 2007 (WCRF/ACIR, 2007)√  Recommended to limit  consumption of salty foods and foods processed with salt. ≤1,500 mg/d |  | 8/12 |
| **Alcohol** |  |  |  |  | MDS (Bauer et al., 2018) √ recommended to limit intake between 10 and 50 g/d | DHD-Index (van Lee et al., 2012) √  DGHD (Health Council of the Netherlands, 2006)  recommend limited to two Dutch units (20 g ethanol) a day for males and one for females | The 9-item index (Klassen et al., 2018) √  recommended limit intake ≤1 drink daily for women, ≤2 for men | WCRF/AICR 2007 (WCRF/ACIR, 2007) √  Recommended to limit alcoholic drinks., if alcoholic drinks are consumed, Ethanol intake ≤20 g/d (♂)  Ethanol intake ≤10 g/d (♀)  WCRF/AICR 2018 (WCRF/ACIR, 2018) √  recommended to limit alcoholic drinks., Total ethanol 0g/day |  | 5/12 |
| **Calories from Solid Fats, Alcoholic Beverages, and Added Sugars (SoFAAS)** | HEI-2005 (Guenther et al., 2008)√  DGA (U.S. Department of Health and Human Services and U.S. Department of Agriculture, 2005) recommended  ≤20% of energy  HEI-2010 (Guenther et al., 2013) √  DGA (McGuire, 2011) recommended  ≤19% of energy | RHEI (Guenther et al., 2006) √  DGA (U.S. Department of Health and Human Services and U.S. Department of Agriculture, 2005) recommended  ≤20% of energy | BHEI-R (Previdelli et al., 2011) √  DGB (MInistery of Health Brazil/Secretariat of Health Care Primary Health Care Department, 2006) recommended  ≤10% of TEV |  |  |  |  |  |  | 4/12 |
| **Total Calories** |  |  |  |  |  |  | The 9-item index (Klassen et al., 2018) √  recommended total calories (1600-2200, based on age, gender, and sedentary/  active lifestyle |  |  | 1/12 |
| **Diet Diversity** |  |  |  | Includes 23 food subgroups to describe dietary diversity and all foods were assigned to one of these subgroups and scored |  |  |  |  |  | 1/12 |
| **Dietary moderation** |  |  |  | Comprised of Added sugar, alcohol, discretionary fat, and sodium, each contributes 0-2.5 points for total of 0-10. |  |  |  |  |  | 1/12 |
| **Dietary Supplements** |  |  |  |  |  |  |  | WCRF/AICR 2007 (WCRF/ACIR, 2007) √  Recommended to meet nutritional needs through diet alone; dietary supplements are not recommended for cancer prevention |  | 1/12 |
| **Energy density considered** | HEI-2005 (Guenther et al., 2008) √  HEI-2010 (Guenther et al., 2013) √  HEI-2015 (Krebs-Smith et al., 2018) √  Considers scoring  per 1,000 kcal | Considers scoring  per 1,000 kcal | Considers scoring  per 1,000 kcal | Considers scoring  per 1,000 kcal, for fruits, vegetables and grains |  | Considers scoring  per 4.2MJ per day for fibre, saturated fat and trans fats | Considers scoring  per per day for saturated fat | WCRF/AICR 2007 (WCRF/ACIR, 2007) √  Recommended to limit consumption of energy-dense food and drinks, ED ≤125 kcal/100g/d |  | 1/12 |

Note. This table summarizes the components and scoring criteria of different dietary quality indices including HEI: Healthy Eating Index, HEI-2005: Healthy Eating Index 2005, HEI-2010: Healthy Eating Index 2015, HEI-2015: Healthy Eating Index 2015, BHEI-R: Brazil Healthy Eating Index Revised, RHEI: Revised Healthy Eating Index, WCRF/AICR-2007: World Cancer Research Fund/American Institute for Cancer Research 2007, WCRF/AICR-2018: World Cancer Research Fund/American Institute for Cancer Research 2018, DQI-R: Diet Quality Index Revised, Mediterranean Diet Score: MDS, DHDI: Dutch Healthy Diet Index DGA: Dietary Guidelines for Americans, DGA: Dietary Guidelines for Brazilian Population, The American Cancer Society guidelines diet scores (ACS) , TEV: Total Energy Value

**Table S1:** Preferred Reporting Items for Systematic reviews and Meta-Analyses extension for Scoping Reviews (PRISMA-ScR) Checklist (2018)

| **SECTION** | **ITEM** | **PRISMA-ScR CHECKLIST ITEM** | **REPORTED ON PAGE #** |
| --- | --- | --- | --- |
| **TITLE** | | | |
| Title | 1 | Identify the report as a scoping review. | 1 |
| **ABSTRACT** | | | |
| Structured summary | 2 | Provide a structured summary that includes (as applicable): background, objectives, eligibility criteria, sources of evidence, charting methods, results, and conclusions that relate to the review questions and objectives. | 1 |
| **INTRODUCTION** | | | |
| Rationale | 3 | Describe the rationale for the review in the context of what is already known. Explain why the review questions/objectives lend themselves to a scoping review approach. | 2 |
| Objectives | 4 | Provide an explicit statement of the questions and objectives being addressed with reference to their key elements (e.g., population or participants, concepts, and context) or other relevant key elements used to conceptualize the review questions and/or objectives. | 2 |
| **METHODS** | | | |
| Protocol and registration | 5 | Indicate whether a review protocol exists; state if and where it can be accessed (e.g., a Web address); and if available, provide registration information, including the registration number. | Available upon request |
| Eligibility criteria | 6 | Specify characteristics of the sources of evidence used as eligibility criteria (e.g., years considered, language, and publication status), and provide a rationale. | 2-5 |
| Information sources* | 7 | Describe all information sources in the search (e.g., databases with dates of coverage and contact with authors to identify additional sources), as well as the date the most recent search was executed. | 2-5 |
| Search | 8 | Present the full electronic search strategy for at least 1 database, including any limits used, such that it could be repeated. | Table S4 |
| Selection of sources of evidence† | 9 | State the process for selecting sources of evidence (i.e., screening and eligibility) included in the scoping review. | 2 |
| Data charting process‡ | 10 | Describe the methods of charting data from the included sources of evidence (e.g., calibrated forms or forms that have been tested by the team before their use, and whether data charting was done independently or in duplicate) and any processes for obtaining and confirming data from investigators. | 2-5  Table S1  Table S2 |
| Data items | 11 | List and define all variables for which data were sought and any assumptions and simplifications made. | 2-5  Table S1  Table S2 |
| Critical appraisal of individual sources of evidence§ | 12 | If done, provide a rationale for conducting a critical appraisal of included sources of evidence; describe the methods used and how this information was used in any data synthesis (if appropriate). | N/A |
| Synthesis of results | 13 | Describe the methods of handling and summarizing the data that were charted. | 2-5 |
| **RESULTS** | | | |
| Selection of sources of evidence | 14 | Give numbers of sources of evidence screened, assessed for eligibility, and included in the review, with reasons for exclusions at each stage, ideally using a flow diagram. | 2-3, Fig 1, S3 Table 3 |
| Characteristics of sources of evidence | 15 | For each source of evidence, present characteristics for which data were charted and provide the citations. | 2-5  Table S1  Table S2  Table S3 |
| Critical appraisal within sources of evidence | 16 | If done, present data on critical appraisal of included sources of evidence (see item 12). | N/A |
| Results of individual sources of evidence | 17 | For each included source of evidence, present the relevant data that were charted that relate to the review questions and objectives. | 2-5  Table S1  Table S2 |
| Synthesis of results | 18 | Summarize and/or present the charting results as they relate to the review questions and objectives. | 2-5 |
| **DISCUSSION** | | | |
| Summary of evidence | 19 | Summarize the main results (including an overview of concepts, themes, and types of evidence available), link to the review questions and objectives, and consider the relevance to key groups. | 5-9 |
| Limitations | 20 | Discuss the limitations of the scoping review process. | 9 |
| Conclusions | 21 | Provide a general interpretation of the results with respect to the review questions and objectives, as well as potential implications and/or next steps. | 9 |
| **FUNDING** | | | |
| Funding | 22 | Describe sources of funding for the included sources of evidence, as well as sources of funding for the scoping review. Describe the role of the funders of the scoping review. | 10 |

**Table S2:** Diet quality indices in older adult cancer survivors: a scoping review

Search Strategy – Medline, from inception to 12^th^ November 2024

| **#** | **Searches** | **Results** |
| --- | --- | --- |
| 1 | exp Neoplasms/ | 3886657 |
| 2 | (Cancer* or neoplas* or tumo?r* or Malignan* or carcinoma* or metasta* or leuk?em* or lymphoma* or myeloma* or sarcoma*).tw,kf. | 4455250 |
| 3 | 1 or 2 | 5250946 |
| 4 | survivors/ | 30786 |
| 5 | (survivor* or remission).tw,kf. | 273529 |
| 6 | Remission Induction/ | 44603 |
| 7 | (("patient outcome*" or "patient's outcome*" or "symptom free" or asymptomatic) adj3 ("12 month*" or "3 year*" or "5 year*")).tw,kf. | 741 |
| 8 | 4 or 5 or 6 or 7 | 301934 |
| 9 | 3 and 8 | 138354 |
| 10 | Cancer Survivors/ | 9129 |
| 11 | 9 or 10 | 138982 |
| 12 | Diet, Mediterranean/ or Diet/ or Diet, Healthy/ or Diet, Western/ or Diet, Sodium-Restricted/ or Dietary Approaches To Stop Hypertension/ | 204979 |
| 13 | ((food* or diet*) adj5 (quality or index or indices or pattern* or asses* or improv* or scale or adherence)).tw,kf. | 119663 |
| 14 | ("dietary indices" or "Healthy Eating Index" or "Mediterranean Dietary Score" or "Healthy Food Diversity index" or Assessment* or "Diet Quality Index – International" or DASH).tw,kf. | 1420924 |
| 15 | 12 or 13 or 14 | 1679931 |
| 16 | aged/ or "aged, 80 and over"/ | 3466270 |
| 17 | ((older adj1 adult*) or elderly or (Aged adj3 ("over 65" or ">65" or "65" or "70" or "80"))).tw,kf. | 458073 |
| 18 | 16 or 17 | 3590825 |
| 19 | 11 and 15 and 18 | 3158 |
| 20 | "Systematic Review"/ or "Review"/ | 3335554 |
| 21 | review.ti. or review.pt. | 3512257 |
| 22 | 20 or 21 | 3533667 |
| 23 | 19 not 22 | 2958 |
| 24 | limit 23 to english language | 2828 |

*Translated above strategy for other databases: CINAHL; Scopus, COCHRANE; Web of Science*

**Table S3.** List of excluded articles and reason

| **Article**  **number** | **Reference** | **Reason for exclusion after full text screening** |
| --- | --- | --- |
| 1 | Skiba, M.B., Jacobs, E.T., Crane, T.E., Kopp, L.M., Thomson, C.A., 2022. Relationship Between Individual Health Beliefs and Fruit and Vegetable Intake and Physical Activity Among Cancer Survivors: Results from the Health Information National Trends Survey. J Adolesc Young Adult Oncol 11, 259-267. | No diet quality indices used |
| 2 | Winger, J.G., Mosher, C.E., Rand, K.L., Morey, M.C., Snyder, D.C., Demark-Wahnefried, W., 2014. Diet and exercise intervention adherence and health-related outcomes among older long-term breast, prostate, and colorectal cancer survivors. Ann Behav Med 48, 235-245. | No diet quality indices used |
| 3 | Tan, Z., Meng, Y., Li, L., Wu, Y., Liu, C., Dong, W., Chen, C., 2023. Association of Dietary Fiber, Composite Dietary Antioxidant Index and Risk of Death in Tumor Survivors: National Health and Nutrition Examination Survey 2001-2018. Nutrients 15. | Does not have food groups - mostly micronutrients |
| 4 | Hawkins, N.A., Berkowitz, Z., Rodriguez, J.L., 2015. Awareness of Dietary and Alcohol Guidelines Among Colorectal Cancer Survivors. Am J Prev Med 49, S509-517. | No diet quality indices used |
| 5 | Meyerhardt, J.A., Niedzwiecki, D., Hollis, D., Saltz, L.B., Hu, F.B., Mayer, R.J., Nelson, H., Whittom, R., Hantel, A., Thomas, J., Fuchs, C.S., 2007. Association of dietary patterns with cancer recurrence and survival in patients with stage III colon cancer. Jama 298, 754-764. | No diet quality indices used |
| 6 | Springfield, S., Odoms-Young, A., Tussing-Humphreys, L.M., Freels, S., Stolley, M.R., 2019. A Step toward Understanding Diet Quality in Urban African-American Breast Cancer Survivors: A Cross-sectional Analysis of Baseline Data from the Moving Forward Study. Nutr Cancer 71, 61-76. | Wrong population |
| 7 | Maskarinec, G., Murphy, S., Shumay, D.M., Kakai, H., 2001. Dietary changes among cancer survivors. Eur J Cancer Care (Engl) 10, 12-20. | No diet quality indices used |
| 8 | Fonseca, J., Santos, C.A., Brito, J., 2016. Malnutrition and Clinical Outcome of 234 Head and Neck Cancer Patients who Underwent Percutaneous Endoscopic Gastrostomy. Nutr Cancer 68, 589-597. | No diet quality indices used |
| 9 | Jo, S.R., Joh, J.Y., Jeong, J.R., Kim, S., Kim, Y.P., 2015. Health Behaviors of Korean Gastric Cancer Survivors with Hypertension: A Propensity Analysis of KNHANES III-V (2005-2012). PLoS One 10, e0126927.. | No diet quality indices used |
| 10 | Berry, N.M., Miller, M.D., Woodman, R.J., Coveney, J., Dollman, J., Mackenzie, C.R., Koczwara, B., 2014. Differences in chronic conditions and lifestyle behaviour between people with a history of cancer and matched controls. Med J Aust 201, 96-100. | Wrong population  Non-cancer participants >25% of study population |
| 11 | Schlesinger, S., Walter, J., Hampe, J., von Schönfels, W., Hinz, S., Küchler, T., Jacobs, G., Schafmayer, C., Nöthlings, U., 2014. Lifestyle factors and health-related quality of life in colorectal cancer survivors. Cancer Causes Control 25, 99-110. | No diet quality indices used |
| 12 | Tat, D., Kenfield, S.A., Cowan, J.E., Broering, J.M., Carroll, P.R., Van Blarigan, E.L., Chan, J.M., 2018. Milk and other dairy foods in relation to prostate cancer recurrence: Data from the cancer of the prostate strategic urologic research endeavor (CaPSURE™). Prostate 78, 32-39. | No diet quality indices used |
| 13 | Heitz, A.E., Baumgartner, R.N., Baumgartner, K.B., Boone, S.D., 2018. Healthy lifestyle impact on breast cancer-specific and all-cause mortality. Breast Cancer Res Treat 167, 171-181. | No diet quality indices used |
| 14 | Mosher, C.E., Lipkus, I., Sloane, R., Snyder, D.C., Lobach, D.F., Demark-Wahnefried, W., 2013. Long-term outcomes of the FRESH START trial: exploring the role of self-efficacy in cancer survivors' maintenance of dietary practices and physical activity. Psychooncology 22, 876-885. | No diet quality indices used |
| 15 | Reedy, J., Haines, P.S., Campbell, M.K., 2005. The influence of health behavior clusters on dietary change. Prev Med 41, 268-275. | No diet quality indices used |
| 16 | Chan, J.M., Van Blarigan, E.L., Langlais, C.S., Zhao, S., Ramsdill, J.W., Daniel, K., Macaire, G., Wang, E., Paich, K., Kessler, E.R., Beer, T.M., Lyons, K.S., Broering, J.M., Carroll, P.R., Kenfield, S.A., Winters-Stone, K.M., 2020. Feasibility and Acceptability of a Remotely Delivered, Web-Based Behavioral Intervention for Men With Prostate Cancer: Four-Arm Randomized Controlled Pilot Trial. J Med Internet Res 22, e19238. | No diet quality indices used |
| 17 | Ergas, I.J., Cespedes Feliciano, E.M., Bradshaw, P.T., Roh, J.M., Kwan, M.L., Cadenhead, J., Santiago-Torres, M., Troeschel, A.N., Laraia, B., Madsen, K., Kushi, L.H., 2021. Diet Quality and Breast Cancer Recurrence and Survival: The Pathways Study. JNCI Cancer Spectr 5. | Wrong population |
| 18 | Madlensky, L., Natarajan, L., Flatt, S.W., Faerber, S., Newman, V.A., Pierce, J.P., 2008. Timing of dietary change in response to a telephone counseling intervention: evidence from the WHEL study. Health Psychol 27, 539-547. | No diet quality indices used |
| 19 | Reeves, M.M., Terranova, C.O., Erickson, J.M., Job, J.R., Brookes, D.S., McCarthy, N., Hickman, I.J., Lawler, S.P., Fjeldsoe, B.S., Healy, G.N., Winkler, E.A., Janda, M., Veerman, J.L., Ware, R.S., Prins, J.B., Vos, T., Demark-Wahnefried, W., Eakin, E.G., 2016. Living well after breast cancer randomized controlled trial protocol: evaluating a telephone-delivered weight loss intervention versus usual care in women following treatment for breast cancer. BMC Cancer 16, 830. | No diet quality indices used |
| 20 | Palacios, C., Daniel, C.R., Tirado-Gómez, M., Gonzalez-Mercado, V., Vallejo, L., Lozada, J., Ortiz, A., Hughes, D.C., Basen-Engquist, K., 2017. Dietary Patterns in Puerto Rican and Mexican-American Breast Cancer Survivors: A Pilot Study. J Immigr Minor Health 19, 341-348. | Wrong population |
| 21 | Lei, Y.Y., Ho, S.C., Cheng, A., Kwok, C., Lee, C.I., Cheung, K.L., Lee, R., Loong, H.H.F., He, Y.Q., Yeo, W., 2018. Adherence to the World Cancer Research Fund/American Institute for Cancer Research Guideline Is Associated With Better Health-Related Quality of Life Among Chinese Patients With Breast Cancer. J Natl Compr Canc Netw 16, 275-285. | Wrong population |
| 22 | Kim, N.H., Song, S., Jung, S.Y., Lee, E., Kim, Z., Moon, H.G., Noh, D.Y., Lee, J.E., 2018. Dietary pattern and health-related quality of life among breast cancer survivors. BMC Womens Health 18, 65. | No diet quality indices used |
| 23 | Thomson, C.A., Crane, T.E., Miller, A., Garcia, D.O., Basen-Engquist, K., Alberts, D.S., 2016. A randomized trial of diet and physical activity in women treated for stage II-IV ovarian cancer: Rationale and design of the Lifestyle Intervention for Ovarian Cancer Enhanced Survival (LIVES): An NRG Oncology/Gynecologic Oncology Group (GOG-225) Study. Contemp Clin Trials 49, 181-189. | No diet quality indices used |
| 24 | Alfano, C.M., Day, J.M., Katz, M.L., Herndon, J.E., 2nd, Bittoni, M.A., Oliveri, J.M., Donohue, K., Paskett, E.D., 2009. Exercise and dietary change after diagnosis and cancer-related symptoms in long-term survivors of breast cancer: CALGB 79804. Psychooncology 18, 128-133. | No diet quality indices used |
| 25 | Garrett, K., Okuyama, S., Jones, W., Barnes, D., Tran, Z., Spencer, L., Lewis, K., Maroni, P., Chesney, M., Marcus, A., 2013. Bridging the transition from cancer patient to survivor: pilot study results of the Cancer Survivor Telephone Education and Personal Support (C-STEPS) program. Patient Educ Couns 92, 266-272. | No diet quality indices used |
| 26 | Gould Rothberg, B.E., Bulloch, K.J., Fine, J.A., Barnhill, R.L., Berwick, M., 2014. Red meat and fruit intake is prognostic among patients with localized cutaneous melanomas more than 1mm thick. Cancer Epidemiol 38, 599-607. | No diet quality indices used |
| 27 | Kwan, M.L., Kushi, L.H., Danforth, K.N., Roh, J.M., Ergas, I.J., Lee, V.S., Cannavale, K.L., Harrison, T.N., Contreras, R., Loo, R.K., Aaronson, D.S., Quesenberry, C.P., Tritchler, D., Ghai, N.R., Quinn, V.P., Ambrosone, C.B., Zhang, Y., Tang, L., 2019. The Be-Well Study: a prospective cohort study of lifestyle and genetic factors to reduce the risk of recurrence and progression of non-muscle-invasive bladder cancer. Cancer Causes Control 30, 187-193. | No diet quality indices used |
| 28 | Beesley, V.L., Eakin, E.G., Janda, M., Battistutta, D., 2008. Gynecological cancer survivors' health behaviors and their associations with quality of life. Cancer Causes Control 19, 775-782. | No diet quality indices used |
| 29 | Kaur, H., Fernández, J.R., Locher, J.L., Demark-Wahnefried, W., 2022. Rural and Urban Differences in Vegetable and Fruit Consumption Among Older Cancer Survivors in the Deep South: An Exploratory Cross-Sectional Study. J Acad Nutr Diet 122, 1717-1724.e1714. | No diet quality indices used |
| 30 | Gray, M.S., Judd, S.E., Sloane, R., Snyder, D.C., Miller, P.E., Demark-Wahnefried, W., 2019. Rural-urban differences in health behaviors and outcomes among older, overweight, long-term cancer survivors in the RENEW randomized control trial. Cancer Causes Control 30, 301-309. | No diet quality indices used |
| 31 | Gu, Q., Dummer, T.B.J., Spinelli, J.J., Murphy, R.A., 2019. Diet Quality among Cancer Survivors and Participants without Cancer: A Population-Based, Cross-Sectional Study in the Atlantic Partnership for Tomorrow's Health Project. Nutrients 11. | Wrong population  Non-cancer participants >25% of study population |
| 32 | Maino Vieytes, C.A., Mondul, A.M., Crowder, S.L., Zarins, K.R., Edwards, C.G., Davis, E.C., Wolf, G.T., Rozek, L.S., Arthur, A.E., On Behalf Of The University Of Michigan, H., Neck Spore, P., 2021. Pretreatment Adherence to a Priori-Defined Dietary Patterns Is Associated with Decreased Nutrition Impact Symptom Burden in Head and Neck Cancer Survivors. Nutrients 13. | Wrong population |
| 33 | Gordon, L.G., Patrao, T., Kularatna, S., Hawkes, A.L., 2015. A telephone-delivered multiple health behaviour change intervention for colorectal cancer survivors: making the case for cost-effective healthcare. Eur J Cancer Care (Engl) 24, 854-861. | No diet quality indices used |
| 34 | Blanchard, C.M., Courneya, K.S., Stein, K., 2008. Cancer survivors' adherence to lifestyle behavior recommendations and associations with health-related quality of life: results from the American Cancer Society's SCS-II. J Clin Oncol 26, 2198-2204. | No diet quality indices used |
| 35 | Caan, B., Sternfeld, B., Gunderson, E., Coates, A., Quesenberry, C., Slattery, M.L., 2005. Life After Cancer Epidemiology (LACE) Study: a cohort of early stage breast cancer survivors (United States). Cancer Causes Control 16, 545-556. | No diet quality indices used |
| 36 | Vance, V., Campbell, S., McCargar, L., Mourtzakis, M., Hanning, R., 2014. Dietary changes and food intake in the first year after breast cancer treatment. Appl Physiol Nutr Metab 39, 707-714. | No diet quality indices used |
| 37 | Demark-Wahnefried, W., Peterson, B., McBride, C., Lipkus, I., Clipp, E., 2000. Current health behaviors and readiness to pursue life-style changes among men and women diagnosed with early stage prostate and breast carcinomas. Cancer 88, 674-684. | No diet quality indices used |
| 38 | Pierce, J.P., Newman, V.A., Flatt, S.W., Faerber, S., Rock, C.L., Natarajan, L., Caan, B.J., Gold, E.B., Hollenbach, K.A., Wasserman, L., Jones, L., Ritenbaugh, C., Stefanick, M.L., Thomson, C.A., Kealey, S., 2004. Telephone counseling intervention increases intakes of micronutrient- and phytochemical-rich vegetables, fruit and fiber in breast cancer survivors. J Nutr 134, 452-458. | No diet quality indices used |
| 39 | Lewis, J.E., Soler-Vilá, H., Clark, P.E., Kresty, L.A., Allen, G.O., Hu, J.J., 2009. Intake of plant foods and associated nutrients in prostate cancer risk. Nutr Cancer 61, 216-224. | No diet quality indices used |
| 40 | Shahril, M.R., Zakarai, N.S., Appannah, G., Nurnazahiah, A., Mohamed, H.J., Ahmad, A., Lua, P.L., Fenech, M., 2021. ‘Energy-Dense, High-SFA and Low-Fiber’ Dietary Pattern Lowered Adiponectin but Not Leptin Concentration of Breast Cancer Survivors, Nutrients. | No diet quality indices used |
| 41 | Zhang, F.F., Liu, S., John, E.M., Must, A., Demark-Wahnefried, W., 2015. Diet quality of cancer survivors and noncancer individuals: Results from a national survey. Cancer 121, 4212-4221. | Wrong population |
| 42 | Pekmezi, D., Fontaine, K., Rogers, L.Q., Pisu, M., Martin, M.Y., Schoenberger-Godwin, Y.M., Oster, R.A., Kenzik, K., Ivankova, N.V., Demark-Wahnefried, W., 2022. Adapting MultiPLe behavior Interventions that eFfectively Improve (AMPLIFI) cancer survivor health: program project protocols for remote lifestyle intervention and assessment in 3 inter-related randomized controlled trials among survivors of obesity-related cancers. BMC Cancer 22, 471. | No diet quality scores listed |
| 43 | Anderson, C., Sandler, D.P., Weinberg, C.R., Houck, K., Chunduri, M., Hodgson, M.E., Sabatino, S.A., White, M.C., Rodriguez, J.L., Nichols, H.B., 2017. Age- and treatment-related associations with health behavior change among breast cancer survivors. Breast 33, 1-7. | No diet quality scores listed |
| 44 | Dennis Parker, E.A., Sheppard, V.B., Adams-Campbell, L., 2013. Compliance With National Nutrition Recommendations Among Breast Cancer Survivors in “Stepping Stone”. Integrative Cancer Therapies 13, 114-120. | No diet quality scores listed |
| 45 | Rim, C.H., Ahn, S.J., Kim, J.H., Yoon, W.S., Chun, M., Yang, D.S., Lee, J.H., Kim, K., Kong, M., Kim, S., Kim, J., Park, K.R., Shin, Y.J., Ma, S.Y., Jeong, B.K., Kim, S.S., Kim, Y.B., Lee, D.S., Cha, J., 2019. Questionnaire study of the dietary habits of breast cancer survivors and their relationship to quality of life (KROG 14-09). Eur J Cancer Care (Engl) 28, e12961. | Wrong population |
| 46 | Potter, J.L., Collins, C.E., Brown, L.J., Hure, A.J., 2014. Diet quality of Australian breast cancer survivors: a cross-sectional analysis from the Australian Longitudinal Study on Women's Health. J Hum Nutr Diet 27, 569-576. | Wrong population |
| 47 | Bergengren, O., Enblad, A.P., Garmo, H., Bratt, O., Holmberg, L., Johansson, E., Bill-Axelson, A., 2020. Changes in lifestyle among prostate cancer survivors: A nationwide population-based study. Psychooncology 29, 1713-1719. | No diet quality indices used |
| 48 | Miller, P.E., Morey, M.C., Hartman, T.J., Snyder, D.C., Sloane, R., Cohen, H.J., Demark-Wahnefried, W., 2012. Dietary patterns differ between urban and rural older, long-term survivors of breast, prostate, and colorectal cancer and are associated with body mass index. J Acad Nutr Diet 112, 824-831, 831.e821. | No diet quality indices used |
| 49 | Wu, T., Seaver, P., Lemus, H., Hollenbach, K., Wang, E., Pierce, J.P., 2019. Associations between Dietary Acid Load and Biomarkers of Inflammation and Hyperglycemia in Breast Cancer Survivors. Nutrients 11. | Wrong population |
| 50 | Dennett, A.M., Hirko, K.A., Porter, K.J., Loh, K.P., Liao, Y., Yang, L., Arem, H., Sukumar, J.S., Salerno, E.A., 2023. Embedding lifestyle interventions into cancer care: has telehealth narrowed the equity gap? J Natl Cancer Inst Monogr 2023, 133-139. | No diet quality indices used |
| 51 | Wang, Z., McLoone, P., Morrison, D.S., 2015. Diet, exercise, obesity, smoking and alcohol consumption in cancer survivors and the general population: a comparative study of 16 282 individuals. Br J Cancer 112, 572-575. | Wrong population  Non-cancer participants >25% of study population |
| 52 | Zick, S.M., Colacino, J., Cornellier, M., Khabir, T., Surnow, K., Djuric, Z., 2017. Fatigue reduction diet in breast cancer survivors: a pilot randomized clinical trial. Breast Cancer Res Treat 161, 299-310. | No diet quality indices used |
| 53 | Song, M., Wu, K., Meyerhardt, J.A., Ogino, S., Wang, M., Fuchs, C.S., Giovannucci, E.L., Chan, A.T., 2018. Fiber Intake and Survival After Colorectal Cancer Diagnosis. JAMA Oncol 4, 71-79. | No diet quality indices used |
| 54 | Morey, M.C., Snyder, D.C., Sloane, R., Cohen, H.J., Peterson, B., Hartman, T.J., Miller, P., Mitchell, D.C., Demark-Wahnefried, W., 2009. Effects of home-based diet and exercise on functional outcomes among older, overweight long-term cancer survivors: RENEW: a randomized controlled trial. Jama 301, 1883-1891. | No diet quality indices used |
| 55 | Park, S.H., Knobf, M.T., Kerstetter, J., Jeon, S., 2019. Adherence to American Cancer Society Guidelines on Nutrition and Physical Activity in Female Cancer Survivors: Results From a Randomized Controlled Trial (Yale Fitness Intervention Trial). Cancer Nurs 42, 242-250. | Wrong population |
| 56 | Saquib, N., Rock, C.L., Natarajan, L., Flatt, S.W., Newman, V.A., Thomson, C.A., Caan, B.J., Pierce, J.P., 2009. Does a healthy diet help weight management among overweight and obese people? Health Educ Behav 36, 518-531. | No diet quality indices used |
| 57 | Zuniga, K.E., Parma, D.L., Muñoz, E., Spaniol, M., Wargovich, M., Ramirez, A.G., 2019. Dietary intervention among breast cancer survivors increased adherence to a Mediterranean-style, anti-inflammatory dietary pattern: the Rx for Better Breast Health Randomized Controlled Trial. Breast Cancer Res Treat 173, 145-154. | Wrong population |
| 58 | Ristevsk, E., Trinh, T., Vo, N., Byrne, A., Jamieson, P., Greenall, A., Barber, G., Roman, A., Schmidt, U., 2020. I.CAN: health coaching provides tailored nutrition and physical activity guidance to people diagnosed with cancer in a rural region in West Gippsland, Australia. J Cancer Surviv 14, 48-52. | No diet quality indices used |
| 59 | McMenamin, E., Gottschalk, A.B., Pucci, D.A., Jacobs, L.A., 2023. Health behaviors among head and neck cancer survivors. J Health Popul Nutr 42, 48. | No diet quality indices used |
| 60 | Tan, S.Y., Wong, H.Y., Vardy, J.L., 2021. Do cancer survivors change their diet after cancer diagnosis? Support Care Cancer 29, 6921-6927. | No diet quality indices used |
| 61 | Panagiotakos, D.B., Dimakopoulou, K., Katsouyanni, K., Bellander, T., Grau, M., Koenig, W., Lanki, T., Pistelli, R., Schneider, A., Peters, A., 2009. Mediterranean diet and inflammatory response in myocardial infarction survivors. Int J Epidemiol 38, 856-866. | Wrong population |
| 62 | Cha, R., Murray, M.J., Thompson, J., Wall, C.R., Hill, A., Hulme-Moir, M., Merrie, A., Findlay, M.P., 2012. Dietary patterns and information needs of colorectal cancer patients post-surgery in Auckland. N Z Med J 125, 38-46. | No diet quality indices used |
| 63 | Lee, M.K., Park, S.Y., Choi, G.S., 2019. Facilitators and Barriers to Adoption of a Healthy Diet in Survivors of Colorectal Cancer. J Nurs Scholarsh 51, 509-517. | No diet quality indices used |
| 64 | van den Berg, M.G., Rütten, H., Rasmussen-Conrad, E.L., Knuijt, S., Takes, R.P., van Herpen, C.M., Wanten, G.J., Kaanders, J.H., Merkx, M.A., 2014. Nutritional status, food intake, and dysphagia in long-term survivors with head and neck cancer treated with chemoradiotherapy: a cross-sectional study. Head Neck 36, 60-65. | No diet quality indices used |
| 65 | St George, S.M., Noriega Esquives, B., Agosto, Y., Kobayashi, M., Leite, R., Vanegas, D., Perez, A.T., Calfa, C., Schlumbrecht, M., Slingerland, J., Penedo, F.J., 2020. Development of a multigenerational digital lifestyle intervention for women cancer survivors and their families. Psychooncology 29, 182-194. | No diet quality indices used |
| 66 | Park, B., Lee, J., Kim, J., 2018. Imbalanced Nutrient Intake in Cancer Survivors from the Examination from the Nationwide Health Examination Center-Based Cohort. Nutrients 10. | Wrong population |
| 67 | Slade, A.N., 2019. Prostate cancer and subsequent nutritional outcomes: the role of diagnosis and treatment. J Cancer Surviv 13, 171-179. | No diet quality indices used |
| 68 | Anderson, A.S., Caswell, S., Wells, M., Steele, R.J., Macaskill, S., 2010. "It makes you feel so full of life" LiveWell, a feasibility study of a personalised lifestyle programme for colorectal cancer survivors. Support Care Cancer 18, 409-415. | No diet quality indices used |
| 69 | Kanera, I.M., Bolman, C.A., Mesters, I., Willems, R.A., Beaulen, A.A., Lechner, L., 2016. Prevalence and correlates of healthy lifestyle behaviors among early cancer survivors. BMC Cancer 16, 4. | No diet quality indices used |
| 70 | Ho, M., Ho, J.W.C., Fong, D.Y.T., Lee, C.F., Macfarlane, D.J., Cerin, E., Lee, A.M., Leung, S., Chan, W.Y.Y., Leung, I.P.F., Lam, S.H.S., Chu, N., Taylor, A.J., Cheng, K.K., 2020. Effects of dietary and physical activity interventions on generic and cancer-specific health-related quality of life, anxiety, and depression in colorectal cancer survivors: a randomized controlled trial. J Cancer Surviv 14, 424-433. | No diet quality indices used |
| 71 | George, S.M., Bernstein, L., Smith, A.W., Neuhouser, M.L., Baumgartner, K.B., Baumgartner, R.N., Ballard-Barbash, R., 2014. Central adiposity after breast cancer diagnosis is related to mortality in the Health, Eating, Activity, and Lifestyle study. Breast Cancer Res Treat 146, 647-655. | Wrong population |
| 72 | Hammerlid, E., Wirblad, B., Sandin, C., Mercke, C., Edström, S., Kaasa, S., Sullivan, M., Westin, T., 1998. Malnutrition and food intake in relation to quality of life in head and neck cancer patients. Head Neck 20, 540-548. | No diet quality indices used |
| 73 | Wang, F., Cai, H., Gu, K., Shi, L., Yu, D., Zhang, M., Zheng, W., Zheng, Y., Bao, P., Shu, X.O., 2020. Adherence to Dietary Recommendations among Long-Term Breast Cancer Survivors and Cancer Outcome Associations. Cancer Epidemiol Biomarkers Prev 29, 386-395. | Wrong population |
| 74 | Christifano, D.N., Fazzino, T.L., Sullivan, D.K., Befort, C.A., 2016. Diet Quality of Breast Cancer Survivors after a Six-Month Weight Management Intervention: Improvements and Association with Weight Loss. Nutr Cancer 68, 1301-1308. | Wrong population |
| 75 | Hawkes, A.L., Chambers, S.K., Pakenham, K.I., Patrao, T.A., Baade, P.D., Lynch, B.M., Aitken, J.F., Meng, X., Courneya, K.S., 2013. Effects of a telephone-delivered multiple health behavior change intervention (CanChange) on health and behavioral outcomes in survivors of colorectal cancer: a randomized controlled trial. J Clin Oncol 31, 2313-2321. | No diet quality indices used |
| 76 | Bourke, L., Thompson, G., Gibson, D.J., Daley, A., Crank, H., Adam, I., Shorthouse, A., Saxton, J., 2011. Pragmatic lifestyle intervention in patients recovering from colon cancer: a randomized controlled pilot study. Arch Phys Med Rehabil 92, 749-755. | No diet quality indices used |
| 77 | Van Blarigan, E.L., Fuchs, C.S., Niedzwiecki, D., Zhang, S., Saltz, L.B., Mayer, R.J., Mowat, R.B., Whittom, R., Hantel, A., Benson, A., Atienza, D., Messino, M., Kindler, H., Venook, A., Ogino, S., Giovannucci, E.L., Ng, K., Meyerhardt, J.A., 2018. Association of Survival With Adherence to the American Cancer Society Nutrition and Physical Activity Guidelines for Cancer Survivors After Colon Cancer Diagnosis: The CALGB 89803/Alliance Trial. JAMA Oncol 4, 783-790. | Wrong population |
| 78 | Crowder, S.L., Najam, N., Sarma, K.P., Fiese, B.H., Arthur, A.E., 2020. Head and Neck Cancer Survivors' Experiences with Chronic Nutrition Impact Symptom Burden after Radiation: A Qualitative Study. J Acad Nutr Diet 120, 1643-1653. | No diet quality indices used |
| 79 | Pierce, J.P., Natarajan, L., Caan, B.J., Parker, B.A., Greenberg, E.R., Flatt, S.W., Rock, C.L., Kealey, S., Al-Delaimy, W.K., Bardwell, W.A., Carlson, R.W., Emond, J.A., Faerber, S., Gold, E.B., Hajek, R.A., Hollenbach, K., Jones, L.A., Karanja, N., Madlensky, L., Marshall, J., Newman, V.A., Ritenbaugh, C., Thomson, C.A., Wasserman, L., Stefanick, M.L., 2007. Influence of a diet very high in vegetables, fruit, and fiber and low in fat on prognosis following treatment for breast cancer: the Women's Healthy Eating and Living (WHEL) randomized trial. Jama 298, 289-298. | No diet quality indices used |
| 80 | Bours, M.J., Beijer, S., Winkels, R.M., van Duijnhoven, F.J., Mols, F., Breedveld-Peters, J.J., Kampman, E., Weijenberg, M.P., van de Poll-Franse, L.V., 2015. Dietary changes and dietary supplement use, and underlying motives for these habits reported by colorectal cancer survivors of the Patient Reported Outcomes Following Initial Treatment and Long-Term Evaluation of Survivorship (PROFILES) registry. Br J Nutr 114, 286-296. | No diet quality indices used |
| 81 | Grimmett, C., Simon, A., Lawson, V., Wardle, J., 2015. Diet and physical activity intervention in colorectal cancer survivors: a feasibility study. Eur J Oncol Nurs 19, 1-6. | No diet quality indices used |
| 82 | Milliron, B.J., Vitolins, M.Z., Tooze, J.A., 2014. Usual dietary intake among female breast cancer survivors is not significantly different from women with no cancer history: results of the National Health and Nutrition Examination Survey, 2003-2006. J Acad Nutr Diet 114, 932-937. | No diet quality indices used |
| 83 | Springfield, S., Odoms-Young, A., Tussing-Humphreys, L., Freels, S., Stolley, M., 2019. Adherence to American Cancer Society and American Institute of Cancer Research dietary guidelines in overweight African American breast cancer survivors. J Cancer Surviv 13, 257-268. | Wrong population |
| 84 | Kwan, M.L., Weltzien, E., Kushi, L.H., Castillo, A., Slattery, M.L., Caan, B.J., 2009. Dietary patterns and breast cancer recurrence and survival among women with early-stage breast cancer. J Clin Oncol 27, 919-926. | No diet quality indices used |
| 85 | Wang, H.H., Chung, U.L., Tsay, S.L., Hsieh, P.C., Su, H.F., Lin, K.C., 2015. Development and preliminary testing of an instrument to measure healthiness of lifestyle among breast cancer survivors. Int J Nurs Pract 21, 923-932. | No diet quality indices used |
| 86 | Baguley, B.J., Skinner, T.L., Leveritt, M.D., Wright, O.R., 2017. Nutrition therapy with high intensity interval training to improve prostate cancer-related fatigue in men on androgen deprivation therapy: a study protocol. BMC Cancer 17, 1. | No diet quality scores used |
| 87 | Zheng, J., Tabung, F.K., Zhang, J., Liese, A.D., Shivappa, N., Ockene, J.K., Caan, B., Kroenke, C.H., Hébert, J.R., Steck, S.E., 2018. Association between Post-Cancer Diagnosis Dietary Inflammatory Potential and Mortality among Invasive Breast Cancer Survivors in the Women's Health Initiative. Cancer Epidemiol Biomarkers Prev 27, 454-463. | No diet quality indices used |
| 88 | Christensen, M.A., Smoak, P., Lisano, J.K., Hayward, R., Coronado, C., Kage, K., Shackelford, D., Stewart, L.K., 2019. Cardiorespiratory fitness, visceral fat, and body fat, but not dietary inflammatory index, are related to C-reactive protein in cancer survivors. Nutr Health 25, 195-202. | No diet quality indices used |
| 89 | Ratjen, I., Enderle, J., Burmeister, G., Koch, M., Nöthlings, U., Hampe, J., Lieb, W., 2021. Post-diagnostic reliance on plant-compared with animal-based foods and all-cause mortality in omnivorous long-term colorectal cancer survivors. Am J Clin Nutr 114, 441-449. | Plant-based diet index |
| 90 | Ratjen, I., Shivappa, N., Schafmayer, C., Burmeister, G., Nöthlings, U., Hampe, J., Hébert, J.R., Lieb, W., Schlesinger, S., 2019. Association between the dietary inflammatory index and all-cause mortality in colorectal cancer long-term survivors. Int J Cancer 144, 1292-1301. | No diet quality indices used |
| 91 | Lee, M.S., Huang, Y.C., Su, H.H., Lee, M.Z., Wahlqvist, M.L., 2011. A simple food quality index predicts mortality in elderly Taiwanese. J Nutr Health Aging 15, 815-821. | Wrong intervention |
| 92 | León-Salas, B., Zabaleta-del-Olmo, E., Llobera, J., Bolíbar-Ribas, B., López-Jiménez, T., Casajuana-Closas, M., Esteva, M., 2020. Health status, lifestyle habits, and perceived social support in long-term cancer survivors: a cross-sectional study. BMC Research Notes 13, 376. | Wrong population  Non-cancer participants >25% of study population |
| 93 | Tabung, F.K., Noonan, A., Lee, D.H., Song, M., Clinton, S.K., Spakowicz, D., Wu, K., Cheng, E., Meyerhardt, J.A., Fuchs, C.S., Giovannucci, E.L., 2020. Post-diagnosis dietary insulinemic potential and survival outcomes among colorectal cancer patients. BMC Cancer 20, 817. | No diet quality indices used |
| 94 | George, S.M., Ballard-Barbash, R., Shikany, J.M., Caan, B.J., Freudenheim, J.L., Kroenke, C.H., Vitolins, M.Z., Beresford, S.A., Neuhouser, M.L., 2014. Better postdiagnosis diet quality is associated with reduced risk of death among postmenopausal women with invasive breast cancer in the women's health initiative. Cancer Epidemiol Biomarkers Prev 23, 575-583. | Wrong population |
| 95 | Coa, K.I., Smith, K.C., Klassen, A.C., Thorpe, R.J., Jr., Caulfield, L.E., 2015. Exploring important influences on the healthfulness of prostate cancer survivors' diets. Qual Health Res 25, 857-870. | Wrong population |
| 96 | Crowder, S.L., Li, Z., Sarma, K.P., Arthur, A.E., 2021. Chronic Nutrition Impact Symptoms Are Associated with Decreased Functional Status, Quality of Life, and Diet Quality in a Pilot Study of Long-Term Post-Radiation Head and Neck Cancer Survivors. Nutrients 13. | Wrong population |
| 97 | Lee, E., Zhu, J., Velazquez, J., Bernardo, R., Garcia, J., Rovito, M., Hines, R.B., 2021. Evaluation of Diet Quality Among American Adult Cancer Survivors: Results From 2005-2016 National Health and Nutrition Examination Survey. J Acad Nutr Diet 121, 217-232. | Wrong population |
| 98 | Izano, M.A., Fung, T.T., Chiuve, S.S., Hu, F.B., Holmes, M.D., 2013. Are diet quality scores after breast cancer diagnosis associated with improved breast cancer survival? Nutr Cancer 65, 820-826. | Wrong population |
| 99 | Ollberding, N.J., Maskarinec, G., Wilkens, L.R., Henderson, B.E., Kolonel, L.N., 2011. Comparison of modifiable health behaviours between persons with and without cancer: the Multiethnic Cohort. Public Health Nutr 14, 1796-1804. | Wrong population |
| 100 | Romaguera, D., Ward, H., Wark, P.A., Vergnaud, A.C., Peeters, P.H., van Gils, C.H., Ferrari, P., Fedirko, V., Jenab, M., Boutron-Ruault, M.C., Dossus, L., Dartois, L., Hansen, C.P., Dahm, C.C., Buckland, G., Sánchez, M.J., Dorronsoro, M., Navarro, C., Barricarte, A., Key, T.J., Trichopoulou, A., Tsironis, C., Lagiou, P., Masala, G., Pala, V., Tumino, R., Vineis, P., Panico, S., Bueno-de-Mesquita, H.B., Siersema, P.D., Ohlsson, B., Jirström, K., Wennberg, M., Nilsson, L.M., Weiderpass, E., Kühn, T., Katzke, V., Khaw, K.T., Wareham, N.J., Tjønneland, A., Boeing, H., Quirós, J.R., Gunter, M.J., Riboli, E., Norat, T., 2015. Pre-diagnostic concordance with the WCRF/AICR guidelines and survival in European colorectal cancer patients: a cohort study. BMC Med 13, 107. | Wrong population |
| 101 | Winkels, R.M., van Lee, L., Beijer, S., Bours, M.J., van Duijnhoven, F.J., Geelen, A., Hoedjes, M., Mols, F., de Vries, J., Weijenberg, M.P., Kampman, E., 2016. Adherence to the World Cancer Research Fund/American Institute for Cancer Research lifestyle recommendations in colorectal cancer survivors: results of the PROFILES registry. Cancer Med 5, 2587-2595. | Wrong population |
| 102 | Park, J., Kim, J., Shin, D.W., Shin, J., Cho, B., Song, Y.M., 2023. Factors Associated with Dietary Habit Changes in Korean Stomach Cancer Survivors after Cancer Treatment. Nutrients 15. | Wrong population |
| 103 | Lewis, C.M., Wolf, W.A., Xun, P., Sandler, R.S., He, K., 2016. Racial differences in dietary changes and quality of life after a colorectal cancer diagnosis: a follow-up of the Study of Outcomes in Colorectal Cancer Survivors cohort. Am J Clin Nutr 103, 1523-1530. | Wrong population |
| 104 | Wayne, S.J., Baumgartner, K., Baumgartner, R.N., Bernstein, L., Bowen, D.J., Ballard-Barbash, R., 2006. Diet quality is directly associated with quality of life in breast cancer survivors. Breast Cancer Res Treat 96, 227-232. | Wrong population |
| 105 | Ryu, S.W., Son, Y.G., Lee, M.K., 2020. Motivators and barriers to adoption of a healthy diet by survivors of stomach cancer: A cross-sectional study. Eur J Oncol Nurs 44, 101703. | Wrong population |
| 106 | Natalucci, V., Ferri Marini, C., De Santi, M., Annibalini, G., Lucertini, F., Vallorani, L., Panico, A.R., Sisti, D., Saltarelli, R., Donati Zeppa, S., Agostini, D., Gervasi, M., Baldelli, G., Grassi, E., Nart, A., Rossato, M., Biancalana, V., Piccoli, G., Benelli, P., Villarini, A., Somaini, M., Catalano, V., Guarino, S., Pietrelli, A., Monaldi, S., Sarti, D., Barocci, S., Flori, M., Rocchi, M.B.L., Brandi, G., Stocchi, V., Emili, R., Barbieri, E., 2023. Movement and health beyond care, MoviS: study protocol for a randomized clinical trial on nutrition and exercise educational programs for breast cancer survivors. Trials 24, 134. | Wrong population |
| 107 | Chung, J., Kulkarni, G.S., Bender, J., Breau, R.H., Guttman, D., Maganti, M., Matthew, A., Morash, R., Papadakos, J., Jones, J.M., 2020. Modifiable lifestyle behaviours impact the health-related quality of life of bladder cancer survivors. BJU Int 125, 836-842. | No diet quality index used |
| 108 | Paunescu, A.C., Préau, M., Jacob, G., Pannard, M., Delrieu, L., Delpierre, C., Kvaskoff, M., 2023. Health behaviour changes in female cancer survivors: The Seintinelles study. Bull Cancer 110, 496-511. | Wrong population |
| 109 | Kleckner, A.S., Kleckner, I.R., Renn, C.L., Rosenblatt, P.Y., Ryan, A.S., Zhu, S., 2025. Dietary Composition, Meal Timing, and Cancer-Related Fatigue: Insights From the Women's Healthy Eating and Living Study. Cancer Nurs 48, 19-30. | Wrong population |
| 110 | Sremanakova, J., Sowerbutts, A.M., Todd, C., Cooke, R., Pearce, L., Leiberman, D., McLaughlin, J., Hill, J., Ashby, H., Ramesh, A., Burden, S., 2024. Healthy Eating and Active Lifestyle after Bowel Cancer (HEAL ABC)-feasibility randomised controlled trial. Eur J Clin Nutr 78, 1095-1104. | No full text |
| 111 | The development of dietary compliance intervention among colorectal cancer survivors based the social cognitive theory - intervention mapping ChiCTR2400083146, 2024;():2024 | No full text |
| 112 | Weinhold, K.R., Light, S., Bittoni, A.M., Zick, S., Orchard, T.S., 2023. A remote Whole Food Dietary Intervention to Reduce Fatigue and Improve Diet Quality in Lymphoma Survivors: Results of a Feasibility Pilot Study. Nutr Cancer 75, 937-947. | Wrong population |
| 113 | Porciello, G., Coluccia, S., Vitale, S., Palumbo, E., Luongo, A., Grimaldi, M., Pica, R., Prete, M., Calabrese, I., Cubisino, S., Montagnese, C., Falzone, L., Martinuzzo, V., Poletto, L., Rotondo, E., Di Gennaro, P., De Laurentiis, M., D'Aiuto, M., Rinaldo, M., Thomas, G., Messina, F., Catalano, F., Ferraù, F., Montesarchio, V., Serraino, D., Crispo, A., Libra, M., Celentano, E., Augustin, L.S.A., The, D.S.G., 2024. Baseline Association between Healthy Eating Index-2015 and Health-Related Quality of Life in Breast Cancer Patients Enrolled in a Randomized Trial. Cancers (Basel) 16. | Wrong population |
| 114 | Leach, M.J., Barber, G., Monacella, S., Jamieson, P., Trinh, T., Vo, N., Schmidt, U., Byrne, A., Ristevski, E., 2024. Adherence to dietary guidelines and associated factors among rural Australian cancer survivors: a cross-sectional study. Support Care Cancer 32, 326. | No diet quality indices used |
| 115 | Puklin, L.S., Harrigan, M., Cartmel, B., Sanft, T., Gottlieb, L., Zhou, B., Ferrucci, L.M., Li, F.-Y., Spiegelman, D., Sharifi, M., Irwin, M.L., 2023. Randomized Trial Evaluating a Self-Guided Lifestyle Intervention Delivered via Evidence-Based Materials versus a Waitlist Group on Changes in Body Weight, Diet Quality, Physical Activity, and Quality of Life among Breast Cancer Survivors, Cancers. | Wrong population |
| 116 | Grainger, E.M., Spees, C.K., Hill, E., Braun, A.C., Young, G., Mo, X., Webb, M.Z., Focht, B.C., Weiss, R., England, E.M., Clinton, S.K., 2024. A Phase II Trial of Beef Integrated into a Healthy Dietary Pattern for Cancer Survivors: Impact on Weight and Cardiometabolic Biomarkers. Current developments in nutrition 8, 102551. | No full text |

Wrong population: Median or mean age of population studied <65 years old
